# Community-based malaria control in southern Malawi: a description of experimental interventions of community workshops, house improvement and larval source management

**DOI:** 10.1186/s12936-018-2415-1

**Published:** 2018-07-16

**Authors:** Henk van den Berg, Michèle van Vugt, Alinune N. Kabaghe, Mackenzie Nkalapa, Rowlands Kaotcha, Zinenani Truwah, Tumaini Malenga, Asante Kadama, Saidon Banda, Tinashe Tizifa, Steven Gowelo, Monicah M. Mburu, Kamija S. Phiri, Willem Takken, Robert S. McCann

**Affiliations:** 1 Laboratory of Entomology, Wageningen University, PO Box 16, 6700AA Wageningen, The Netherlands; 2 Academic Medical Center, University of Amsterdam, Amsterdam, The Netherlands; 3 College of Medicine, University of Malawi, Blantyre, Malawi; 4 The Hunger Project, Blantyre, Malawi

**Keywords:** Community participation, Community workshops, Health education, House improvement, Integrated vector management, Larval source management, Malaria transmission, Vector control

## Abstract

**Background:**

Increased engagement of communities has been emphasized in global plans for malaria control and elimination. Three interventions to reinforce and complement national malaria control recommendations were developed and applied within the context of a broad-based development initiative, targeting a rural population surrounding a wildlife reserve. The interventions, which were part of a 2-year research trial, and assigned to the village level, were implemented through trained local volunteers, or ‘health animators’, who educated the community and facilitated collective action.

**Results:**

Community workshops on malaria were designed to increase uptake of national recommendations; a manual was developed, and training of health animators conducted, with educational content and analytical tools for a series of fortnightly community workshops in annual cycles at village level. The roll-back malaria principle of diagnosis, treatment and use of long-lasting insecticidal nets was a central component of the workshops. Structural house improvement to reduce entry of malaria vectors consisted of targeted activities in selected villages to mobilize the community into voluntarily closing the eaves and screening the windows of their houses; the project provided wire mesh for screening. Corrective measures were introduced to respond to field challenges. Committees were established at village level to coordinate the house improvement activities. Larval source management (LSM) in selected villages consisted of two parts: one on removal of standing water bodies by the community at large; and one on larviciding with bacterial insecticide *Bacillus thuringiensis israelensis* by trained village committees. Community workshops on malaria were implemented as ‘core intervention’ in all villages. House improvement and LSM were implemented in addition to community workshops on malaria in selected villages.

**Conclusions:**

Three novel interventions for community mobilization on malaria prevention and control were described. The interventions comprised local organizational structure, education and collective action, and incorporated elements of problem identification, planning and evaluation. These methods could be applicable to other countries and settings.

**Electronic supplementary material:**

The online version of this article (10.1186/s12936-018-2415-1) contains supplementary material, which is available to authorized users.

## Background

In the face of substantial advancements made in malaria control in sub-Saharan Africa since 2000 [1], there are strong indications that the active involvement of communities within malaria control and elimination programmes has not been fully realized [2, 3]. Community engagement is critical to the uptake of prevention, diagnosis and treatment, and helps elucidate how local knowledge, beliefs and practices affect the acceptability of interventions by the community [4, 5]. A review of studies on the impact of community participation concluded that the use of locally selected, and trained, volunteers was a common and important element of the success of interventions [2].

Now that bold new global targets have been set for further reduction of malaria case incidence and mortality by 2030 [6], a long-term commitment to community engagement is emphasized in the Roll Back Malaria (RBM) action plan that places malaria within the broader development agenda of the sustainable development goals (SDG), instead of a focus on disease alone [7]. Recommended interventions cover vector control, chemoprevention, diagnostic testing, treatment and surveillance. Insecticide-treated nets (ITNs) and indoor residual spraying (IRS) are core vector control interventions for endemic settings, but challenges with insecticide resistance and residual transmission (including outdoor transmission) suggest that supplemental interventions will be needed to enhance and sustain transmission reduction [8–10].

Structural house improvement and larval source management are existing vector control methods that have demonstrated an epidemiological impact in specific settings [11–14]. These methods have different modes of action from ITNs and IRS and, when they show incremental impact on disease, they could be implemented in addition to the core interventions. Active engagement of communities and local partners is considered appropriate for implementing house improvement and larval source management on a large scale.

In Malawi, malaria continues to be a major public health burden, with over 6 million presumed and confirmed cases reported in 2015 in a population of 17 million [15], although the malaria mortality rate has shown a steady decline over the past 15 years [16]. Insecticide-treated nets are the primary intervention for malaria prevention, but problems have been reported with net use and the development of insecticide resistance [17–21]. Moreover, challenges remain in adherence to case management policy and prompt treatment seeking [21, 22].

This paper describes three interventions that were implemented through trained community volunteers: community workshops on malaria, structural house improvement and larval source management. The interventions were part of a cluster-randomized controlled trial, described in a separate paper [23]. The descriptions of these intervention methods may inspire other vector-borne disease control initiatives in developing their community-based interventions.

## Methods

**Fig. 1:**
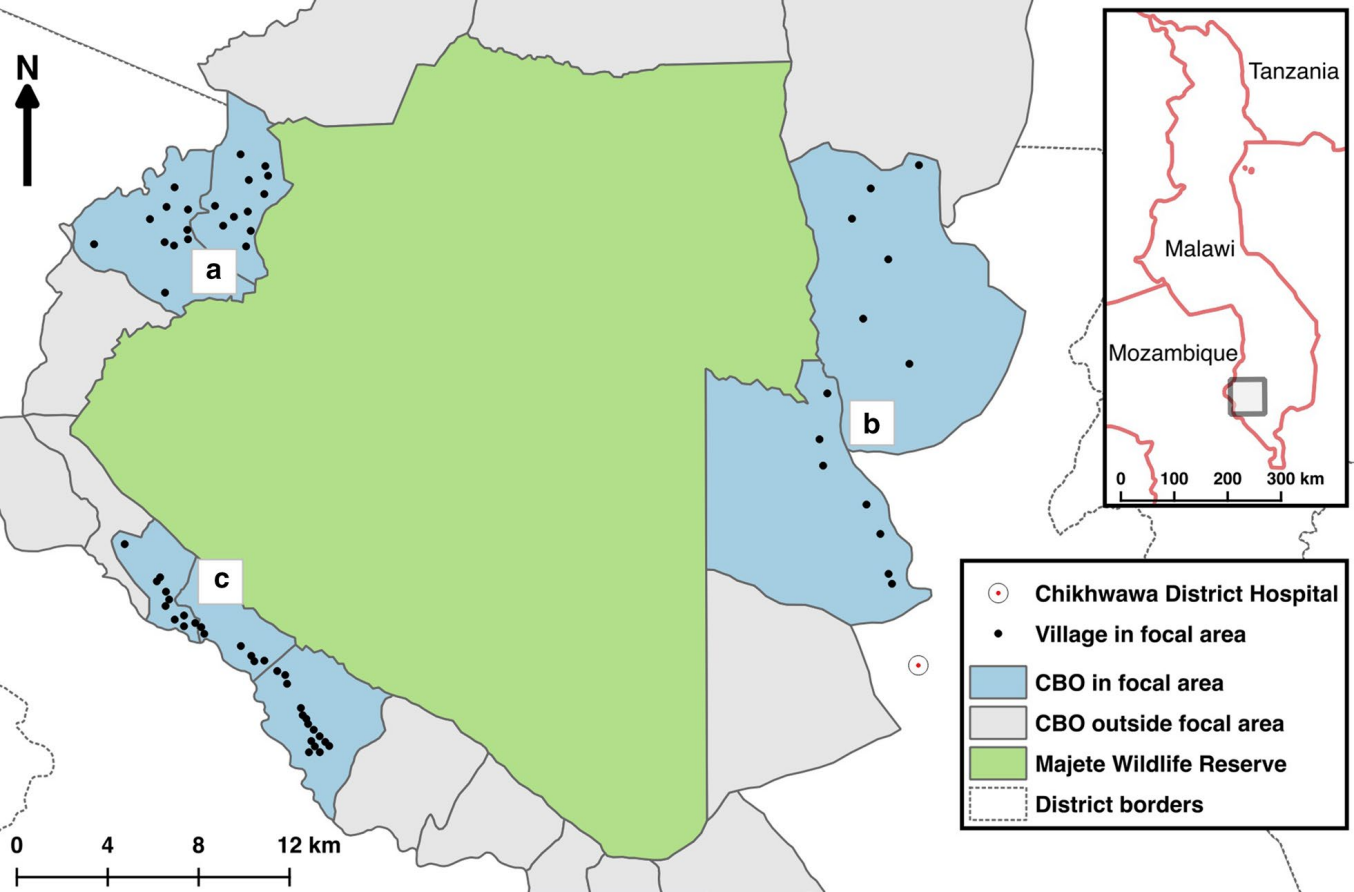
Study site map, showing Majete Wildlife Reserve, surrounded by 19 groups of villages known as community-based organisations (CBO). Three focal areas (A, B and C), each with their individual villages, are indicated (Reprinted with modifications [23, 49])

The study area is a zone, on average 10 km wide, surrounding the 70,000 ha of the Majete Wildlife Reserve in the Lower Shire Valley in Chikhwawa District, southern Malawi (Fig. 1). This zone, which is inhabited by approximately 90,000 people, will be referred to as the Majete perimeter. Within this zone, three focal areas (indicated as A, B and C), spaced roughly evenly around the perimeter were selected for the intervention trial [23]. These focal areas had a population of 24,153 (enumeration results as of 2014) living in 65 villages, including some recently split-up villages. Average household size was 4.5.

The objective of the trial was to determine the incremental impact of house improvement and larval source management on malaria parasite transmission, when added to nationally recommended interventions [23]. The trial was implemented from May 2016 through April 2018, preceded by a 1-year baseline period. After the end of the trial, the best interventions will be rolled out to the remainder of Majete perimeter. Out of the 65 villages covered by the trial, seven were allocated as ‘control’, twelve as ‘buffer’, thirteen as ‘house improvement’, 24 as ‘larval source management’, and nine as ‘house improvement plus larval source management’. Community workshops on malaria were implemented in all villages, as ‘core intervention’ to enhance uptake of nationally recommended tools and guidelines.

During the baseline data collection in 2015/16 preceding the trial, a 34% malaria prevalence rate and a clinical malaria incidence rate of 1.2 cases per child-year at risk in U5′s were measured in the three focal areas [24, 25], indicating high parasite transmission and burden. Malaria control is implemented by the District Health Office following national guidelines and coverage targets, using long-lasting insecticidal nets (LLINs) and IPTp for prevention, microscopy or malaria rapid diagnostic tests (RDT) for diagnosis, and artemisinin-based combination therapy (ACT) for treatment; however, these interventions are often challenged by field realities [23]. The District Health Office employs community health workers who were in 2015 upgraded and trained to conduct malaria diagnosis and treatment at community level.

From 2003, Majete Wildlife Reserve has, after a period of being devoid of most wildlife due to poaching, been rehabilitated, restocked and managed by African Parks Majete (Ltd), with the goal to develop an environmentally and financially sustainable park by 2028. A 144 km-long predator-proof perimeter fence was constructed to surround the entire reserve, and law enforcement officers and scouts patrol the Reserve to deter poaching [26]. In order to advance the role of the people living in Majete perimeter as partner in wildlife conservation, various initiatives for employment, education and development have been created during the past decade, through a series of community projects, and construction of schools, boreholes and health clinics.

The main development project in Majete perimeter is operated by international non-governmental organization The Hunger Project. This organization uses the so-called ‘epicentre’ strategy, whereby an epicentre is an introduced social infrastructure covering proximate villages with a 4000–20,000 population per epicentre. An epicentre actively engages with existing local government structures and services, and initiates a number of development programmes to address acute problems in rural livelihoods [27]. As of 2018, there are 5 existing and 3 planned epicentres in Majete perimeter. Four phases are identified in the development of an epicentre: (i) 1 year of activities to mobilize the community and establish thematic committees; (ii) a 2-year period in which a large epicentre building is constructed together with the community and during which development programmes are established; (iii) a 3-year period of implementation of development programmes; and (iv) a 2-year period to establish self-reliance of the epicentre, whereby external support is retracted but monitoring continues.

The Hunger Project uses participatory methods at village-level to initiate a change in people’s mindset—from resignation and dependency towards vision and commitment—to help overcome livelihood challenges. Selected villagers are trained to become ‘animators’, which are local volunteers who facilitate educational programmes, such as on nutrition and HIV/AIDS prevention, within their own village. Other epicentre development programmes each with their dedicated committee and core members include: water and sanitation, adult literacy, improved farming, food security, and microfinance. The epicentre building accommodates a health facility, nursery school, library, food processing room, microcredit bank, meeting hall, and sanitary facilities. The health facility, with outpatient department and often with maternity facility, is operated by the District Health Office, which provides personnel and commodities.

The research project took an early decision to embed its malaria control interventions within the ‘context’ of The Hunger Project’s epicentres, rather than implementing malaria control as a stand-alone package. The rationale was that the uptake of the malaria interventions could be enhanced through the community approach introduced by The Hunger Project. The project entered into a collaborative agreement with The Hunger Project and African Parks. Hence, malaria control effectively became an additional development programme of the epicentre. In accordance, the ‘animator approach’ was adopted, whereby ‘health animators’ facilitated activities on malaria in each village at regular intervals.

In total, 77 health animators (1 or 2 per village) were selected, covering the 65 trial villages. The selection was conducted by village leaders and guided by The Hunger Project using criteria of literacy skills, leadership potential and level of motivation. Health animators operated as volunteers, without remuneration, but during their training they received a standard upkeep cash allowance, and branded T-shirts, caps and bags; they also received a bicycle for attending coordination meetings [28]. The community health workers operating for the District Health Office in the focal areas were given orientation training on the project’s interventions and attended some of the training courses for health animators, so as to stimulate active linkages with health animators.

## Results

### Intervention 1: Community workshops on malaria

**Fig. 2:**
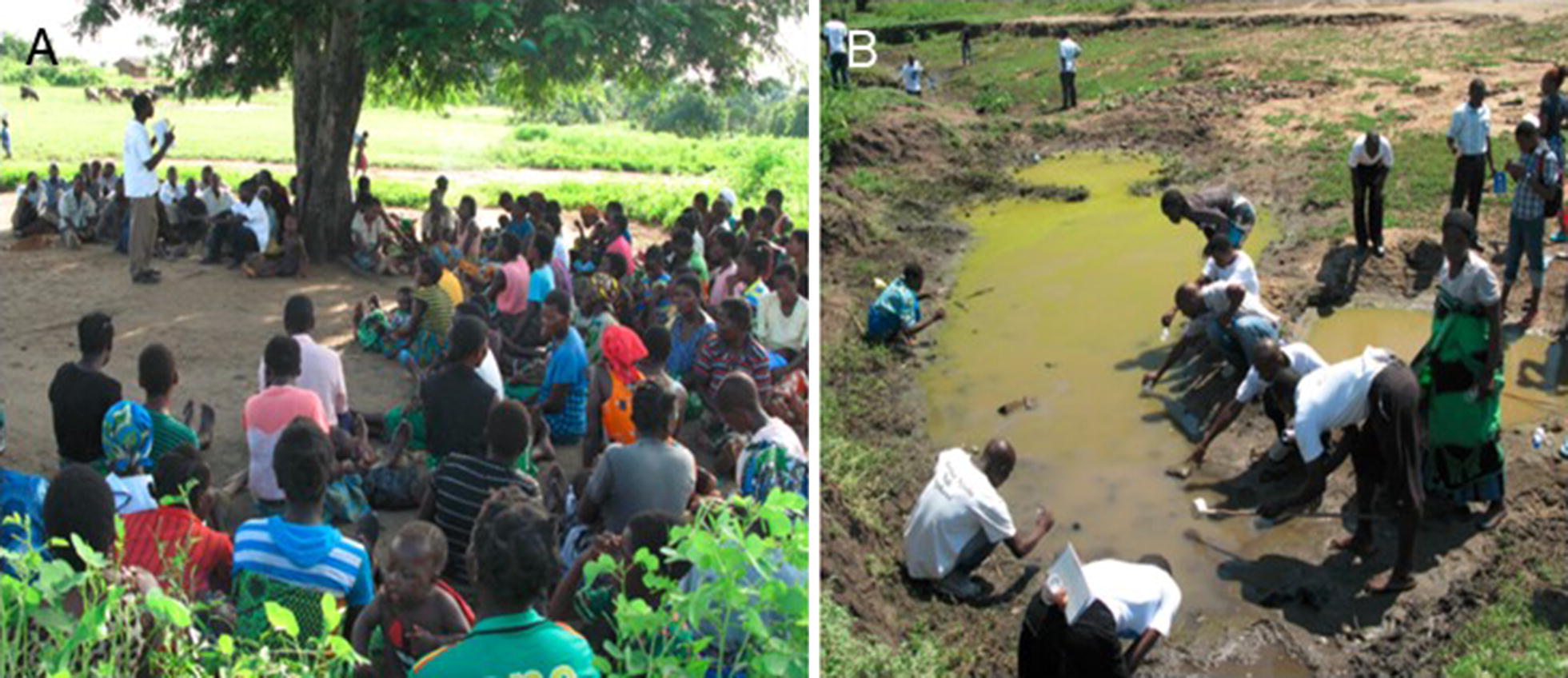
**A** Community workshop on malaria by health animators; **B** field training of health animators on larval source management. Photographs by H. van den Berg, Malawi, 2015 and 2016

Community workshops on malaria, led by trained health animators, were the core intervention implemented in all 65 villages covered by the trial (Fig. 2A). The objective of this intervention was to reach a meaningful proportion of the population with education, situation analysis and discussion on malaria prevention and control. The aim was to increase people’s compliance with nationally recommended malaria control measures and to encourage collective action. The intervention was through training-of-trainers, training of health animators, monitoring and support. The community workshops were delegated to the health animators.

In consultation with various stakeholders, and based on available information about the target population [29], a manual was developed with a number of topics designed for a series of fortnightly community workshops for an annual cycle (see Additional file 1); all project manuals were translated into the local language Chichewa. Visual aids were provided. Each community workshop session addressed a topic (Table 1), group discussion and self-reporting on malaria cases. Self-reporting on malaria cases involved the counting new cases since the previous workshop among participating households, and noting health services received; the results were then to be used in group discussion [28].

**Table 1:** Topics of community workshops led by health animators

Category	Workshop/topic^a^		Learning objective
A. Introduction	1	Vision and commitment on malaria	Change of mind-set with regard to malaria control
	2	Participatory evaluation	Able to evaluate current situation, regarding malaria prevention
B. Malaria basics	3	Malaria transmission cycle	Able to describe how the malaria parasite is transmitted
	4	Malaria signs and symptoms	Able to explain the signs, symptoms and burden of malaria
	5	Malaria diagnosis	Able to explain the importance of early diagnosis and treatment
	6	Malaria treatment and compliance	Able to explain importance of early treatment and compliance
	7	Malaria prevention	Able to outline the available strategies for malaria prevention
C. Bed nets	8	Biting behaviour of mosquito vector	Able to describe biting behaviour and self-protection measures
	9	Bed net hang-up	Demonstrate skills in proper hang-up of bed net
	10	Bed net distribution	Able to describe purpose and mechanism of bed net distribution
	11	Bed net use	Demonstrate skills in correct use of bed net to optimize protection
	12	Bed net maintenance and repair	Demonstrate skills in proper maintenance, repair, of bed nets
D. Various	13	Life-cycle and breeding of the vector	Able to describe mosquito life-cycle and breeding sites
	14	Recognizing the mosquitoes	Able to distinguish adult malaria mosquitoes
	15	Risks of contracting malaria parasite	Able to analyze local risk factors of infection
	16	Vulnerable groups	Able to describe vulnerability of pregnant women, young children
	17	Case management, severe malaria	Able to recognize severe malaria, need for hospital admission
	18	Community and health system	Able to explain health system structure and community linkage
	19	Mother and infant care	Able to explain role of mother to recognize severe illness in infants
	20	Malaria prevention (repeat)	See (7)
	21	Bed net maintenance, repair (repeat)	See (12)
E. Community	22	Community-wide malaria control	Able to explain need for collective action, identify players
	23	Problem analysis	Able to identify problems, causes and effects
	24	Community action planning	Able to develop a plan of collective actions to control malaria
	25	Songs and drama	Able to explain the role of songs and drama for awareness raising
	26	Participatory evaluation	Able to evaluate progress made, regarding malaria prevention

See Additional file 1

^a^The order of topics was adjusted to the local situation or demand

After a 2-day training-of-trainers, the health animators received a total of 7 days of training to build technical, organizational and group facilitation skills, using participatory methods. Also, drama and songs were developed and practiced on a range of issues, for example on the malaria transmission cycle, or in relation to the health system. Following the initial training September–October 2014, health animators received 3-day refresher trainings at the end of the year in 2015, 2016 and 2017. Health animators made initial arrangements with the village head and informed villagers about a launch event, coinciding with the first community workshop. Community workshops were then to be held at village level, or cluster village level, whereby the communities from one or, in some cases, more villages met in a central place, typically under a landmark tree.

Monitoring and support was given through monthly coordination meetings, conducted at focal-area level, under the leadership of an epicentre project officer. Participants were health animators, and sometimes also the village leaders and community health workers. Community workshops were also monitored through spot checks and self-monitoring.

The implementation and outputs of the community workshops have been reported in a separate contribution [28]. The results indicated feasibility of the malaria workshops with reasonable attendance of communities in the malaria workshops; 10–17% of the population participated per workshop (average 46 adult participants per workshop), with 23.5 workshops held per village per year. The community workshops were found appropriate and acceptable in rural communities, but the support from village leaders and community health workers was critical for success.

### Intervention 2: Structural house improvement

Structural house improvement was implemented in 22 selected villages of the trial (total of 2344 houses) in addition to intervention 1. Prior observations had indicated a high proportion of houses with eaves (including gables) and windows (including vent holes) being open, thus allowing entry of malaria vectors. The intervention consisted of targeted activities for mobilizing the community into making adequate house improvements; the project itself did not conduct house improvements.

The project provided locally-procured mosquito wire mesh and some basic hand tools; the community provided bricks, mud, wood, nails and tools. A manual was developed for a series of community workshops on house improvement, including learning units, community organizing and action planning, for implementation in 2015 (see Additional file 2A). Each community workshop session covered a specific topic (Table 2A) and group discussion. There was no material provision for the doors, but the curriculum encouraged villagers to improve the door or doorframe, and keep the door closed after dark.

**Table 2:** Topics of workshops on house improvement led by health animators

Phase	Workshop/topic		Learning/action objective
*A. Community workshops on house improvement (July*–*December 2015)*			
	1	Housing situation, brick baking	Prepare community for house improvement, plan brick baking
	2	Mosquito house entry behaviour	Able to explain mosquito behaviour and protection methods
	3	Methods of house improvement	Able to describe appropriate methods of house improvement
	4	Organizing and action planning	Prepare village-wide plan for house improvement
	5	1st evaluation of progress	Describe progress and plan further activities
	6	2nd evaluation of progress	Same as above
	7	3rd evaluation of progress	Same as above
	8	Maintenance of house improvement^a^	Motivated to maintain structural house improvements
	9	Closing door, windows after dark	Able to explain importance of closing door, windows after dark
*B. Establishing demonstration houses (August*–*September 2016)*			
	10	Meetings at focal area-, village-level	Plan activities and roles for Phase 2
	12	Establish demonstration house	Show example of properly sealed eaves to all villagers
	13	Village event	Launch campaign and plan activities ahead
*C. Training of village committees (April 2017)*			
	14	Identification of gaps and maintenance	Committees motivated and skilled to address shortcomings in HI

See Additional file 2

^a^This activity was added as a topic in regular ongoing community workshops on malaria

After a 1-day training-of-trainers, 29 health animators representing the villages selected for house improvement in the three focal areas took part in a 3-day training workshop in July 2015; half-day refresher courses were given in 2016 and 2017. During the training, health animators conducted a simple house survey, to understand people’s perceptions regarding house improvement, and a practical exercise to gain hands-on experience in developing best practices of house improvement. The training was interactive in that some outcomes of discussions were subsequently adopted in the training curriculum. The group decided that best practices of house improvement in the local setting were to begin with closing of eaves with bricks and mud, after which wire mesh would be made available to households for screening the windows. There was consensus that open eaves were generally not intended to provide ventilation, which was confirmed by a survey conducted in one of the focal areas, indicating 97% willingness to close eaves (Mburu, unpublished data, 2015). The group further decided that the best strategy for house improvement (HI) would be to establish a HI committee at village level, tasked with the distribution of wire mesh, coordination and monitoring. Committee members would be gender balanced and democratically elected, with the village head and community health worker having advisory roles.

**Fig. 3:**
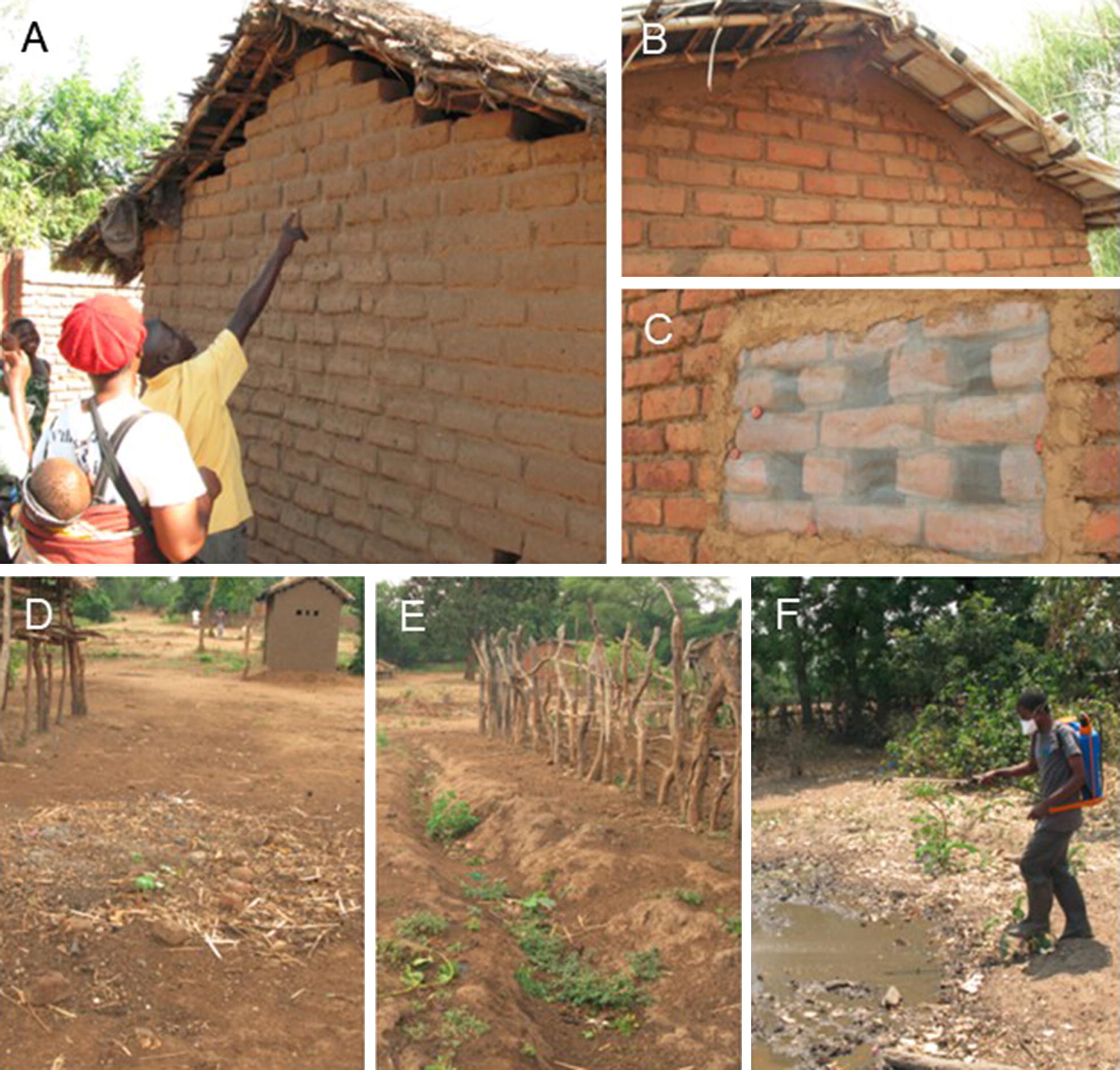
**A** House improvement pre-intervention; **B** closed gable; **C** screened ‘windows’; **D** former water body filled with soil; **E** drainage passage created to prevent standing water; **F** trained HI committee member applying *Bacillus thuringiensis israelensis* to remaining water bodies. Photographs by H. van den Berg, Malawi, 2015 and 2016

Immediately after the training, health animators organized a launch event in their respective villages to elect committee members (average 10/village) and initiate need-based brick making. Community workshops on house improvement were implemented at monthly intervals (Table 2A). In October 2015, progress monitoring suggested that the majority of households had made a voluntary effort to close their eaves, in response to which the wire mesh was distributed to the community (Fig. 3A–C). In May 2016, field visits indicated reasonably good implementation of window screening (including vent holes; Fig. 3C), but a poor quality of closure of eaves. In response, a plan for corrective voluntary measures was made (Table 2B; see Additional file 2B), which emphasized the preparation of demonstration houses in each HI village and the launch of the next stage in the house improvement campaign by HI committees. By September 2016, all villages had at least one demonstration house with properly sealed eaves and screened windows.

Concurrently, the wire mesh started to show signs of corrosion in some locations; a magnet-test confirmed that the mesh contained iron. By February 2017, corrosion had led to broken screens in a number of houses, implying the need for replacement. To address this challenge, 1-day training sessions were held covering all 22 HI committees, their health animators, community health workers and village leaders, to identify local gaps, and to discuss monitoring and response action by the committees (Table 2C; see Additional file 2C). In June 2017, field monitoring indicated that replacement of wire mesh with a non-corrosive aluminium mesh had been largely completed, with some exceptions. The quality of house improvement was measured using coverage indicators in randomly selected houses during the trial period, which will be reported separately.

### Intervention 3: Larval source management

Larval source management (LSM) was implemented in 33 selected villages of the trial, and in the 400-m buffer zone surrounding each selected village [23]; LSM was in addition to intervention 1. The intervention consisted of activities aiming to mobilize the community to conduct LSM; the project itself did not directly carry out LSM. In the context of the trial, LSM refers to removal or larviciding of larval habitats [23].

**Fig. 4:**
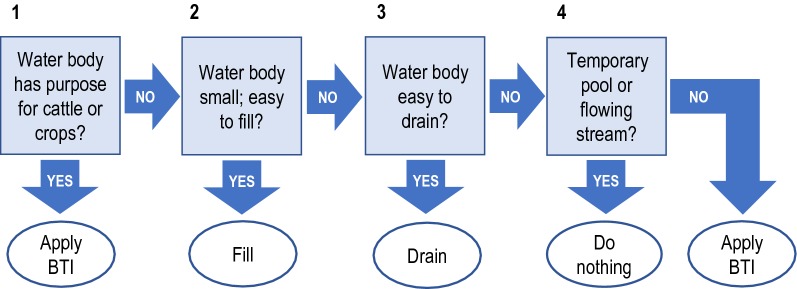
Guideline on the use of methods of larval source management

Removal of larval habitats (draining, filling) can be implemented by the community without external material support (Fig. 3D, E). However, larviciding depends on equipment and supplies that were provided by the project, namely manual compression knapsack sprayers, goggles, gloves, rubber boots, and a supply of the bacterial insecticide *Bacillus thuringiensis israelensis* (VectoBac WDG, Valent Biosciences, Libertyville IL, USA), referred to as ‘Bti’. Hence, the training was developed in two parts: the first part with focus on removal of standing water bodies by the community at large; the second part on larviciding with Bti by community representatives (Fig. 3F). A flowchart was developed to facilitate the efficient use of Bti (Fig. 4).

Regarding the removal of larval habitats, a manual was developed for a series of community workshops, including learning units and action planning (Table 3; see Additional file 3). In November 2015, after a 2-day training-of-trainers, 43 health animators representing the villages selected for LSM in the three focal areas took part in a 3-day training workshop; 1-day refresher courses were given in 2016 and 2017. The curriculum included practical sessions on identification of larvae of *Anopheles* and *Culex*, use of LSM planning tools (e.g. participatory mapping; community work plan), and field sampling of various larval habitats (Fig. 2B) to discuss options for control. As for house improvement, the health animators agreed on the need to establish LSM committees to run LSM activities in each selected village (in some cases, the committee covered several adjoining villages).

**Table 3:** Outline of topics of workshops on larval source management led by health animators

Category	Workshop/topic		Learning objective
Basics	1	Breeding of malaria mosquitoes	Understand larval breeding, establish LSM committee
	2	Collecting and recognizing mosquito larvae	Able to distinguish larvae of malaria mosquitoes
	3	Draining and filling of breeding sites	Understand methods of filling and draining
Planning	4	Exploring where mosquitoes breed	Mapping of breeding sites in the village
	5	Community organizing to remove breeding	Prepare a plan for village-wide LSM; agree on roles
	6	Killing mosquito larvae with Bti	Understand role of Bti to complement filling, draining
Action	7–12	Community action to reduce breeding	Bti team and villagers evaluate and plan activities

See Additional file 3

Regarding larviciding with Bti, a training manual was developed for LSM committee members, as community representatives who would do the spraying. The curriculum covered basics (e.g. surveying larval habitats), practical aspects (e.g. how to conduct a spray operation), and organizational and management aspects (e.g. record keeping) (Table 4; see Additional file 4). In May 2016, a 3-day training-of-trainers was succeeded by 3-day practical training sessions conducted in nine locations, to cover all 266 LSM committee members, their health animators, community health workers and village leaders. After these trainings, the LSM committees were monitored and supported regarding technical issues (e.g. preparing spray mix; systematic surveying). In addition, the monthly coordination meetings held in each focal area for intervention 1, provided a forum for discussion and support on LSM. The quality of LSM was measured by the project using coverage indicators, which will be reported separately.

**Table 4:** Outline of topics of training on Bti application for village committee members

Category	Session/topic		Learning objective
Basics	1	What is Bti?	Understand characteristics and role of Bti
	2	How to use the sprayer	Understand sprayer components and assembling
	3	When to use filling, draining or Bti	Learn to apply decision rules
	4	Surveying and mapping of breeding sites	Learn to use village mapping as planning tool
Spray operation	5	Preparing for a spray operation	Able to make all steps in preparation for spraying
	6	Conducting a spray round	Skilled in methods of spraying
	7	Cleaning up after spraying	Able to conduct proper clean-up
	8	Maintaining, storing equipment, supplies	Able to conduct basic maintenance of spray equipment
	9	Examining effectiveness of spraying	Able to evaluate effect of spraying on mosquito larvae
Organization, management	10	Roles of LSM committee	Aware of roles of the committee and its members
	11	Feedback at community workshops	Able to give feedback on Bti spraying to villagers
	12	Preparing, maintaining a work plan	Able to use a work plan of weekly activities
	13	Record keeping and reporting	Able to report to health animators and villagers

See Additional file 4

## Discussion

Recent studies on vector control have highlighted the value of community participation from various angles. Studies from Rwanda, Uganda and Kenya showed the critical importance of engaging communities in the formulation of appropriate measures towards malaria reduction [30, 31], and in the design of a suitable implementation strategy for a new vector control technology [32, 33]. Other studies demonstrated the community’s role in implementation of larval control. Surveillance and microbial larviciding of malaria vectors in Dar es Salaam, Tanzania, has been successfully implemented through trained, modestly-paid community members, recruited via the local government, resulting in reduced malaria prevalence [34, 35]. In Sri Lanka, farmer field schools in irrigated rice systems led to improved knowledge and an increase in environmentally sound mosquito control measures [36, 37].

The methods of community participation described in this paper resemble those of studies on dengue vector control, which comprised local organizational structure, education and collective action. In Cuba, community working groups of trained volunteers at the neighbourhood level engaged the community and stakeholders in decision making, leading to effective dengue vector control [38, 39]. In Vietnam, committees of volunteers at commune level received education and carried out surveillance and use of biological control agents, resulting in local elimination of dengue [40, 41]. Comparably, this paper describes methods of establishing ‘nodes’ at village level, whereby animators and committees educated the community on malaria prevention and control, and instigated village-wide action.

The economic and financial costs of the described interventions, the outcomes in terms of a change in knowledge, attitudes and practices, and impact on malaria prevalence and transmission, will be presented in separate publications.

There are several reasons why the described methods have prospect for increasing the community’s role in malaria control. The decision to operate through trained local unpaid volunteers (health animators; village-level committees) allowed the interventions to be carried out at scale, despite the time-consuming character of the methods (e.g. conducting community workshops). Moreover, the methods included elements that have been associated with community ownership and empowerment; these elements are: problem identification, adult education, priority setting, planning, action, and participatory monitoring and evaluation [2, 38, 42, 43]. For example, problem analysis was included as topic for community workshops on malaria, and a form of evaluation during community workshops was through self-reporting on malaria cases.

The interventions were introduced through the existing structures of local government and epicentres in order to foster acceptability and sustainability, whereby village leaders and community health workers played a supportive role. Also, the core intervention of community workshops provided momentum through fortnightly malaria-related activities at village level during annual cycles. The Hunger Project envisages to sustain the community workshops for epicentres that have become self-reliant, as a continuous forum for villagers to tackle malaria or other (emerging) health issues.

It has been advocated that strategies of vector-borne disease control need to be complemented by actions in other sectors to address the determinants of malaria, thus increasing effectiveness and sustainability [44–46]. The partnership with The Hunger Project and African Parks—agencies with long-term strategies that aim towards self-reliance and sustainability—had several benefits, as compared to a stand-alone malaria package. Harnessing the epicentre’s existing infrastructure with its mechanisms of support, feedback and non-fiscal incentives can render participatory malaria control interventions more effective, by raising motivation and volunteerism. Moreover, the epicentre’s development programmes, and income generated from tourism from Majete Wildlife Reserve, potentially improve local socio-economic conditions, thus complementing malaria control [47].

Despite these prospects for increasing the demand for, and uptake of, interventions by the community, the provision of health services in the targeted areas remains a challenge, with inadequate personnel, frequent stock-outs of anti-malaria commodities, and inconsistent case recording [48]. Consequently, systematic strengthening of the rural health services deserves urgent attention.

A limitation of the intervention methods described in this paper was that the investment in training and field support during the trial period was moderately costly. Nevertheless, it is planned that during the roll-out phase after the trial, in which all villages within the Majete perimeter will receive malaria interventions based on the trial’s outcome, inputs will be gradually reduced by handover of tasks of training, monitoring and field support from project staff to health animators, in line with the epicentre strategy, resulting in reduced expenses for personnel and transport. Regarding the provision of supplies, a system of local entrepreneurship could be developed, in which the community makes financial contributions for purchase of wire mesh or Bti. Also, future trials could consider the use of botanical insecticide products such as Neem oil (*Azadirachta indica*, Meliaceae) as locally-available alternatives to Bti.

There may be further options for using community-based interventions such as described in this paper—other than through a partnership with development agencies. One approach could be by expanding the reach of the health system, whereby local field staff are assigned with tasks similar to those of a health animator, under supervision of the community health worker. However, the question will remain to be studied whether the approach would be feasible if not imbedded within a broader development programme like the epicentre strategy.

## Conclusions

This paper described novel interventions for community mobilization on malaria prevention and control through involvement of local volunteers as health animators in 65 villages. The interventions were embedded within the context of a broad-based development programme, thus, harnessing existing mechanisms of support, feedback, non-fiscal incentives. The interventions incorporated elements of problem identification, planning and evaluation, which could lead to empowerment effects in individuals and communities.

Community workshops on malaria provided a platform for basic education on malaria, aiming to increase demand for and uptake of health services by the community. Interventions of house improvement and larval source management were introduced in addition to the community workshops on malaria, as two introduced methods with potential to complement national malaria control recommendations; both were implemented by the community.

## Additional files

**Additional file 1.** Training manual for health animators on malaria prevention and control.

**Additional file 2.** Training manual on house improvement for malaria prevention and control.

**Additional file 3.** Training manual for health animators on larval source management for malaria prevention and control.

**Additional file 4.** Manual on the use of BTI by village committee members for malaria prevention and control.

## Abbreviations

ACT: artemisinin-based combination therapy
Bti: *Bacillus thuringiensis israelensis*
HI: house improvement
IPTp: intermittent preventive treatment in pregnancy
IRS: indoor residual spraying
ITN: insecticide-treated nets
LLIN: long-lasting insecticidal nets
LSM: larval source management
RDT: malaria rapid diagnostic test
NMCP: National Malaria Control Programme
RBM: Roll Back Malaria Partnership
SDG: sustainable development goals
WDG: water dispersible granule
