# Supplementary material for: Community-based malaria control in southern Malawi: a description of experimental interventions of community workshops, house improvement and larval source management
Source: Malar J. 2018 Jul 16;17:266. doi: 10.1186/s12936-018-2415-1 (PMC6048888; doi:10.1186/s12936-018-2415-1)
Supplement: Supplementary file 1 — Additional file 1. Training manual for health animators on malaria prevention and control. [file 12936_2018_2415_MOESM1_ESM.pdf]

Additional file 1

TRAINING MANUAL
FOR HEALTH ANIMATORS
ON MALARIA PREVENTION
AND CONTROL

Majete Malaria Project

Majete Malaria Project – Community-based malaria control in the perimeter of Majete Wildlife Reserve

Contents

PREFACE **3**

INTRODUCTION **4**

PART I. ORGANIZING AND PLANNING COMMUNITY WORKSHOPS **7**

1. HOW TO ORGANIZE AND PLAN **7**
- 1.1 Local coordination 7
- 1.2 Preparatory activities 9
- 1.3 Leadership and facilitation skills 11

2. HOW TO CONDUCT COMMUNITY WORKSHOPS **13**
- 2.1 Workshop agenda 13
- 2.2 Record keeping 15

PART II. TOPICS FOR COMMUNITY WORKSHOPS **16**

3. GENERAL TOPICS **17**
- 3.1 Vision and commitment on malaria 17
- 3.2 Participatory evaluation 17

4. MALARIA BASICS **19**
- 4.1 Malaria transmission cycle 19
- 4.2 Malaria signs and symptoms 20
- 4.3 Malaria diagnosis 22
- 4.4 Malaria treatment and compliance 24
- 4.5 Malaria prevention 26
- 4.6 Vulnerable groups 28

5. VECTORS AND THE ENVIRONMENT **30**
- 5.1 Biting behaviour of the malaria mosquito 30
- 5.2 Life-cycle and breeding of the malaria vector 32
- 5.3 Recognizing the mosquitoes 33
- 5.4 Risks of contracting malaria parasites 34

6. PERSONAL PROTECTION FROM MALARIA **35**
- 6.1 Bed net hang-up 35
- 6.2 Bed net distribution 37
- 6.3 Bed net use 39
- 6.4 Bed net maintenance and repair 41

7. HEALTH CARE **43**
- 7.1 Case management and severe malaria 43
- 7.2 Community and health system 45
- 7.3 Mother and infant care 47

8. COMMUNITY ACTION **49**
- 8.1 Community-wide malaria control 49
- 8.2 Problem analysis 50
- 8.3 Community action planning 51
- 8.4 Theater for development, songs, in malaria prevention and control 53

ANNEXES **54**
- Annex 1. Participants list 55
- Annex 2. Self-reporting on malaria 57
- Annex 3. Malaria-adapted VCA manual 58

PREFACE

This manual has been developed by the Majete Malaria Project, as a collaborative effort between its project partners. Drafting of the document was by Henk van den Berg, with inputs and comments provided by Rob McCann, Zinenani Truwah, Rowlands Kaotcha, Mackenzie Nkalapa, Alinune Kabaghe, Michele van Vugt, Tumaini Malenga, Grace Chikowi, Henry Chungu, Samuel Kamoto and Tobias Kunkumbira. Annex 3 was produced separately by The Hunger Project.

This manual was first produced in October 2014, and has been adapted for use in subsequent years. The manual is available in the English and Chichewa language. Funding for development of this manual was provided by Dioraphte Foundation, the Netherlands. Requests for permission to reproduce or translate this document – whether for sale or for non-commercial distribution – should be addressed to the programme manager (e-mail address: pmthrue@medcol.mw).

INTRODUCTION

Malaria is one of the most serious health problems affecting the communities surrounding Majete Wildlife Reserve. Malaria causes immense human suffering, compromises economic development, and is a major burden on the health system.

The Hunger Project (THP) in collaboration with **Majete Malaria Project** (MMP) aim to drastically reduce the burden of malaria in the coming years. This will be done by strengthening the health system, rolling out effective interventions, and educating the communities through use of '**Health Animators**'. The health system will be supported through strengthened capacity and reliable supply of malaria diagnostic kits and medication at clinics and health posts.

The households and communities in each village have a major role to play in prevention and control of malaria. However, to achieve this, the communities need to be educated and empowered to make a change in their **mindset, actions and behaviour** in relation to malaria.

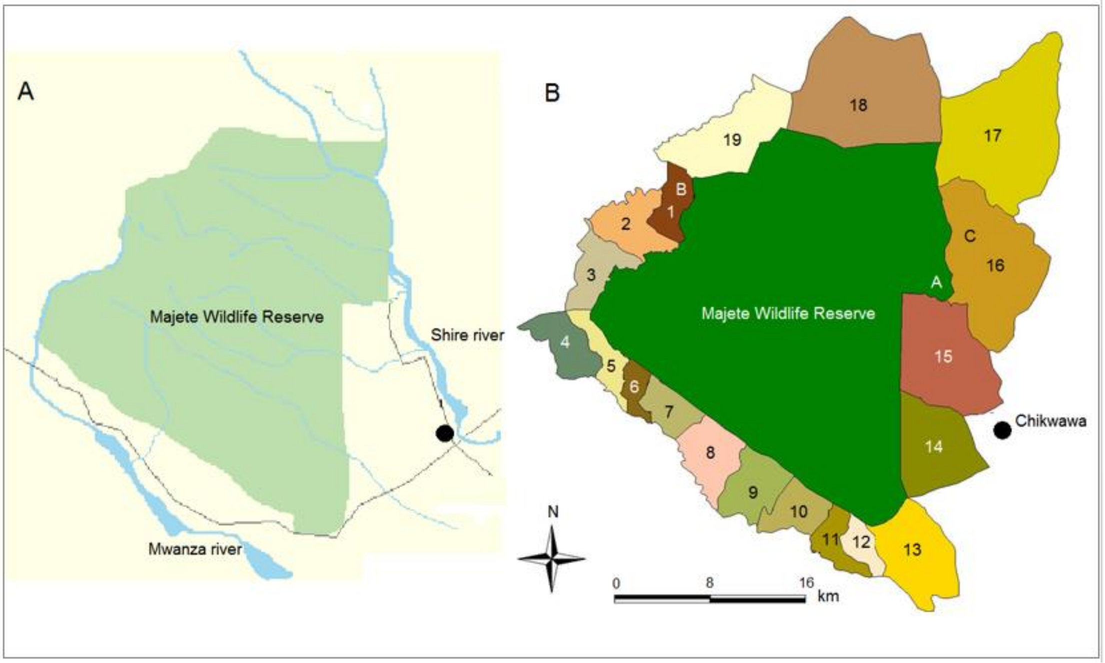

Figure 1. Map of Majete Wildlife Reserve and the Majete Perimeter

Health animators are an essential link between the project and the community. Their task is to enable the community to voluntarily **adopt interventions** and change their **practices and behaviours** in order to control malaria. Without the active role of health animators, the malaria control interventions cannot achieve sustained impact.

In this multi-partner activity, **THP-Malawi** is responsible for recruiting the health animators, managing the health animator group on the ground, and training of the health animators in the conduction of village workshops.

The **Health and Education Unit** (HEU) of MoH is responsible for assisting the development of information, education and communication (IEC) materials, assisting the training of health animators, annual supervision of the health animator process and health promotion policy.

The College of Medicine is responsible for training of the health animators on technical malaria information.

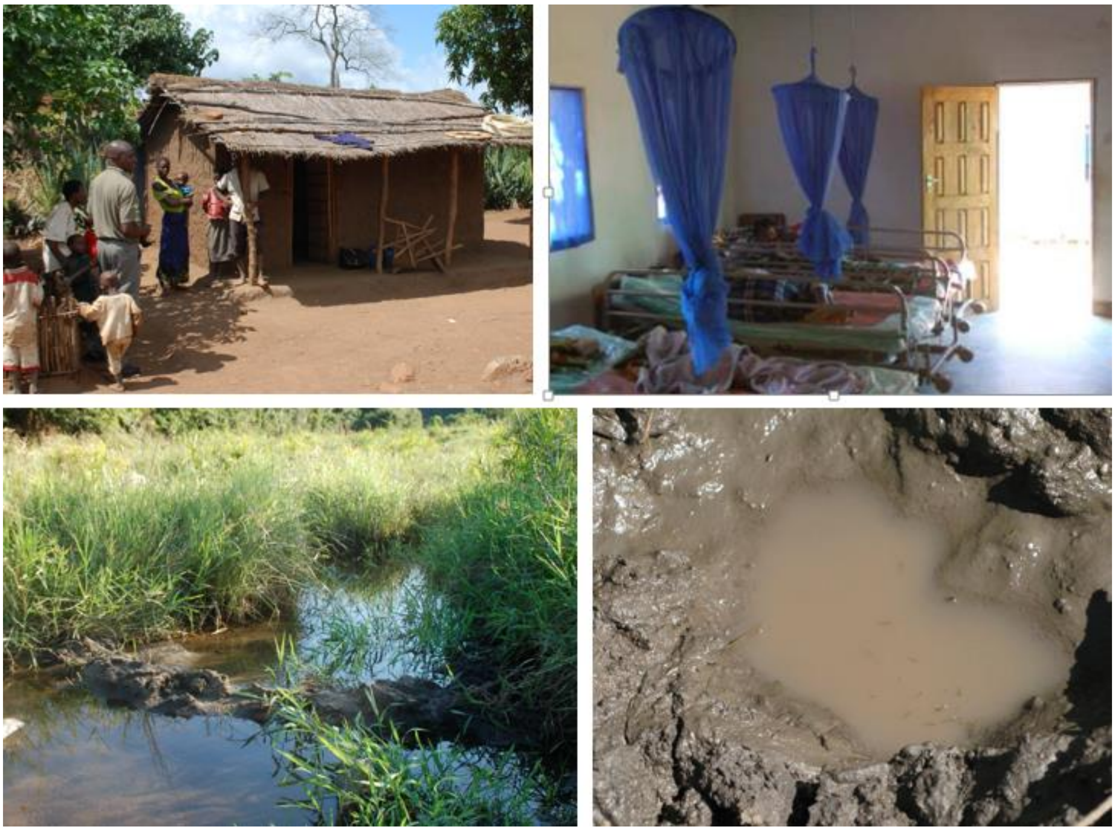

Figure 2. Houses around Majete; health centre; small and large breeding sites of malaria mosquitoes

Objective and goal

The objective of this curriculum is to provide villagers with optimal **education on malaria prevention and control**.

The ultimate goal of the malaria educational activities is to increase participation and **adoption** of malaria prevention and control interventions by household members of the targeted communities, thereby facilitating **collective action** against malaria at village level.

Educational approach

It is assumed that the villagers have limited educational background and low literacy levels, but that some of the villagers will have reading and writing skills.

The curriculum is divided into a number of short community **workshops**, each with a **specific learning objective**. The community workshops cover a wide range of topics in relation to malaria, from community perception, the basics of the disease to organizing collective action against malaria. Some key aspects are repeated in several workshop sessions.

At the onset, the **participants should commit** to attending all or most of the community workshops, because the sequence of topics discussed in the workshops constitute one **training course**.

Participatory evaluation and graduation introduce an evaluative and **reward mechanism**: Through their own evaluation at the beginning and the end of the year, participants will be able to see progress.

Visual materials (live specimens of mosquitoes in transparent cups, laminated colour materials, demonstrations) will be used to illustrate training content.

Text will be used sparingly because of low literacy levels.

The training content **introduces the key messages** which should be understood clearly by the villagers. **Group discussions** will circle around these key messages and myths will be debunked.

Role of health animators

The health animators are drawn from the villages. Therefore, they have a dual role: they **represent the project** as well as the community. Health animators are in fact the interface between project and community. This is essential, because the health animators will be the continuing factor in community-based malaria control, even after the project has come to an end.

Health animators mainly have a **role as facilitators**, who ensure that the learning process takes place and actively involve the villagers in planning, implementation and evaluation. Therefore, the model of "training-as-you-were-trained" is used, with clearly packaged sessions and use of the same manual for TOT and at village level.

The duration of the **training of health animators** is limited to 7 days (2 days of general training in skills for community engagement processes and 5 days of malaria training). During their training, the health animators will receive more in-depth information about the key messages related to each topic. Equally important, they will also learn about the community engagement processes to be used.

Health animators receive a refresher training once every year.

Use of this manual

This manual is used in the **training of health animators** on how to conduct community workshops on malaria. This manual is also used for **conducting the community workshops**.

Part I of the manual addresses general aspects of organizing and planning the community workshops. **Part II** presents the topics for discussion in the community workshops.

PART I. ORGANIZING AND PLANNING COMMUNITY WORKSHOPS

1. HOW TO ORGANIZE AND PLAN

1.1 Local coordination

Health animators should understand the importance of local coordination in malaria control activities. It is important to first determine the geographic **area covered** by the community workshops, and whether the health animator of one village will or will not **work together** with the health animator of the neighboring village. It is also important to understand the role of the **village head** and local **committees** in the malaria control activities, and to understand the need for **coordination meetings** with other health animators and the HSA's (community health workers) in the entire focal area, covering many villages.

Below is a **stake-holder analysis** for the malaria project; this will be updated when needed (Figure 3).

IMPORTANCE OF STAKEHOLDER ------------>

INFLUENCE OF STAKEHOLDER <------------

	**HIGH**	**LOW**
**HIGH**	**Key players: Village head, health committee, HSA, EPO, any committees of other malaria initiatives**<br><br>Action: Focus effort on this group. Engage and consult regularly, make deliberate initiative to involve them in some decisions as well as follow-ups on implementation of agreed action. Meet with them regularly. Ensure they are part of the monthly cordination meetings, with the possible exception for village heads	**Who: Village heads committee**<br><br>Action: Regularly inform this group about the malaria activities. Individual village heads are considered key players in their own villages
**LOW**	**Who: Epicenter committee, CBO leadership, DHO, Hospital staff of nearby hospital**<br><br>Action: Maintain a fair level of contact. Update them i.e request to brief them quarterly during their meetings. They are important as an executive committee of the epicenter and may be malaria 'ambassadors'	**Minor players: Other committees i.e. on food security, microfinance, education, water, etc.**<br><br>Action: Simply inform this group when chance for briefing arises

Figure 3. Stakeholder analysis of the Majete Malaria Project at field level

Implementation unit

The implementation unit for a community workshop is the geographical **area covered** by the workshop. The workshop is held for a small cluster of several neighboring villages. Hence, the implementation unit is the **village cluster**.

In a village cluster, there will most likely be more than one health animator. In case of more than one health animator per implementation unit, the health animators will **work together as a team** in organizing and conducting the community workshops. For example, one animator could be the main facilitator of the workshop whereas the other could be the rapporteur, or assist in demonstrations of presentation of visual materials to the workshop participants.

This will promote team work and enable them to **cover up** for each other, for example, when one health animator is not available or incapacitated. So in most cases animators of the same cluster conduct the workshops together in villages of their cluster (they may rotate the villages so that the workshops ideally happen in all the cluster villages).

In focal areas where an epicentre project officer (**EPO**) or equivalent is present, the EPO may attend a village workshop, but it is not imperative that the EPO should attend all village workshops.

Village head

The village head is a **key person** in the activities on malaria control. He or she represents the local leadership, and his **support in coordination, organization and planning** on community-level activities is of utmost importance.

The project provides an opportunity for the village head to **strengthen** his leadership position through his involvement in bed net distribution, communication activities, workshops and meetings of the existing committees.

For all community workshops and meetings on malaria control, the health animator should always timely **inform** the village head and **request his participation**.

Local committees

Several local committees might exist that have a bearing on the malaria control activities. These are the health committee, the village heads committee and the epicentre (or focal area) committee. Depending on the area, these committees may already exist or still have to be established by The Hunger Project. Where an epicentre is already present, there may be more committees related to specific development programmes, but those committees are of lower priority to malaria control.

The **health committee** is a group of representatives of local society that have a say in the functioning of the local health system. This committee can help ensure that the demand for health services is matched by the provision of services by the health system. Health committee members should be invited to **attend** community workshops on malaria. This will help improve the linkage between community and the health system.

The **village heads committee** is a committee that will be facilitated with the aid of the Hunger Project. This committee will consist of the village heads in the focal area (or epicentre). It is important that village heads within the focal area **meet regularly** to share experiences on malaria control and jointly address common problems.

The **epicentre committee** (or focal area committee) is another committee which will be established by The Hunger Project that will have an important role in the **overall development** of the area. This committee will consist of representatives of society. Whenever health animators are conducting village workshops, they should in advance extend an **invitation** to a member of the epicenter committee who comes from the cluster in which the meeting will be conducted.

Coordination meetings

In each focal area (or epicentre), the health animators will meet at a fixed day, time and venue **once a month**. Led by their coordinator (the EPO or equivalent), and the HSA, the health animators **report** on status of community workshops, problems encountered, and remedial action taken. The coordinator provides **feedback** by reporting the findings of monitoring visits and/or mentoring activities. THP and MMP **project staff** will frequently – at least twice per year – attend these coordination meetings to provide technical inputs and to monitor progress of activities.

1.2 Preparatory activities

Venue

It is important to have a **fixed** venue and time or date of the month – for example: *“every other Tuesday at 2:00pm at the village headman’s bwalo”* for the community workshops, so that the villagers know where and when to meet. A suitable venue should be selected, together with the village head. The venue should fulfill the following **criteria**:

- Easy access by villagers
- Possible to display newsprint presentations and bed net demonstrations
- Sufficient capacity
- Peaceful location with limited distractions

Preparing materials

In preparation of the community workshops, the health animator should have the **required materials** ready in advance of the workshops. The materials are indicated in Part II of this manual.

Launch

After the health animator has made coordinating arrangements together with the village head, preparations should be made for the launch of the community workshops. This is conducted by advertising or informing villagers about the new activity. This could be done by **door-to-door visits** by the health animator and/or by **announcements** at public events using a Megaphone. Care must be taken by the health animator that **far-out** households belonging to the village are not left out.

Annual plan

The workshops will be at **14-day intervals** throughout the year.

The community workshops will cover a wide **range of topics**, which are presented in Part II. It is important that **all topics** are covered during the year in an appropriate **sequence**.

Therefore, the health animator should carefully plan the topics for the year. The plan should fix the dates for workshops for the entire year. A provisional plan is presented below; this should be adjusted to field realities.

Table 1. Proposed plan for the topics of workshop sessions in an annual cycle. Chapter numbers are indicated for easy reference.

Block	Worksh.	Time	Course content	Manual chapter
Block 1: Introduction	1	1st month	Vision and commitment on malaria	3.1 General aspects
	2	1st month	Participatory evaluation (before training)	3.2 General aspects
Block 2: Malaria	3	2nd month	Malaria transmission cycle	4.1 Malaria basics
	4	2nd month	Malaria signs and symptoms	4.2 Malaria basics
	5	3rd month	Malaria diagnosis	4.3 Malaria basics
	6	3rd month	Malaria treatment and compliance	4.4 Malaria basics
	7	4th month	Malaria prevention	4.5 Malaria basics
Block 3: Bed nets	8	4th month	Biting behaviour of the mosquito vector	5.1 Vectors and environment
	9	5th month	Bed net hang-up	6.1 Personal protection
	10	5th month	Bed net distribution	6.2 Personal protection
	11	6th month	Bed net use	6.3 Personal protection
	12	6th month	Bed net maintenance and repair	6.4 Personal protection
Block 4: Various	13	7th month	Life-cycle and breeding of the vector	5.2 Vectors and environment
	14	7th month	Recognizing the mosquitoes	5.3 Vectors and environment
	15	8th month	Risks of contracting malaria parasites	5.4 Vectors and environment
	16	8th month	Vulnerable groups	4.6 Malaria basics
	17	9th month	Case management and severe malaria	7.1 Health care
	18	9th month	Community and health system	7.2 Health care
	19	10th month	Mother and infant care	7.3 Health care
Block 5: Community	20	10th month	Community-wide malaria control	8.1 Community action
	21	11th month	Problem analysis	8.2 Community action
	22	11th month	Community action planning	8.3 Community action
	23	12th month	Songs and drama	8.4 Community action
	24	12th month	Participatory evaluation (after training)	3.2 General aspects

To simplify the planning process, the workshops are divided into training '**blocks**'. Block 1 is the introduction of the community to malaria control activities. Block 2 relates to bed nets. Block 3 relates to vectors. Block 4 discusses the basic aspects of malaria. Block 5 covers various topics on malaria. Block 6 covers the role of the community in sustainable malaria control.

Bed net distribution

The **timing** of bed-net distribution is of major importance to the planning of the topics of community workshops. Therefore, the activities of Block 2 could be advanced or delayed according to the **actual availability of the nets**, as indicated in Table 1. Information on the availability of bed nets will be conveyed to the health animators by the HSA's during the monthly coordination meetings.

1.3 Leadership and facilitation skills

The health animator, as key person in conducting the community workshops, should develop the necessary **skills** for leadership and facilitation.

Leadership

Leadership is the ability to take the lead in getting the community organized. A leader is **not a 'boss'**, but rather a person who is willing to **serve the community**.

Good leadership skills are:

- Ability to clearly communicate
- Having confidence
- Committed to malaria control
- Positive attitude to improving the situation for all villagers
- Mindful of marginalized households or groups
- Able to involve others in organizing activities (the leader does not need to do everything him/herself)

Health animator as facilitator, not lecturer

The health animator plays the role of a facilitator, not a lecturer. The health animators should clearly understand the differences between a skilled facilitator and a lecturer.

Lecturers are needed in the formal educational system for teaching of pupils. However, for programmes on malaria and community development, the issues are directly related to people's lives. Also, the participants will include mostly adults. Therefore, **facilitation is more appropriate** than lecturing. There is a clear difference between facilitation and lecturing.

Lecturing aims to deliver information and instruct what should be done. Participants are the objects who should listen and do as told. The information is **passively** transferred from the lecturer to the pupils. Lecturing is suitable for children but not really for adults. The transferred information is quickly forgotten by the recipients.

Facilitation, on the other hand, aims to introduce the basic concepts but then allow the participants to use those concepts and **apply them to their own lives**. The facilitator should encourage the learning process by asking questions that help the participants find the solution. Unlike a lecturer, the facilitator does not immediately give the answer or solution, but allows the participants to come up with the answer or solution by themselves. Facilitation helps **bottom-up planning**, whereby the community learns to take an active role in the planning process.

If the facilitator gives a short-cut solution or answer without allowing the participants to come with the solution by themselves, this is a **missed opportunity for learning** by the community. A good facilitator does not give endless speeches, does not boast with his knowledge, but puts the **community on the foreground**, asks many questions, and gives corrective comments when needed.

The advantage of facilitation is that it generates more interest from the community by encouraging programme '**ownership**' by the community. Facilitation takes more time than lecturing but is much more effective. Facilitation allows the community to **actively participate** in malaria control and in planning activities to improve the situation of their livelihood.

2. HOW TO CONDUCT COMMUNITY WORKSHOPS

2.1 Workshop agenda

A typical community workshop would have agenda items as described in table 2.

Table 2. Proposed agenda items for community workshops; total duration approx. 2 hr.

No.	Workshop agenda item	Min	Notes	Self monitoring
1	Opening remarks and workshop agenda	10		Has the agenda been explained?
2	Introducing participants	10	Rapporteur completes participant record sheet	Are new participants introduced?
3	Summary of previous workshop	5		Is summary of previous session given?
4	Course content	30	See course content per session*	Are the key points presented clearly?
5	Group discussion	30	See questions related to course content*	Does a lively group discussion take place?
6	Self-reporting on malaria	15	Rapporteur updates record sheet on mala-ria cases and health services received	Is record keeping conducted correctly?
7	Plan for next workshop, and closing	5	Create expectation and encourage participation	Is next session's topic announced?

* See course content according to the schedule in table 1; course content is described in Part II

Where an epicentre has already been established, the malaria workshop will most likely be **merged** with the workshops of other epicentre programme. This may reduce the actual time available for the workshop session on malaria. The **minimum time** allotted to the malaria programme in a community workshop should be around 1 hour.

Opening

The workshop starts with greetings, prayer, followed by opening remarks and presentation of the agenda for the workshop. Participants should be asked whether they agree with the agenda.

Introduction of participants

Participants are asked to introduce themselves. This '**breaks the ice**', giving the participants a brief moment to talk in public, and this also stimulates group building. After a few workshop sessions, repetitive introductions may no longer be desirable, and the animator may decide to ask only the new participants to introduce themselves.

The animator should appoint a **rapporteur** to assist him/her in taking notes. The rapporteur should complete the participants list. An example of a participants list is presented in Annex 1. Keeping track of the participation is an important part in evaluating the attendance and commitment by individual villagers. This information will be used for the **accreditation** at the end of the first year, to determine whether participants will receive a graduation certificate on the topic of malaria control. Only those who have attended 18 or more workshops by the end of the year, will be granted a certificate.

Summary of previous workshop

The health animator briefly mentions the topic of the previous workshop, highlighting a few of the key points. This serves as a **refresher** for the participants and also helps any **newcomers** with understanding the context of the current workshop topic.

Course content

Next agenda item is the course content. This is the content as described in Part II of this manual. The topic of each workshop session is selected in accordance with the **plan** made for the year, as explained in Table 1. Approximately 30 minutes are allocated for explaining the course content; some topics may require more time. The rapporteur should be the timekeeper.

Group discussion

After the course content is clearly explained, the health animator should stimulate group discussion by asking the questions proposed in the manual. Remember that the purpose of the discussion is to stimulate **learning** and create programme **ownership**.

Avoid the situation that few persons dominate the group discussion; **invite** those that keep to the background to express their opinion. Ensure that the discussion does not divert from the scope of the topic.

End the discussion by summarizing the **main outcomes** and why this is important for malaria control.

Self-reporting on malaria

After the group discussion, the participants are asked to self-report on:

1. Malaria cases
2. Health services received

The record sheet is provided in Annex 2.

The health animator asks the participants to indicate the number of **new malaria cases** that have occurred in the households of the participants since the previous workshop. Cases that have already been recorded in the previous workshop, should not be included again: only the new cases should be counted. The total number is written in the table. A distinction is made between children (under 16 years) and adults (16 or above). The health animator discusses the **trend** in malaria cases by comparing the current cases with those recorded during previous workshops.

The health animator then asks whether these cases sought care from the health system. First, the **total number of cases** that sought health care is recorded. Then, the number of cases is recorded on which an **RDT test** was **done**. Then, the number of cases that **tested positive in the RDT** test is recorded. Then, the number of cases that **received LA** treatment is recorded (See Annex 2).

The health animator should discuss the data in relation to those recorded in previous workshops. The data can provide the villagers with interesting insights. For example, if most of the RDT's that were done produced a negative result, this means that there are many cases with related signs and symptoms, which will require further medical attention. Also, if the number of cases that received LA exceeds the number of cases that tested RDT positive, this means that LA is over-administered (given to patients

that should not have received it). The community should be aware that LA should only be given upon a positive RDT.

The records should be kept for the entire year, so that the **trend** can be analysed at the end.

Plan for next workshop

As a final agenda item, briefly present the plan for the next workshop with the topic for discussion. This will create **anticipation** from the villagers.

2.2 Record keeping

Record keeping is important for the purpose of **monitoring and evaluation**. The health animator, with the assistance of a rapporteur, should make records during each community workshop to keep track of the participation. In addition, malaria case reporting is conducted in each community workshop as a form of self-reporting by the villagers. Self-reporting is an important form of **participatory evaluation**. It provides the villagers with a feedback mechanism to monitor the trend in malaria cases. It also allows the community to evaluate the quality of health services, which helps generate the demand for quality health services by the community.

Hence, record keeping is needed on:

1. Participation list
2. Malaria cases
3. Health services received

Keeping accurate records of participants at each community workshop has two purposes. It assists the health animator in identifying households that **require door-to-door visits**, and it **stimulates routine participation** by villagers. The participant lists will also benefit the external monitoring of activities. The participants list is kept by the rapporteur. An example of a participant list is provided in **Annex 1**.

Self-reporting on malaria cases and health services received is recorded on the record sheet in **Annex 2**. The records on health services received should be regularly **shared** by the health animator with the **health committee** and with the **HSA**, for comparison vis-à-vis the HSA's own records.

All record sheets should be safely kept in a folder by the health animator. These data sheets should be **shared with the malaria project teams** during their monitoring visits to the village.

PART II. TOPICS FOR COMMUNITY WORKSHOPS

In this part of the manual, the topics of the community workshops are presented. The topics are divided into **six categories**: general topics; malaria basics; vectors and environment; health care; personal protection; and community action.

Table 3. Topics for community workshops

Category	Topic
General topics	3.1 Vision and commitment on malaria
	3.2 First participatory evaluation
Malaria basics	4.1 Malaria transmission cycle
	4.2 Malaria signs and symptoms
	4.3 Malaria diagnosis
	4.4 Malaria treatment and compliance
	4.5 Malaria prevention
	4.6 Vulnerable groups
Vectors and environment	5.1 Biting behaviour of the mosquito vector
	5.2 Life-cycle and breeding of the mosquito vector
	5.3 Recognizing the mosquitoes
	5.4 Risks of contracting malaria parasites
Personal protection	6.1 Bed net hang-up
	6.2 Bed net distribution
	6.3 Bed net use
	6.4 Bed net maintenance
Health care	7.1 Case management and severe malaria
	7.2 Community and health system
	7.3 Mother and infant care
Community action	8.1 Community-wide malaria control
	8.2 Problem analysis
	8.3 Community action planning
	8.4 Songs and drama

3. GENERAL TOPICS

3.1 Vision and commitment on malaria

Participants may have been lived in an environment where malaria-related deaths and malaria itself may have now been accepted as part of life - if not disguised as a curse or something that is God-given and humans cannot influence. The purpose of this section is therefore to challenge participants to envision a possibility of a new future, free from malaria and malaria-related deaths.

This is an active process and may require the facilitator to engage the participants in seemingly feared discussions that “it is possible to eliminate malaria”. Like any other general VCA, the discussion will be centered around five principles of ending hunger and poverty namely *“Change of Mindset; Good Leadership; Vision; Commitment; Action”* –

Please use the adapted VCA Manual presented in Annex 3.

3.2 Participatory evaluation

Learning objective

- Participants able to carry out an evaluation of their current situation and progress made, with respect to the prevention and control of malaria.

Materials

- Newsprint paper and markers for each small group of participants

Course content

1. Remind participants that the community and local authorities have an important role in malaria control activities, through their participation in **planning** and **interventions**. Therefore, they should also have a role in the **evaluation** of these activities.
2. Explain that the **objective** of participatory evaluation is to determine whether progress has been made in relation to malaria prevention and control.
3. The evaluation consists of **two parts**: a description of the situation **at the beginning** of the community workshops, and a description of the situation **after one year** of community workshops.
4. The **difference** between the situation at the beginning and the situation after 1 year is the **progress** that has been made in the community.
5. If the situation is not described at the beginning, it will NOT be possible to measure progress. Progress can only be measured if there is a **starting point** (baseline).

6. Participatory evaluation is an evaluation conducted by the community themselves. By being participatory, the activity will enable the community to **evaluate their own achievements**.
7. Participatory evaluation will **strengthen local ownership** over the malaria prevention programme, and will contribute to the empowerment and self-mobilization of the community. This will help the community to continue malaria prevention activities even after the project has come to an end.
8. To conduct the actual evaluation, <u>take the following steps</u>:
9. Divide the participants into several smaller **groups**, each with a good representation of the community, and ask each group to select its chairperson and a secretary to prepare the newsprint presentation.
10. Give each group the task to **describe the current situation**. Ask one person in each group to take notes. Six components should be considered:
    A. <u>Current situation on **malaria**</u>: Discuss how malaria is currently affecting life in our village/community, especially for pregnant women and under-5-year-olds.
    B. <u>Current situation on our **knowledge**</u>: How is our current understanding about malaria; do we understand what it is, how it is caused, and how it can be controlled?
    C. <u>Current situation of **health services**</u>: Describe how prompt and effective diagnosis and treatment is currently being provided at the health facility.
    D. <u>Current situation on **bed nets**</u>: Describe whether we currently have adequate and good-quality bed nets, or whether there are shortcomings.
    E. <u>Current **vision** about the future</u>: To what extent do we think that we are able to change or influence the malaria problem in our village? (for example, do we think that the situation will remain the way it always was, or do we think that we can achieve improvements?)
    F. <u>Current situation on **collaborative action**</u>: Describe how villagers, and others (village head, committee members) are currently collaborating in the fight against malaria (for example, do people work together to raise awareness or to reduce risk of malaria transmission, or is this lacking?)
11. The participatory evaluation is conducted once in the beginning of the training, and once again at the end of the training. Therefore, the **notes** taken at the beginning **should be kept** until the end of the year, so that a comparison between the two evaluations can be made.

Group discussion

1. Why is the participatory evaluation important in our community?
2. <u>Only to be answered for the evaluation at the end</u>: For each of the six components (A-F), ask the groups to compare the situation at the end with the situation at the beginning. Refer to the notes taken at the beginning and at the end. Identify the change (worsening, improvement) that has taken place.

4. MALARIA BASICS

4.1 Malaria transmission cycle

Learning objective

- Participants able to describe how the malaria parasite is transmitted between humans and mosquitoes.

Materials

- Visual material showing the transmission cycle (Visual 1)

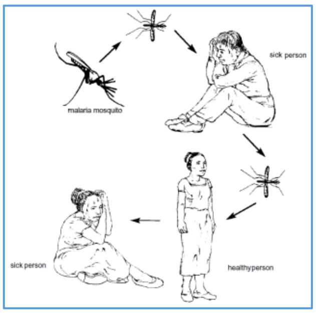

Visual 1. Malaria transmission cycle (source: WHO)

Course content

1. Ask participants: **What is malaria**?
   - Malaria is a disease caused by very small parasites present in the blood. The malaria parasites are so small that they can only be seen through a microscope.
   - The malaria parasite makes you ill by destroying part of your blood cells.
2. Ask: **how do you catch malaria**?
   - You catch malaria when bitten by an infected mosquito.
3. Ask: Where does the **mosquito get infected**? Is it born with the infection?
   - The mosquito gets infected when it bites a person infected with malaria because the mosquito sucks the blood which contains parasites. The mosquito is not born with the infection.
4. Ask: how can the malaria **parasite spread** within the community?
   - When a mosquito picks up the malaria parasite and later on bites a healthy person, it passes the malaria parasite to that person. That person also becomes ill with malaria.
   - When this sick person with malaria parasites in the blood is bitten by another mosquito, that mosquito sucks up the blood with parasites and also gets infected.
   - This way, the cycle of transmission goes on and on (unless we stop it).
5. Show and explain the visual material with the transmission cycle.

Group discussion

- What are common beliefs in the community about how we get malaria?
- Where mosquitoes are absent, can malaria still circulate in the community?
- At which time of the year is malaria most common? Why would that be?
- Discuss how we could break the cycle of transmission of malaria parasites from mosquito to human to mosquito.

4.2 Malaria signs and symptoms

Learning objective

- Participants able to explain the signs and symptoms of malaria and the burden caused by malaria

Materials

- None

Course content

1. Ask participants to recall from the previous session: **What is malaria** and **how do we get malaria**?
    a. Malaria is a disease caused by very small parasites present in the blood. The malaria parasite makes you ill by destroying part of your blood cells.
    b. A person becomes infected with malaria parasites through the bite of a mosquito that carries the parasite.
2. Ask: **what are the signs and symptoms** of malaria?
    a. The most important sign of malaria is **fever**. After the initial fever, the fever will recur at regular intervals, with attacks every 2-3 days.
    b. Each attack starts with **shivering** and **body shaking**, some hours of fever, followed by excessive **sweating**
    c. **During an attack**, the person can have headache, backache, aching all over the body, weakness. The person loses appetite; there may be vomiting and diarrhea.
    d. **If untreated**, the malaria attacks can last weeks or months, and cause poor health and weakness
    e. **Repeated infection** can lead to anemia (a condition in which a person's blood is not healthy as it should be) and exhaustion
3. Ask: Is malaria **dangerous**?
    a. Yes! In some cases, a person can become very ill very quickly, lose consciousness, and at times start twitching and shaking. This is called 'severe malaria'
    b. When not timely treated, severe malaria can develop in brain malaria, a very dangerous condition
    c. Severe malaria can cause seizures and failure of organs
    d. Severe malaria can cause **death**
4. What are effects of malaria in **pregnant women**?
    a. Maternal anaemia leading which can lead to maternal deaths or requiring blood transfusion;
    b. Low birth weight deliveries of babies;

c. Fetal (unborn child) death and perinatal loss (baby dies around the time of delivery);

d. Premature delivery

5. Effects of malaria in **children**

a. Anaemia

b. School absenteeism

c. Decreased school performance

d. Enlarged spleen

e. Death

f. Severe malaria.

Group discussion

- What are other common illnesses with symptoms similar to malaria?
- How does malaria affect the health status of the household?
- How does malaria affect our daily lives? Think of:
    - Physical growth and development
    - Performance of school children
    - work performance
    - our expenses
    - the economic development of the community or village

4.3 Malaria diagnosis

Learning objective

- Participants able to explain the process and importance of early diagnosis and treatment.

Materials

- Malaria Rapid Diagnostic Test (RDT) kit
- Pricker, glass slides
- Visual on RDT showing test lines (Visual 2)

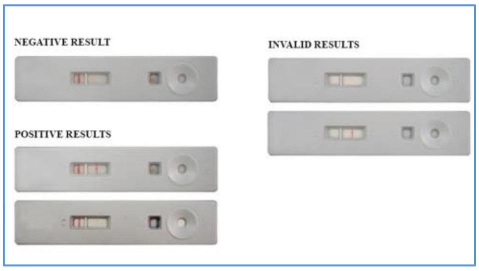

Visual 2. Malaria Rapid Diagnostic Test kit, with results (source: WHO)

Course content

1. It is often difficult to recognize malaria in a sick person. There are several diseases that have signs and symptoms **similar to those of malaria**. If a person has a fever, it could be malaria, but it could also be something else.
2. Why is diagnosis important?
    - Because malaria treatment must only be given to patients with **confirmed** malaria
    - If it is not malaria, another **appropriate treatment** must be sought
    - Giving malaria treatment to someone who does not have malaria is a **waste** of resources; also, there could be some side-effects of the medication
3. Ask: How is malaria diagnosed?
    - Diagnosis is by the health care worker.
    - The signs and symptoms in the patient can suspect that the patient has malaria. Measuring of body temperature can confirm a fever. These signs and symptoms do **not confirm** that the person really has malaria.
    - All suspected malaria cases must be confirmed through a **diagnostic test of a blood** droplet by a qualified person.
    - When health care worker suspects malaria, they can test the blood of the patient to confirm.
4. How is the test done?
    - The test is by **RDT-kit** (RDT = Rapid Diagnostic Test), or sometimes through a microscope (explain that a microscope is a glass lens through which the malaria parasites can be seen in a droplet of blood). Show microscope slide.
5. Show the RDT kit with the visual, and explain the following points:
    - The RDT should show the **control line** whenever it is tested with a droplet of blood. Without the control line, the test is not valid.

    - If the RDT shows a control line but **no other line**, the person does not have malaria. It is a 'negative' result.
    - If the RDT shows the control line, but also a second line is visible, called the **test line**, it is confirmed that the person has malaria. This test line can be faint. This test line is caused by the malaria parasites present in the blood droplet.
6. The HSA (or other qualified person) reads the result of the RDT test and interprets the result: the patient has no malaria or has confirmed malaria.
7. Only when malaria is confirmed, medication should be given.

Group discussion

- What are other causes of fever in a child (other than malaria)?
- Discuss why suspected malaria cases should seek diagnosis promptly, without delay.
- What are barriers people encounter in seeking proper diagnosis? How could these barriers be solved?
- What are people's experiences with receiving diagnosis at a health facility? How should the Majete Malaria Project help improve the situation?

4.4 Malaria treatment and compliance

Learning objective

- Participants aware that malaria can be treated, and able to explain the importance of early treatment and completion of the treatment course according to prescribed schedule.

Materials

- Example of LA medication (blister)

Course content

1. Can malaria be treated? Yes! **Effective treatment** of malaria is available.
2. When malaria is suspected, a person should **seek medical care** immediately. If the diagnostic test is positive for malaria, appropriate medication should be administered
3. Treatment is important for two reasons:
    a. Treatment **cures** the infection in the patient and prevents that the disease progresses to severe malaria
    b. Treatment eliminates the parasites, which helps **prevent** that other family members become infected
4. Uncomplicated malaria: When the patient has ‘uncomplicated’ malaria (this is when malaria is not severe), **LA** (lumefantrine-artemether) will be administered for 3 days: the first, second and third day of the treatment course. It is best to take this medication with or immediately after a meal.
5. It is essential that the patient complies with the **full course of treatment**, and does not stop after the first or second day. Even when disease symptoms disappear after the first day, the treatment should be taken for the full three days.
6. To reduce suffering during fever, paracetamol or ibuprofen may be administered to the patient.
7. Occasionally, malaria treatment can fail. This is when the medicine is vomited or when the full course is not completed. When a treatment has failed, malaria should then be treated with a second-line anti-malarial drug (not LA).
8. If the malaria fever comes back after 14 or more days after the treatment, this is considered to be a new infection, and LA should be administered.
9. Severe malaria: Severe malaria is a medical emergency, requiring prompt treatment. It may lead to **death** if untreated. Severe malaria will be discussed in more detail in a separate workshop session (7.1)
10. Severe malaria requires **hospital admission** and intravenous treatment with quinine and other medicines
11. People with malaria can also die if there is delay in seeking treatment. It is therefore important to seek medical care once we feel unwell or notice that our child is ill.

Group discussion

- Are there traditional medicines available in our community? If yes, are they effective?
- What is your experience with medical care on malaria through the health system?
- What are possible reasons why some people do not seek care from the health facility, even though they suspect they have malaria?

4.5 Malaria prevention

Learning objective

- Participants able to outline the available strategies for malaria prevention

Materials

- Visuals on house screening and bed nets (Visual 3)

Visual 3. House screening and bed net (source: WHO)

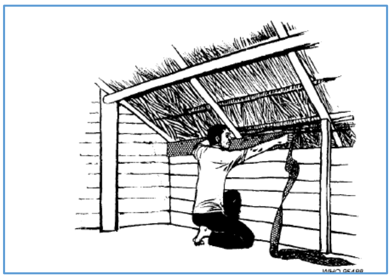

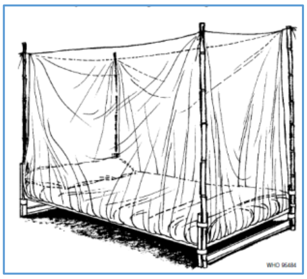

Course content

1. Can malaria be prevented? Yes!
2. Remind the participants of the malaria transmission cycle, and ask participants at which stages of the transmission can be stopped.
    a. Prevent mosquitos from **biting** people
    b. Control mosquito **breeding**
    c. Kill **adult mosquitoes**
    d. Seek early **treatment**
    e. Seek **preventive** treatment
3. How can we prevent mosquitos from **biting** people?
    a. Sleep under mosquito nets (ordinary or insecticide treated)
    b. Screen all windows and doors in the house. Or at least in rooms where people sleep
    c. Apply mosquito repellents to the skin
    d. Burn mosquito coils
4. How can we control mosquito **breeding**?
    a. Eliminate water bodies where mosquitos breed
    b. Reclaim land by filling and draining
    c. In water bodies that cannot be eliminated or drained, for example, swamp areas, put special insecticides in the water to kill mosquito larvae.
5. How can we **kill** adult mosquitoes?
    a. Spray rooms with household insecticides before going to bed

   b. Participate in activities carried out by the health services, such as spraying the inside walls of houses with residual insecticides that kill mosquitoes that rest on the walls.

6. How can **early treatment** prevent malaria?
   a. Early treatment helps reduce amount of malaria causing parasites from the human population which can be transmitted by mosquitoes.
   b. Early treatment in one person can prevent malaria of a neighboring person

7. How can we prevent malaria in **pregnancy**?
   a. Taking intermittent preventive therapy (SP)
   b. Sleeping under a mosquito net

Group discussion

- Can malaria be controlled?
- Can malaria be eliminated?

4.6 Vulnerable groups

Learning objective

- Participants able to describe the vulnerability of pregnant women and young children, and the need for IPTp for pregnant women, and early diagnosis and treatment for young children

Materials

- Sample of fansidar (SP)

Course content

1. Ask: who is **at risk** of getting malaria?
    a. Everyone is at risk of contracting malaria.
    b. People in most areas of Africa are at risk of malaria infection. In Malawi, people living within the Lower Shire river valley and along the lake shore are especially at high risk of malaria infection (e.g. Chikwawa, Nsanje, Salima, Nkhotakota, Mangochi).
2. Ask participants: Which groups of people are **more vulnerable** to malaria than others living in the same village?
    a. **Young children** (especially those under 5 years) are most at risk of developing severe malaria; this is the dangerous form of malaria.
    b. **Pregnant women**, especially those that are pregnant for the first time, are also at high risk of developing severe malaria. Malaria in the pregnant woman can affect the unborn child.
    c. **Visitors** from areas without malaria (for example, highland areas) are also at risk of developing severe malaria, because they are not used to being exposed to the malaria parasite
3. Ask: Why do these groups **suffer most** from a malaria attack?
    a. Young children have had no **previous attacks** of malaria (or olny few attackes). Therefore, their young bodies are not yet used to malaria. Young children with malaria can develop anaemia (reduced red blood cells), an enlarged spleen, and severe malaria. This can lead to death.
    b. Similarly, visitors from malaria-free areas have not recently been exposed to malaria. Their bodies are not used to malaria.
    c. On the other hand, persons who have had malaria several times in their lives, have milder attacks of malaria. Some persons only get a slight raise in temperature, but hardly notice the ‘attack’. Their previous malaria attacks give them some level of **immunity** against malaria. This **immunity is lacking in young children**.
4. Ask: And why are **pregnant women** so vulnerable to malaria?
    a. When pregnant, a woman loses this immunity to malaria and **becomes vulnerable** to having some specific complications of malaria, with risks to the unborn child.

b. A pregnant woman with malaria can get **anaemia** which can lead to maternal death.

c. Malaria can also cause premature delivery, low birth weight, or death of the **unborn child**.

5. Ask: What should be done to **protect young children**?

    a. Young children should be carefully protected from mosquito bites at night by **sleeping under a net**.

    b. If malaria is suspected, young children should be immediately brought to a health facility for **diagnosis and treatment**.

6. Ask: What should be done to **protect women during pregnancy**?

    a. Pregnant women should always sleep under a net at night to be protected from mosquito bites.

    b. Pregnant women should receive a so-called "intermittent presumptive therapy during pregnancy" (IPTp) with the antimalarial drug **fansidar** (SP). This protects the pregnant woman against malaria, and protects the unborn child.

    c. The first dose of fansidar should be at the time that the first fetal movements are felt by the mother. From that time onwards, the treatment should be **repeated** at each scheduled antenatal care (ANC) visit to the health facility. Each SP dose should be at least 1 month apart, and SP doses should be continued up to delivery.

Group discussion

- What problems are encountered in protecting young children from malaria, or getting young children the treatment for malaria?
- What problems or barriers do pregnant women experience with receiving IPTp treatment at regular intervals?

5. VECTORS AND THE ENVIRONMENT

5.1 Biting behaviour of the malaria mosquito

Learning objective

- Participants able to describe the differences in biting behaviour of nuisance mosquitoes and malaria mosquitoes and identify measures how they can prevent malaria mosquito bites.

Materials

- Visual of malaria mosquito and nuisance mosquito (Visual 4)
- Sample of blood-fed female malaria mosquito inside plastic tube of bottle

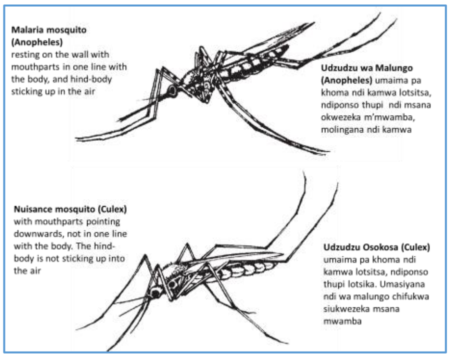

Visual 4. Malaria mosquito and nuisance mosquito (source: WHO)

Course content

1. Mosquitoes search for humans when they are hungry for a blood meal. They use their **sense of smell** to find humans
2. Nuisance mosquitoes (*Culex*) can bite early evening and at night
3. Malaria mosquitoes (*Anopheles*) bite late **evening** and throughout the **night**
4. Bites of nuisance mosquitoes are easily noticed but not dangerous
5. Bites of malaria mosquitoes are **not easily noticed** but are dangerous
6. Most nuisance mosquitoes bite **animals** as well as humans
7. Most malaria mosquitoes prefer to feed on **humans**, not animals

   In summary:

**Nuisance mosquitoes**	**Malaria mosquitoes**
Bite early evening and at night	Bite late evening and at night
Bite is easily noticed, but not dangerous	Bite is not easily noticed, but dangerous
Bite animals and humans	Bite mostly humans

8. Only **female** mosquitoes bite and feed on blood; the males do not bite
9. After a blood meal, the female **rests on the wall** for two days while the blood is digested and eggs are developed.
10. After the **eggs** are laid, the female will again search for a blood meal.

11. Show sample of **blood-fed mosquito** (live specimen in plastic tube, or picture); ask if participants have noticed blood-fed mosquitoes around their bed after they wake up in the morning.
12. Not all malaria mosquitoes carry the malaria parasite – only some do. We cannot distinguish which ones are infected and which ones are normal.
13. Ask participants how we can **avoid being bitten** in the evening or at night. Only correct the answers when needed. Personal protection measures are: bed net use, repellents, and avoiding exposure in the evening and at night.

Group discussion

- Where are malaria mosquitoes in the day-time?
- How much of a nuisance do mosquitoes or other pests cause at night (cockroaches, spiders, biting bedbugs, rodents)?
- Do personal protection measures also protect against other pests?

5.2 Life-cycle and breeding of the malaria vector

Learning objective

- Participants able to describe the mosquito life-cycle and breeding sites.

Materials

- Plastic container with water and mosquito larvae (malaria and/or nuisance mosquitoes)
- Prior to the meeting, survey for a larval breeding site near the meeting location; this should be used for a field visit
- Visual of mosquito life-cycle (Visual 5)

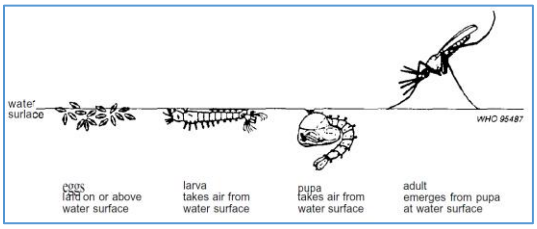

Visual 5. Life-cycle of the malaria mosquito (source: WHO)

Course content

1. Remind participants of the **role of malaria mosquitoes** and ask where these mosquitoes are coming from
2. Explain that **standing water** plays an essential role in the life of a mosquito, because that is where its development takes place before it becomes a mosquito with wings as we know it
3. Draw the **life-cycle** of the mosquito on a large sheet, starting from the tiny eggs until the emergence of the adult (use the provided diagram as example)
4. After blood-feeding, **eggs develop** inside the female mosquito (2 days)
5. Then, the female mosquito finds suitable water bodies to **deposit** its many eggs (around 100 eggs)
6. Eggs **hatch** in the water, and the emerging larvae live under water (show sample of live larvae)
7. The **larvae** feed on minute plants (algae) and bacteria on the water surface
8. When fully grown, the larva becomes a **pupa**, which is a kind of cocoon in which the adult mosquito is formed and shaped
9. Finally, the new **adult mosquito emerges** from the pupa at the water surface
10. Not all water bodies are suitable for malaria mosquito larvae. Most malaria mosquitoes like **clear water**, in the sun, or water with water plants.
11. If the water is not clear but **polluted**, it will not produce malaria mosquitoes but it can produce nuisance mosquitoes

Group discussion

- Why is it a problem when water bodies are located near people’s houses?
- Can mosquitoes survive where there is no standing water?
- When looking at the life-cycle, when are the best stages to kill the mosquitoes?

5.3 Recognizing the mosquitoes

Learning objective

- Participants able to distinguish malaria mosquito from nuisance mosquito

Materials

- Visual of malaria mosquito and nuisance mosquito (Visual 4)
- Dried or live specimens in vials

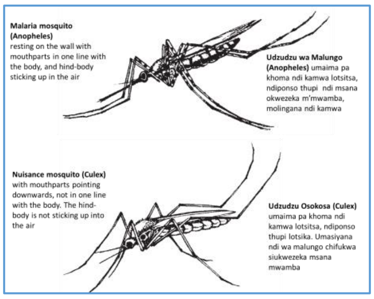

Visual 4. Malaria mosquito and nuisance mosquito (source: WHO)

Course content

1. Remind participants of the **role of mosquitoes** in the transmission of the malaria parasite: without the mosquito there cannot be malaria circulating within the community
2. Point out the difference in **resting position** between malaria mosquitoes and nuisance mosquitoes, by drawing on newsprint paper and by illustrating with a visual material. Ensure that all participants have a chance to see the visuals
3. **Malaria mosquitoes** (*Anopheles*) can be recognized when they are resting on the wall with mouthparts in one line with the body, and hind-body sticking up in the air
4. **Nuisance mosquitoes** (*Culex*) can be recognized when they are resting on the wall by having their mouthparts pointing downwards, not in one line with the body. The hind-body is not sticking up into the air.
5. Emphasize that nuisance mosquitoes do not transmit malaria (but they can transmit lymphatic filariasis; this disease is now rare because it has almost been eliminated from Chikwawa)
6. Only malaria mosquitoes are able to **transmit** malaria parasites. Therefore it is important we know the difference with nuisance mosquitoes
7. Mosquitoes can fly 1000 meters or more, but are 'lazy' because they do not fly far when their human blood host is close by and when they can breed in the vicinity
8. Female mosquitoes can live up to a month, but most die within 1 or 2 weeks
9. Male mosquitoes feed only on nectar and sugar from flowers

Group discussion

- Which mosquitoes are most common and cause most nuisance problems?
- How can we check whether malaria mosquitoes are present in our house?
- Are mosquitoes common in all houses of the village or are they more common in some parts of the village? If so, why?
- Which other household pests are common, and what problems do they cause?

5.4 Risks of contracting malaria parasites

Learning objective

- Participants able to demonstrate skills of analyzing the local risk factors of getting malaria parasites.

Materials

- Newsprint paper; markers

Course content

1. Malaria parasites can spread and **circulate** within the village only when the conditions are favorable for malaria.
2. But what are those conditions? Malaria parasites need the mosquito as the **vector** for transmission, **human** hosts and a suitable **environment**. If any of these elements are missing, malaria cannot exist.
3. Draw the three components on a large newsprint paper. Then ask participants what happens to malaria when one of these components is **missing**. For example, what happens to malaria when the vector is missing? The same for when there are no humans in the area?
4. Draw a simple **map** with a few houses of the village on a large newsprint sheet of paper. Also draw some fields and bushes. Indicate that some are traditional houses and some modern houses. Draw some water pools or a swamp at one side of the map.
5. Participants are reminded that mosquitoes breed in **water bodies**, and that adult mosquitoes move to **houses** where people live.
6. Explain that clearing bushes will <u>not</u> prevent malaria (even though many people believe it does), but clearing water bodies in which the mosquitoes breed can prevent malaria

Group discussion

- Looking at the hand-drawn map, ask a participant to show how the mosquitoes will move around
- Ask participants where on the hand-drawn map the people are most likely to get malaria, and why they think so. Indicate this on the map.
- Ask whether house type or house condition influences the risk of malaria
- What can the villagers do to reduce the bites by malaria mosquitoes?

6. PERSONAL PROTECTION FROM MALARIA

6.1 Bed net hang-up

Learning objective

- By the end of this session, the participants will demonstrate skills in the proper hang-up of a bed net.

Materials and preparation

- Hammer, nail, hooks, strings, bed net
- Try-out the hanging of the bed net in the demonstration site prior to the workshop
- This workshop should be planned to be held approx. 2 weeks before the timing of distribution of bed nets.

Course content

1. Remind participants of the **role of malaria mosquitoes** in transmitting the malaria parasite. These mosquitoes bite at night, so we can protect ourselves from their dangerous bites by sleeping under a net.
2. The bed net can be **effective** in protecting against malaria, but only if the nets are correctly hung and properly used.
3. Ask participants about their **experiences** using nets: Are children protected? Are people getting used to sleeping under nets? Do nets protect against household pests and nuisance insects?
4. Demonstrate to the group **how the net should be hung**. Ask several persons to assist in the hang-up. Start from unwrapping the net. Use an example bed net provided by the malaria programme.
5. Discuss the following points:
    a. When hanging the nets, the net should have a **fixed position**. This will make it easier to use the net every night.
    b. Identify the sleeping positions in the household and **plan the positions** of each net. One nets can accommodate two persons, or an adult person with two children.
    c. For each position where a net will be hung, check whether it is possible to **fix the four strings**. The net is provided with a string on each upper corner. Determine the length of the strings needed, and tie longer strings as required.
    d. Fix strong **hooks or nails** so that each string is tied to the wall, ceiling or other structure in the room. Make sure that the string can be untied to allow for the net to be washed occasionally.
    e. Hang the net so that the bottom part **rests on the floor**, or can be tucked underneath the mattress or mat.

f. Most common problems with nets are the holes created by **sharp objects**, such as the edges of bamboo mats or wood. Inspect the edges of mats or beds and do away with sharp edges, for instance by covering with cloth.

g. Demonstrate how the hanging net is **folded** (while keeping it in its hanging position) during the daytime so as to avoid damage.

6. Note that the nets are not made from ordinary nylon, but contain a **special substance** (insecticide) against mosquitoes. This can repel or even kill the mosquitoes landing on the net.

7. Clarify that this substance against mosquitoes can remain effective for a period of three years. However, if the net is washed too often, the substance is quickly removed from the net. It is important that the net is **not washed more than once every two or three months** (once every 8-10 weeks).

8. Also important is that after washing the net, the net should not be dried in bright sunlight, because this will also remove the substance against mosquitoes. Washed nets should only be **dried in the shade** (for example under a tree). Sunlight is harmful to the net.

Group discussion

- What problems do people encounter in hanging the nets, and how can these problems be solved?
- For what reasons do people not want to hang or use nets? Are these valid reasons, and if not, why?
- Inquire which households need assistance with net hang-up. Ask if neighbors are able to help. If not, note down their name and address, and make follow-up arrangements to visit their homes.

6.2 Bed net distribution

Learning objective

- Participants able to describe the purpose and mechanism of bed net distribution, which is to achieve universal coverage of 1 net per 1 or 2 persons.

Materials and preparation

- This workshop will prepare the villagers for the distribution of bed nets. The **timing** of this VCA session has to be a few days before the actual planned distribution. Distribution will be through the health system and with active participation of the health animators and village heads. The timing depends on the availability of the stock of nets in the village.
- This workshop session will help publicize the net distribution, aiming to reach all households.
- Stock of bed nets should be available and ready for distribution in the village cluster, with strings and hooks, as applicable.

Course content

1. Announce the due **dates for net distribution** in the village.
2. The participants are reminded of the vital **role of bed nets** in protecting against malaria infection. The nets are a barrier between mosquitoes and humans. The mosquito can transfer the malaria parasite to a person through its bite.
3. Participants are reminded of the methods of **net hang-up**: using fixed positions of nets, placing hooks, adjusting the correct height of the net, removing sharp edges of mats or beds.
4. Explain the **planned distribution** of bed nets, which will be from house-to-house. All houses will receive bed nets. The number of nets will depend on the number of people in each household. In general, one net will be issued for every two persons in the household.
5. Nets will only be given upon **signatory** by a household member. Discuss the conditions of the signatory in detail.
6. Explain to the participants that by signing for the net, they **commit** to daily use, correct use, maintenance and repair of the nets. They also commit to giving children priority over adults to sleep under the nets. Nets should not be sold, used for unintended purposes or be taken away from the household.
7. By signing, they enter an **agreement** with the malaria programme that they will allow monitoring teams access to their houses to inspect net use, provide advice and assistance on use and maintenance.
8. If time allows, the hang-up demonstration of the previous session is repeated for newcomers.

Group discussion

- Ask participants whether the conditions of signatory pose any problems (for example, is inspection by monitoring teams acceptable)?
- Ask participants whether they are ready for hang-up and use of the bed nets, or whether assistance with hang-up is required.

6.3 Bed net use

Learning objective

- Participants able to demonstrate skills in correct use of the bed net to provide maximum protection against the bites of malaria mosquitoes.

Materials

- An example bed net should be hung-up over a bed, for demonstration purposes.

Course content

1. Remind the participants of the **role of bed nets** in providing a barrier between malaria mosquitoes and humans. The mosquitoes bite in the evening and at night and therefore, sleeping under a net will protect against malaria infection. The bed net can be very effective in protecting against malaria, but only if the nets are used properly.
2. If there are insufficient nets to cover all household members, **children should have priority** over the of nets because they are most vulnerable to malaria infection
3. For proper bed net use, three components must be considered: (1) How the nets are **prepared for sleep**, (2) how the nets are **used during sleep**, and (3) how the nets are **kept after sleep**.
    a. Nets should be hung in such a way that it makes daily use easy and hassle-free. Having a bed hung in a **fixed position** with the sides flapped over the top, makes it easy for the sleeper to correctly use the net after dark. Preparations could also be made to avoid that the sleepers touch the net during sleep. This could for example be achieved by placing clothes at the inside of the net where arms or feet are likely to touch the net.
    b. During sleep, the edges of the net should be **touching the ground** or be tucked under the matrass or mat to prevent mosquitoes from entering the net. Also, sleepers should not touch the net because the mosquito could bite the person through the net. Parents or siblings should check whether young children are sleeping correctly under the net.
    c. In the morning, the nets should be kept in fixed position. Even though removing and storing of the net during the daytime will keep the net in a better condition, it is still considered best to leave the net in its fixed position because this will make it much easier to use the net each night. The sides of the net are **flapped on top**, but care must be taken that sharp edges of mats or beds do not tear the net. Measures should be taken to **remove sharp edges**.
4. Ask the participants which time of the year they should use the nets. After their responses, explain that malaria mosquitoes can bite in **any night during the year**. Also when it is dry season and when nuisance mosquitoes are not a

problem, a malaria mosquito can still transmit the parasite. It may seem that there are no mosquitoes around, but malaria mosquitoes are not easily noticed.

Group discussion

- What are people's experiences with sleeping under a bed net? What advantages and disadvantages do they experience?
- Do people sleeping under a net still get bitten by mosquitoes? What is the cause (touching the net, holes, improper hanging of net)? How can this be solved?
- If household members do not use bed nets, how could they become familiarized with sleeping under a net?

6.4 Bed net maintenance and repair

Learning objective

- The participants demonstrate skills in proper maintenance and repair of bed nets so that the net can provide good protection for a period of 3 years.

Materials

- Example of a torn bed net, with several holes
- Example of a repaired bed net, with several mended holes
- This session should be selected several months after the distribution of bed nets has taken place. This session can be conducted more than once.

Course content

1. Start by reminding participants of the **benefits of bed nets**, which is to prevent transmission and circulation of malaria parasites from malaria mosquitoes to humans.
2. Explain that the fabric from which the nets are made is strong and can withstand daily use, provided that the nets are used with care. **Sharp objects**, such as the edges of bamboo mats or nails, easily tear holes in the nets. Mosquitoes could enter the nets through these holes.
3. The nets are not made from ordinary nylon, but contain a **special substance** (insecticide) against mosquitoes. This can repel or even kill the mosquitoes landing on the net.
4. Clarify that this substance against mosquitoes can remain effective for a period of three years. However, if the net is **washed too often**, the substance is quickly removed from the net. It is important that the net is not washed more than once every two or three months (once every 8-10 weeks).
5. Also important is that after washing the net, the net should <u>not</u> be dried in bright sunlight, because this will also remove the substance against mosquitoes. Washed nets should only be **dried in the shade** (for example under a tree). Sunlight is harmful to the net.
6. If there are not sufficient nets to cover all household members, **children should have priority** over the of nets because they are most vulnerable to malaria infection
7. Removing and storing the net during the daytime will keep the net in a better position. Nevertheless, it is still considered best to leave the net in its fixed position because this will make it much easier to use the net the next night.
8. Ask participants to examine their own bed nets, the old ones and the newly-distributed ones, and count the number of **small holes** (finger size) and **large holes** (fist size) in each net. They should remember or record the number of holes and report them in the next VCA workshop.
9. Explain to the participants that the health animators will also conduct **monitoring of bed net use and maintenance** by visiting a selection of

houses, but not all houses. The purpose of monitoring is to make decisions on necessary actions.

10. Ask participants what could be done by the community to **repair** the nets with holes. Should repair be done within households, or could the community help each other to organize repair-workshops? What would be the role of the health animator, the village head and the HSA?

11. If **repair-workshops** are seen as an option, ask participants how this could be organized, which roles and tasks would be required, and who would take an active part.

12. Explain that community-level action is essential for combating malaria, because the disease circulates within the community, not just within households.

Group discussion

- Ask who have managed to keep their bed nets in good conditions after daily use for a year or more? What is their secret to good maintenance of nets?
- Is frequent washing of the net necessary? How can we avoid that the net becomes dirty in the first place?
- What could you do when you observe that some persons in your community or wash their nets very frequently or dry their nets in the sun?
- Are children given priority over the use of bed nets? If not, what causes this problem, and how can this be solved?

7. HEALTH CARE

7.1 Case management and severe malaria

Learning objective

- Participants able to recognize severe malaria, and able to explain the need for intensive case management at the hospital.

Materials

- None

Course content

1. Most malaria cases are 'uncomplicated malaria'. These persons will cure after medication, and usually do not need to be admitted to hospital.
2. Some malaria cases are 'severe malaria'. Persons with severe malaria need urgent medical care and hospital admission. If left untreated, severe malaria may lead to **death**.
3. Severe malaria requires **hospital admission** and intravenous treatment with quinine and other medicines
4. What is **severe malaria**?
    a. The illness starts with fever and often vomiting. Children can **deteriorate** rapidly over 1-2 days, going into coma (brain malaria), shock, convulsions, or severe anemia (reduced red blood cells).
5. Who is **most at risk** of severe malaria?
    a. Young children and pregnant women are the groups at highest risk of severe malaria. This is because they lack sufficient immunity against malaria.
6. What are the **features** of severe malaria?
    a. General weakness (child can no longer walk or sit up without assistance)
    b. Breathing problems
    c. Yellowing of eyes
    d. Profuse vomiting and unable to take feeds
    e. Loss of consciousness, convulsions
    f. Shock
    g. Heavy diarrhea
    h. Anaemia (reduced red blood cells)
    i. Low blood sugar
7. What could be the **complications** caused by severe malaria?

a. Brain infection

b. Destruction of blood cells

c. Organ failure such as kidney or liver

d. Meningitis

e. Fluid in the lungs

f. Massive internal bleeding

g. Low birth weight deliveries of babies.

h. Maternal anaemia leading to maternal deaths

i. Effects to the unborn child (low birth weight; loss of the fetus)

8. How is severe malaria treated?

   a. When a patient with ‘severe malaria’ is presented to the community clinic at the local level, the patient should receive special antimalarial **treatment before being referred** to a hospital. **Prompt referral** is especially important in patients with serious complications or patients requiring a blood transfusion.

   b. In the hospital, the severely ill patient will need very **intensive nursing** care, especially when the patient is unconscious.

   c. The patient may have several **complications** that are life-threatening. Special care should be taken to ensure that the airways are free and that breathing is normally. The patient will need intravenous infusion of medicines and fluids. The patient should be monitored for low blood sugar and managed accordingly.

   d. Specific **antimalarial medicines** are recommended for cases of severe malaria. Fever should be reduced with paracetamol.

   e. If the patient has severe anaemia (lack of red blood cells), a **blood transfusion** may be necessary to safe life.

Group discussion

- Have cases of severe malaria occurred recently in your community? If so, have they received proper medical care and nursing care?
- Are patients with severe malaria always presented to the health facility promptly, without delay. If not, what are the causes of the delay, and how could the delay be prevented in future?
- Discuss problems encountered in treatment of patients with severe malaria, both at the local community clinic and at the hospital. What solutions could be proposed?

7.2 Community and health system

Learning objective

- Participants able to explain the structure of the health system and its linkage with the community

Materials

- Newsprint paper; markers

Course content

1. By 'health system' we mean: the **personnel, institutions and resources that deliver health services to the people**. The major aim of the health system is to deliver quality health services to the population.
2. The health system in Chikwawa district is headed by the **District Health Office.** Under the district health office come all the health facilities and health staff. These include: community clinics, health centres (public and private), and the district hospital.
    a. At the local level, **community clinics** provide some basic health services and refer patients to the health centres when required. Community clinics are run by the HSAs. In their villages, the HSAs provide basic medical care for under 5-year-olds and implement immunization. HSAs also follow up patients discharged on TB treatment and postnatal mothers. They do HIV counseling and supply health information.
    b. **Health centres** provide primary health services, and often provide overnight stays. If patients require more specialized (secondary) services, they are referred to the district hospital. Health centres include public health centres and private facilities, such as those of the Christian Association of Malawi (CHAM). Private facilities are supervised by the District Health Office.
    c. The **district hospital** provides secondary health services for the district available for free. It provides specialized treatment for overnight stays.
    d. If patients require specialist services not available at the district hospital, they are referred to the **central hospital** in Blantyre.
3. The health system operates a number of **health programmes**, such as: childhood illness, immunization, reproductive health, safe motherhood, child and adolescent health. The National Malaria Control Programme is also implemented through the health system.
4. At local level, the **HSAs are the implementers** of these health programmes, which include malaria control [i].

[i] In 2015, the HSAs, or community health workers, have been trained, and now implement the malaria testing using RDTs, and prescribe LA for uncomplicated malaria in children below five years old at the community level. Children with severe illness are referred to the health centre.

5. Sometimes, health programmes can be combined. For example, antimalarial treatment of pregnant women (IPTp) can be scheduled during antenatal care (ANC) visits. This helps the women getting most out of their health care visits.
6. The **health committee** is a group of people that represents the community, and that has a role in advising the health centre in the area. Its members may include traditional and religious leaders, women and political representation. The health committee is attached to a specific health centre.
7. The health committee should **function in two ways**: by voicing concerns and demands of the community to the health facilities and health centre, and by informing the community about health programmes.
8. The **health animator** is established by the Majete Malaria Project. The health animator lives in the community and connects the community to the health system. The health animator educates the community through workshops to adopt healthy practices and behaviours related to malaria control, and to actively seek and comply with malaria control services. Health animators meet regularly with the HSAs.
9. Without **supplies**, the health system cannot function. Under the Majete Malaria Project, all supplies necessary for malaria control should be available to all households in the project area. These malaria control supplies include: diagnostic kits, antimalarial treatment, and bed nets.
10. In case there is a shortage of these supplies or services to people in need, the community should **voice their complaints** via the health animators to the health committee members.

Group discussion

- What is your experience with medical care on malaria through the health system? What problems are encountered, and what are possible solutions?
- What steps could the community take when appropriate health care is not provided by the health system (for example, when medication is not available)?
- Is there a role for traditional medicine in malaria control?
- What do you notice about the health committee or its members in your area? Do you know to whom you can voice your concerns or complaints about health services?

7.3 Mother and infant care

Learning objective

- Participants able to explain the role of the mother in management of malaria and to recognize important signs and symptoms in newborns and infants which indicate severe illness.

Materials

- None

Course content

- Ask: What is the role of mothers in **malaria management** in pregnancy and for infants?
    - Antenatal care as early as possible and take fansidar (SP) to decrease the chances of malaria
    - Use of mosquito net during pregnancy since pregnant women can easily contact malaria
    - Ensure that the infant is protected from insect bites by making sure it sleeps under a mosquito net
    - Recognize when the child is unwell
    - Promptly seek help for ill infants
- Routine care of the newborn **at delivery**:
    - Dry the baby with a clean towel, and observe the baby when drying
    - Give the baby to the mother as soon as possible, place on her chest or belly
    - Cover the baby to prevent heat loss
    - Encourage initiation of breastfeeding within the first hour
- Routine care **after delivery**:
    - Keep dry in a warm room away from drafts, well covered
    - Always keep the baby with the mother, rooming in
    - Initiate breastfeeding within the first hour
    - Let the baby breastfeed on demand if able to suck (whenever the baby wants)
    - Keep umbilical cord clean and dry
    - The newborn should receive an oral polio and BCG vaccine
- How to **prevent early infections** in newborns
    - Practice good hygiene and cleanliness during delivery
    - Strict procedures for hand washing for all staff and for families before and after handling babies
    - Special attention to care of the umbilical cord
    - Always practice exclusive breastfeeding (no supplement food or drink)
    - Eye care

- **Danger signs** in newborns and young infants: Neonates and young infants often present with signs which indicate severe illness. Ask mothers if they have seen any of these signs:
    - Unable to breastfeed
    - Convulsions
    - Drowsy or unconscious
    - Cessation of breathing
    - Fast and difficult breathing
    - Grunting
    - Severe chest in drawings
    - Central cyanosis
    - Jaundice
    - Abdominal distension
    - Localizing signs like; painful joints, joint swelling, reduced movement and irritability if these parts are handled
    - Many or severe skin rashes (pustules)
    - Umbilical redness extending to the peri-umbilical skin or umbilicus draining pus.
    - Bulging fontanel
- With any of these signs, the infant should be presented to should be presented to the **hospital**.

Group discussion

- How should mothers/guardians **decide** whether they should take the sick child for help to a medical facility or traditional healers? Consider the following factors:
    - Nature of the illness: Certain ailments are attended at traditional healers while others need a medical care
    - Convenience
    - Religion and other beliefs
    - Cost
    - Relatives (spouse, parents) and friends
- What are the common problems in infant care practices in the community? How could these problems be solved?
- Have any of the danger signs been experienced? If so, was prompt and appropriate treatment received?

8. COMMUNITY ACTION

8.1 Community-wide malaria control

Learning objective

- After this session, the participants will be able to explain the need for community-wide action on malaria and identify potential players in organizing community action.

Materials

- Large newsprint paper; colour markers

Course content

1. Participants are reminded **how malaria spreads** in the village: malaria parasites are transmitted by mosquitoes from person to person; mosquitoes breed in water bodies; and adult mosquitoes move to houses where people live.
2. On a large newsprint sheet of paper, draw a simple **map of the village** with several clearly-indicated houses. Draw some water pools or a swamp at one side of the map, as an example.
3. Draw a circle around one of the houses, and ask participants: "What if this household tries to control malaria with all their means, but their **neighbouring households** don't; what happens then?".
4. Why is it important to **involve the poorest** and most disadvantaged households of the village?
5. Malaria control depends on our own actions, but also on the actions of our neighbours (they may circulate the parasite, or they may contribute to mosquito breeding).
6. Ask one of the participants to describe the **vision on malaria**, as developed during the first community workshop.
7. Then pose the question to the participants how malaria can be **prevented** in the entire village? What would be needed in terms of leadership and organization?

Group discussion

- What are the main constraints to collaboration and joint action of all villagers on malaria control?
- How can these constraints be solved?

8.2 Problem analysis

Learning objective

- By the end of this session the participants will be able to mention a variety of problems in a field setting, distinguish between cause and effect, and construct a problem tree.

Materials

- Large newsprint paper; colour markers

Course content

1. Start by explaining that there are many **types of problems** villagers face in their daily lives. Some problem are related to other problems, or caused by other problems. Explain that it is important to understand how the problems are related to each other.
2. Ask the participants what is meant by “a problem”, so that everyone has the same perception about what is required in this exercise.
3. Then, ask the participants to **identify main problems** commonly faced in the village (for example, problems related to disease, medical cost, low income, isolated location, lack of collaboration, lack of leadership, lack of services, etc.). If malaria is not mentioned as problem, ask whether it is a problem.
4. The health animator assisted by the rapporteur should **keep records** so that each mentioned problem is noted down.
5. Ensure participation of all attendants in the deliberations.
6. Next, the health animator draws the problems one-by-one on a large newsprint paper, so that everyone can see them. Put **malaria in the centre** of the paper. Draw each identified problem around the malaria problem. Use **symbols** rather than text to describe each problem (e.g. a mosquito as symbol of malaria). Clearly encircle each problem.
7. Draw a **connecting line** between the malaria problem and each other problem.
8. Then ask the participants for each of the other problems, how this problem influences malaria – **what is the relationship**? Make the line between the problems into an arrow, to indicate which is the **cause** and which is the **effect** (sometimes the arrow will go both ways).
9. Ask if there are any additional problems that need to be included for the full picture.

Group discussion

1. Looking at the identified problems, how can the malaria situation be improved?
2. What is the role of the community and village leaders in solving each problem?
3. What is the role of the government?

8.3 Community action planning

Learning objective

- After this session, the participants will be able to develop a plan of collective actions to control malaria.

Materials

- In preparation for this session, the health animator should ask the village head whether he/she can assist in facilitating the planning process.
- Large newsprint paper; colour markers

Course content

1. The health animator reminds the participants that malaria parasites are transmitted by mosquitoes from person to person, and that the mosquitoes can move from house to house to pick up or deliver the malaria parasite. Thus, community-wide action against malaria is important.
2. Remind the participants of the **identified problems** in a previous session.
3. Some problems can be addressed by each household themselves. Other problems need action by the entire community. Yet other problems require appropriate collaboration of the community with the government health service.
4. Ask one of the participants to describe (once more) the shared **vision on malaria**, as developed during the VCA session.
5. Ask the participants to brainstorm on what **actions should be taken** for the control of malaria in the village. Write the actions on the newsprint paper for the group to see. Consider the following categories:
    a. Actions at household level
    b. Village-level actions of malaria prevention
    c. Village-level actions of awareness raising
    d. Actions to promote early diagnosis, treatment and compliance
6. For each of the proposed actions, determine whether the action is of high **priority**, medium priority or low priority.
7. For here on, request the **village head** (if appropriate) to take the lead in the planning process, by using the following steps:
    a. Set a clear **goal**: What do we want to achieve after 4 years
    b. Which actions to take: Select the **prioritized actions**. Select only those that are feasible for implementation. Avoid that too many actions are selected, because this will not be realistic.
    c. Develop each action into a community-owned "**programme**". Give an appropriate name to each programme.
    d. Assign a **leader to each programme**, and ask (by raising of hands) who wants to participate in that programme. To select programme

leaders, ask the group to elect ‘champions’ who are motivated and capable for the role. The village head and animator have an overall supporting role.

Group discussion

- If time allows, ask the potential leader of each programme to share his or her views about the activities to be conducted, role of villagers and timing of activities.

8.4 Theater for development, songs, in malaria prevention and control

Learning objective

- Participants able to explain the potential role of songs and drama for raising malaria awareness among their villagers.

Materials

- Locally available objects for use in drama on malaria (bed net; other relevant objects)

Course content

1. Start by explaining that **awareness raising** of all village members about malaria is critical to achieving malaria control at village level. Each person has a role in preventing that malaria parasites continue to circulate within the village, by getting prompt treatment, and by avoiding being bitten by mosquitoes.
2. Explain that **songs or drama** can be used as an effective method to educate villagers about the subject of malaria and malaria control.
3. Ask about the **advantages** of using songs and drama, as compared to leaflets or lectures.
4. Songs and drama attract the **interest** of other villagers and, equally important, they contribute to **community cohesion** within the village. Community cohesion is important for initiating collective action on malaria control.
5. Explain the **example of a drama** that illustrates the transmission cycle of malaria, and how this cycle can be interrupted. Ask volunteers to come forward for the following roles:
    a. Person as villager who plays the person suffering from malaria
    b. Person given ‘wings’ who plays the mosquito
    c. A health officer
    d. Small object (such as a ball) that symbolizes the malaria parasite
    e. A bed net, bed to lay on
6. Ask the participants what would be a good **story to play** with these objects and actors. Think of a problem situation, and an improved situation (use of bed nets; treatment seeking, etc.).
7. If time allows, give the volunteers 15 minutes together to prepare a short drama on the malaria cycle.
8. Give the selected volunteers the task as ‘**homework**’ to work out the story in the form of a drama or song, and to practice with the idea to perform in an upcoming public meeting or during the next workshop.

Group discussion

- Ask participants whether a drama or song would fit as activity into the community action plan that was prepared during an earlier session.

ANNEXES

1. Participants list
2. Self-reporting on malaria
3. Malaria-adapted VCA manual

Annex 1. Participants list

Village name:			Date (dd-mm-yy)						
No.	Participant name	Gender	....-....-....	....-....-....	....-....-....	....-....-....	....-....-....	....-....-....	....-....-....
1									
2									
3									
4									
5									
6									
7									
8									
9									
10									
11									
12									
13									
14									
15									
16									
17									
18									
19									
20									
21									
22									
23									
24									
25									
26									
27									
28									
29									
30									
31									
32									
33									
34									
35									
36									
37									
38									
39									
40									
41									
42									
43									
44									
45									
46									
47									
48									
49									
50									

Village name: **Date (dd-mm-yy)**

No.	Participant name	Gender	....-....-....	....-....-....	....-....-....	....-....-....	....-....-....	....-....-....	....-....-....
51									
52									
53									
54									
55									
56									
57									
58									
59									
60									
61									
62									
63									
64									
65									
66									
67									
68									
69									
70									
71									
72									
73									
74									
75									
76									
77									
78									
79									
80									
81									
82									
83									
84									
85									
86									
87									
88									
89									
90									
91									
92									
93									
94									
95									
96									
97									
98									
99									
100									

Annex 2. Self-reporting on malaria

	Part A: Malaria cases		Part B: Health services received				
Village name: …………………………	Number of new cases since previous workshop		Number of cases				
**Date of workshop**	No. of persons under 5 yr	No. of persons 5 yr and above	Total	RDT done	RDT positive	LA administered	Other

Annex 3. Malaria-adapted VCA manual

Prepared by The Hunger Project – Malawi, July 2014

ABOUT THIS PARTNERSHIP: WHAT IS THE HUNGER PROJECT, MAJETE MALARIA PROJECT, AFRICAN PARKS MAJETE, AND THEIR RELATIONSHIP?

You as a facilitator/animator should fully understand the four key partner institutions of our work in this area, and how we come together. First and far most, it is the fact that the PPP arrangement between GOM and APM has enabled social investment in the communities around Majete Game Reserve. It is therefore important for all of us as we do our work to ensure that people see this connection and have a positive image of the Game Reserve.

The Hunger Project is not: An organization that distributes food for free; or a government organisation; or a representative of any political party or religion.

The Hunger project: is an organization which wants to ensure that people have a better future. A future where people are self-reliant in their country and in the whole world so that they have healthy and profitable lives. In brief, the hunger project's objective is to empower the rural poor communities to their own hunger and poverty and we believe that the answer to them lies in the wisdom that every person possesses.

Other organizations view poor people as poor and helpless beneficiaries in need of hand outs, but the Hunger project views poor people as solutions to their own problems. There is no person without the wisdom and dignity of humanity. All of us were created with dignity and freedom and being poor is not a punishment from God but it is an outcome of what we are doing. When we distribute things for free, it means we are destroying our self-reliance in life. Furthermore, free things do not have dignity.

Majete Malaria Project:

The Majete Malaria Project is a project that aims to drastically reduce the burden of malaria in the villages surrounding Majete Wildlife Reserve. This will be done by strengthening the health system, implementing effective interventions, and educating the communities through use of 'Health Animators' in all villages. A reliable supply of malaria diagnostic kits and medication will be provided to clinics and health posts in the area. Majete Malaria Project is a collaborative effort between the Malawi College of Medicine, The Hunger Project, Ministry of Health, African Parks, and several universities in Europe. The project started in 2014 and will end in 2018.

African Parks - Majete:

African Parks is a non-profit making organisation that takes on total responsibility for rehabilitation and long-term management of National Parks, in partnership with governments and local communities. In Malawi African Parks is managing Majete Wildlife Reserve. Since African Parks assumed management of Majete in 2003, more than 2,550 animals belonging to 14 species have been reintroduced, a fence surrounding the entire reserve has been erected, and 125 permanent and many more part time jobs have been created for the surrounding local communities. Majete is now regarded as a revolutionary and unfolding conservation success story and one of the

leading tourist destinations in Malawi. It is an excellent example of how wildlife conservation can go hand-in-hand with rural community development.

ABOUT THE VCA WORKSHOP PROCESS

This workshop is very important because:

- it provides an opportunity for people to meet and discuss matters. This consolidates their commitment.
- it enables people to understand that people cannot just seat helpless and accept malaria as a god-given curse, people can rise up and take coordinated action and work towards completely eliminating malaria from their communities.
- it enables people to understand the importance of being visionary and it also provides an opportunity for people to set up their vision for what they want to achieve in their lives and their communities as far as the fight against malaria is concerned.
- **This workshop should therefore aim at empowering people to be able to envision a new future “free from the scourge of malaria”**

WHAT ARE YOU CHALLENGED ON AS A FACILITATOR? TO FACILITATE A HIGHLY PARTICIPATORY PROCESS TO ENSURE THAT THE FOLLOWING HAVE BEEN ACHIEVED BY THE END OF THIS WORKSHOP

- People should change their way of thinking and should start believing that malaria can be combated and that as people who are affected by malaria, they are the ones with the power to end this challenges. Give examples of countries like The Netherlands where they have eliminated malaria and yet it is a land full of dams, waterways, and canals.
- People should understand the importance of good leadership for the activities that fight malaria.
- People should have a vision of how they are going to fight malaria and support the fight against malaria, and they should have a timeline of when they are going to achieve their vision.
- People should be self-reliant and proud to be Malawian and also for being able to assist the whole world.
- Participants should explain the activities they are going to be doing in their communities in order to realize their vision.

In brief, there are five steps to fight malaria:

1. Changing of mindset and believing that malaria is not a punishment from God and therefore can be eliminated.
2. Having a good leadership
3. Having a vision for our future free from the scourge of malaria
4. Commitment
5. Taking action (Hard work)

VISION, COMMITMENT AND ACTION (VCA) WORKSHOP MANUAL

Greetings

Firstly greet the participants attending the workshop and introduce yourself to them and allow the usual introduction formalities to be done. Inform participants of the objectives and the importance of this workshop.

Part 1: Changing our mindset

In Malawi, many of us black people believe that we were born in hunger and poverty, we also believe that we were born in a cursed land with heavy infestation of malaria and we will die like that. This is an absolute lie. There is nothing impossible on the face of this earth. As long as we have commitment, everything on this earth is possible.

As such, the first thing we are supposed to do is to engage ourselves and believe that malaria is not a punishment from God and that malaria can be a thing of the past if we believe and became self-reliant and hardworking.

Engage participants in a 'change of mindset' process for example use "lucky Netherlands" versus "unlucky Malawi" analogy or "black man" versus "white man" analogy

Part 2: Having a good leadership

Without good leadership, this work cannot be effective. As such, we are supposed to have leadership that is supposed to guide us in our work of fighting malaria. Leadership groups or individuals such as the Health Committee, Health Animators, CBOs, chiefs and other committees which can lead us in this fight against hunger and poverty and malaria need to be effectively supported and constituted wherever not available. Above all this, there is a proverb in our local language which says "*Tsokonombwe anatha mtunda ndikudumpha"* literally meaning *"this anima called Tsokonombwe covered a long distnace in small jumps"*. Before exercising community leadership, each one of us has to exercise leadership on ourselves as a person, on our households and on our community etc. *Give an example on how an individual can exercise leadership over himself as a person when it comes to malaria prevention and control*

This is an opportune point to intriduce the various leadership groups which we have in the area and dis cuss how important they are in this fight against malaria and how participants can work with them effectively. It is also a good point for you to engage participants in addressing any challenges that are being faced between the leaderships structures and the community in this fight

Part 3: Having a vision for our future

Most of us we don't realize the importance of having a vision for our future. We see it as a dream that cannot happen at all in our lifetime. Maybe you know this, may be you do not, but the truth is that everybody pursues a vision and it is this vision that guides us to do what we do in our everyday lives.

Some visions waste our time for instance deciding to drink beer and not work. Some people's visions indicate that they will have a future full of problems.

We at Hunger project say it is important for us to have a unified vision in Malawi. **For example** = Our vision can be something like this – **We the people of ……………… (indicate the name of your community) we want to eliminate malaria in …… years (indicate the years it will take)**

We have to have a vision that will give us a future just as how Nelson Mandela's vision brought freedom for South Africa. Our 1st President Dr Kamuzu Banda had many visions when he was at Gwero prison, one of which was, to have a road that would run from the north to the south region of Malawi. When he was released from prison, he swayed the whole country and a road called "M1" was constructed in many years and we all are proud of this road today.

Where there is no vision people are confused and they become failures.

What is a Vision?

A vision is a promise of how we want our future to be. We all need to know the following:

- It is possible to fulfill our vision.
- It is important for us to fulfill our vision.
- Having a vision improves our quality of life.
- Having a vision helps us remove fear and helps us to work hard.
- Having a vision removes hatred and disagreements that separates people and it unites us to find what we want.

Our visions are supposed to be based on the following points:

1. Our vision should not be representing any political party, it should include all of us regardless of our religion etc.
2. Our vision is for the future of our country Malawi and not for a small number of people, but for every child, woman and man of this nation.
3. Our vision is for the future that will be made by us the people of ……...(indicate the name of your community/village)
4. Our vision is the future that will be realized because of working together and not leaving things to government, organizations, banks and companies alone.

Activities

Now let us imagine (with our eyes closed) a changed future for ………. (put name of the area where you are doing the VCA), a beautiful future without malaria When we close our eyes we should be able to see in our vision that our community here …….. (indicate the name of your community) has no malaria or malaria- related deaths.

Part 4: Commitment

Even though we might have a good vision but if we are not committed, we might not realize our vision. The development of our communities is propelled by our vision. Commitment changes a person from poverty to prosperity. Commitment makes us use our skills and the hidden wisdom in us. To have commitment one needs to be fearless. Our everyday life needs courageous people who can fight for freedom. As such we need to commit ourselves wholeheartedly to the fight against malaria. We need to committ that we shall be available e and participate in all the modules of the malaria manual and shall not miss a single one. First, commit to yourself as a person, promise yourself, and then a committed person needs to openly show his or her commitment. **I therefore ask all those who say they are committed to fighting this war against malaria to stand up and show themselves to others.**

Facilitator: Tell people to clap hands for those who have openly shown their commitment. That is a sign of courage.

Part 5: Working hard with our own effort (Action)

Now we have reached the last part of our workshop. The last point is very important in this fight. When we have started working, it means we have started realizing our vision. The people who are supposed to work towards eradicating malaria are we the people affected by it. Let us not leave this to anyone else but let us be committed and work hard together with other people.

Some people in Malawi have achieved a lot of things in their communities by doing developmental work in unity. Such developmental work includes building of clinics, schools, bridges, agriculture etc. Hence it is time to realize our vision.

Facilitator, please explain the following points carefully

THE IMPORTANCE OF BEING IN A GROUP- Research which has been done has shown that people progress when they are in groups and discuss ideas.

SELF-RELIANCE – This is very important. People who have progressed are the ones who believe they can develop with what they have and not with what they do not have.

HONESTY – Being a person who tells the truth, does the right things, does not steal.

PARTNERSHIP – working together with others - rich, poor, men or women.

SKILLS – Having skills to do things. This is what differentiates us with animals. Being creative about things that can help us.

We should know the following things:

1. There are jobs/activities that we can do together us a family with or without money. This will help us build a better future.
2. There are activities that we can do together as a group. (Ask people to mention some of these activities, ensure that most of the examples should include those which are related to the fight against malaria)
3. When you form a group, you will realize that what you discuss will be heard more. You have power to engage and partner with government, banks and others.

What is missing?

One thing I would like to share with you is that we at Hunger Project-Malawi, start our work by asking ourselves: What is it that we are lacking? This is a question that can inspire us to work hard.

Discuss the development activities that have taken place in your community because of the unity and partnership of the people. Discuss the things people can do to develop their lives. Discuss this until you agree. Discuss how you can form partnerships and the people that can help you.

Now we are ready to start working. We have found an activity that we can do to help change our lives.

Finally, let us answer a few questions concerning how we are going to work:

- Who will lead us?
- What should we do first?
- When will we do this?
