# Supplementary material for: Community-based malaria control in southern Malawi: a description of experimental interventions of community workshops, house improvement and larval source management
Source: Malar J. 2018 Jul 16;17:266. doi: 10.1186/s12936-018-2415-1 (PMC6048888; doi:10.1186/s12936-018-2415-1)
Supplement: Supplementary file 2 — Additional file 2. Training manual on house improvement for malaria prevention and control. [file 12936_2018_2415_MOESM2_ESM.pdf]

Additional file 2

Training manual on house improvement for malaria prevention and control

a) Training manual for health animators on house improvement for malaria prevention and control (Phase A) – page 2

b) Manual on house improvement (Phase B) – page 22

c) Manual for training workshops for HI committee members at cluster-village level (Phase C) – page 30

TRAINING MANUAL
FOR HEALTH ANIMATORS
ON HOUSE IMPROVEMENT
FOR MALARIA PREVENTION
AND CONTROL

(PHASE A)

Majete Malaria Project

Majete Malaria Project – Community-based malaria control in the perimeter of Majete Wildlife Reserve

PREFACE

This manual has been developed by the Majete Malaria Project, as a collaborative effort between its project partners. Drafting of the document was by Henk van den Berg, with inputs provided by Rob McCann, Zinenani Truwah, Monicah Mburu, Alinune Kabaghe, Asante Kadama, Tumaini Malenga, Mackenzie Nkalapa, Saidon Banda, Tinashe Tizifa, Michele van Vugt, and from the group of health animators.

This manual was first produced in June 2015. The manual is available in the English and Chichewa language. Funding for development of this manual was provided by Dioraphte Foundation, the Netherlands. Requests for permission to reproduce or translate this document – whether for sale or for non-commercial distribution – should be addressed to the programme manager (e-mail: pmthrue@medcol.mw).

INTRODUCTION

This manual is an addition to the malaria manual, by the Majete Malaria Project in collaboration with The Hunger Project. This manual is meant for use in implementation **only in those villages that have been selected** for house improvement during the community ballot box event.

Malaria mosquitoes can be much more numerous in some houses than in others, and previous studies have found that people living in **poor housing** had an **increased risk of malaria**. Improvement of houses plays an important role in reducing the presence of malaria mosquitoes indoors. This will help reducing transmission of the malaria parasite. This could provide an **extra line of protection**, in addition to bed nets, but NOT replacing the bed nets. Methods of house improvement include the screening and closing of eaves, windows and doors

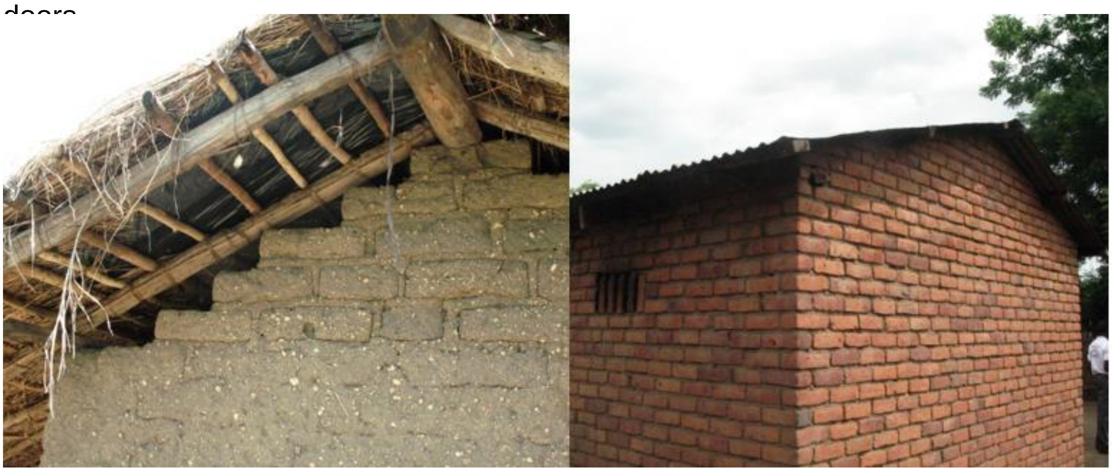

Two major types of houses in Majete: thatched roof with sun-dried bricks; and iron roof with baked bricks. The former is subject to major house improvement, including adding of bricks and mud. The latter needs minor improvements only. Both need iron mesh to maintain air circulation inside the house.

House improvement will be an intervention that is **implemented by the community** themselves. To achieve this, the communities need to be educated and empowered to make a change in their own housing situation.

Health animators have the important task to enable the community to voluntarily **adopt** house improvement for malaria control.

As in the overall MMP project, **THP-Malawi** is responsible for recruiting the health animators, managing the health animator group on the ground, and training of the health animators in the conduction of village workshops.

The **Health and Education Unit** (HEU) of MoH is responsible for assisting the development of information, education and communication (IEC) materials, assisting the training of health animators, annual supervision of the health animator process and health promotion policy.

The **MMP** is responsible for the technical aspects of training of the health animators.

Objective

The objective of this curriculum is to provide people living in selected villages with the **knowledge, tools and motivation** to implement house improvement village-wide.

This training is part of an intervention of a research trial, in which the purpose is to assess the impact of community-driven house improvement on reducing transmission of malaria parasites.

Use of this manual

This manual is used in the **training of health animators**. This same manual will also be used for **conducting the village workshops** on house improvement.

Chapter 1 of the manual addresses general aspects of organizing and planning the village workshops. **Chapter 2** presents the main topics for each village workshop.

1. HOW TO ORGANIZE AND PLAN

The village head is a **key person** in the activities on house improvement. The role of the village head is to give **support in coordination, organization and planning** of the community-level activities. Without this leadership support, it will be very difficult to achieve village-wide house improvement. The health animator should always timely **inform** the village head and **request his participation** in the village workshops on house improvement.

The village workshops on house improvement will follow the same model as the ongoing village workshops on malaria prevention and control. The ongoing workshops are **every two weeks** at an agreed location. The new village workshops on house improvement will be held in the week **in between the ongoing malaria workshops**. So, one week there will be a malaria workshop, the next week a house-improvement workshop, the next week a malaria workshop, and so on. This is illustrated in the figure below.

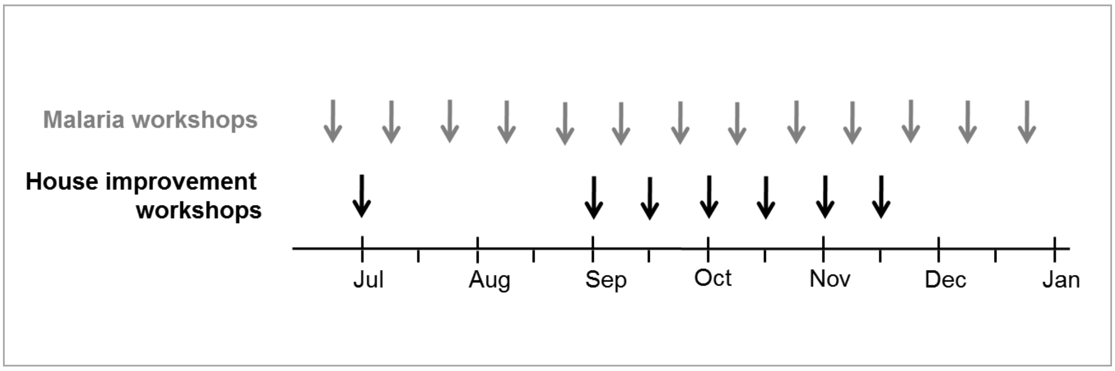

In preparation of the community workshops, the health animator should have the **required materials** ready in advance of the workshops. The materials are indicated in Chapter 2.

During the ongoing malaria workshops in the village, the health animator should make an **announcement** when the first workshop on house improvement, to be held early July, will start. Villagers should be reminded of the community event, in which villages targeted with house improvement were selected through a lottery.

The health animator should carefully plan the dates for each topic of the course content. A provisional plan is presented in Table 1.

The workshops are divided into several categories, or 'blocks'. The first is on **preparation**, which is to be held early July. The second block is related to **action planning**. The third block is the **rolling out** of the house improvement activities, which is the most important part. Finally, the fourth block is the **maintenance** phase, which is not through separate workshops on house improvement, but as a small add-on to the ongoing malaria workshops.

Table 1. Plan for the course content of village workshops on house improvement (from July 2015). Chapter numbers are indicated for easy reference.

Block	Workshop	Time	Course content	Manual chapter
Preparation	1	Jul'15	Housing situation and brick baking	2.1
Action planning	2	Sep'15	Mosquito behaviour of house entry	2.2
	3	Sep'15	Methods of house improvement	2.3
	4	Oct'15	Community organizing and action planning	2.4
Rolling out	5	Oct'15	First evaluation of progress	2.5
	6	Nov'15	Second evaluation of progress	2.6
	7	Nov'15	Third evaluation of progress	2.7
Maintenance	*	from Dec'15	Maintenance of house improvement	2.8
	*	from Dec'15	Closing of door and windows after dark	2.9

* This activity will not be in the form of separate dedicated village workshops, but as an add-on topic in regular ongoing village worshops on malaria

The **workshop agenda** is quite similar to that of the malaria workshops. The only difference is that there is no agenda item on ‘self-monitoring of malaria’ (Table 2). The agenda items have been explained in detail in the malaria manual, and will not be repeated here.

Table 2. Agenda items for community workshops on house improvement; total duration approx. 2 hr.

Nr.	Workshop agenda item	Min	Notes	Self monitoring
1	Opening remarks and workshop agenda	10		Has the agenda been explained?
2	Introducing participants	10	Secretary completes participant record sheet	Are new participants introduced?
3	Summary of previous workshop	5		Is summary of previous session given?
4	Course content	45	See course content per session*	Are the key points presented clearly?
5	Group discussion	30	See questions related to course content*	Does a lively group discussion take place?
6	Plan for next workshop, and closing	5	Create expectation and encourage participation	Is next session's topic announced?

* See course content according to the schedule in table 1

The participant list should be completed in each village workshop on house improvement. This will assist the health animator in identifying households that do not participate. Participant lists are also needed for monitoring purposes. The participants list is kept by the secretary. An example of a participant list is provided in **Annex 1**.

2. COURSE CONTENT

2.1 Housing situation and brick baking

This village workshop should be held early July 2015, as a preparation activity.

Objective

- To prepare the villagers for their involvement in house improvement, by initiating brick baking

Materials and preparation

- Prior to the village workshop, discuss the course content with the village head, and ask him to take a leading role in coordinating the baking of bricks.
- This workshop should serve as the **launch** of the house improvement campaign in the village. Therefore, the date of the workshop should be **announced** to the villagers ahead of time, and a special agenda item (such as a drama on house improvement, or a festive activity) should be added to raise the profile of this workshop.

Course content

1. Remind participants about the lottery event in which this village was selected for house improvement. Ask who were not aware of this.
2. Ask: Why should we try to keep malaria mosquitoes outside of our houses?
3. Ask: How could we stop mosquitoes from entering our houses?
4. Explain that **recent research in Kenya** has showed that closing of all the openings in each house kept most of the malaria mosquitoes out. Only very few (10%) still entered.
5. Explain that some houses in our village have **many openings** through which malaria mosquitoes can enter. Ask participants to indicate the openings (e.g. between wall and roof, openings in windows and doors, ventilation openings)
6. Ask: How can we **close those large openings** between the wall and the roof in some houses? Could we use bricks and mud?
7. Inform that the malaria project will be able to provide a **small amount of iron gauze**, to close small openings kept for ventilation. Other than that, house improvement will be a **self-help activity**, by the community themselves
8. Show and explain the visual:

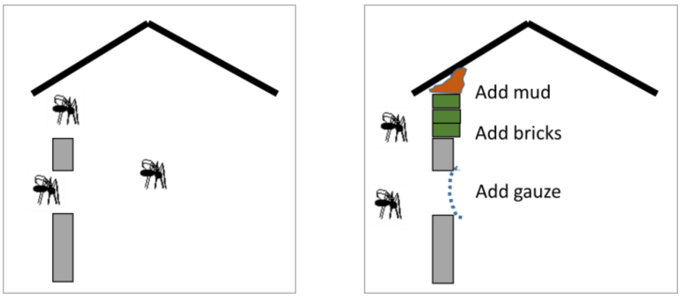

Before house improvement:
Mosquitoes can enter the house

After house improvement:
Mosquitoes stay outdoors

9. Explain that the training on house improvement only starts in September, but before the training starts, we need to make some **preparations**
10. Can we prepare sufficient **bricks during the dry spell**, so we can fill the big openings between the wall and the roof? Can this be done by each household, or as a community effort by neighboring households helping each other?
11. Let's make an assessment of what is needed: **How many houses** are there in each household?
12. And how many of those houses have a **large gap between the wall and roof** (requiring filling with bricks)?
13. **How many bricks** will each household need? And, should these be sun-dried or baked bricks, or both?
14. Ask the participants whether they or their neighbors can make bricks, or whether they need help with producing the bricks
15. Ask the village head to **request 10-20 volunteers** (more if needed) who are able and willing to help with producing the bricks during the month of July. Ask the secretary to record their names
16. Ask the volunteers to stay after the closure of the village workshop so that they can together with the village head **prepare an action plan** for brick baking. The health animator should ensure that the action plan will be realistic, and that it will get implemented

Group discussion

1. What **difficulties** do you foresee in the production of the bricks, and how could these difficulties be resolved or prevented?
2. How will the bricks be **divided among the houses** in the village (or cluster)? Will this likely result in quarrel or strive? If yes, how can this be avoided?

2.2 Mosquito behaviour of house entry

Objective

- Participants understand mosquito behaviour of house entry

- Participants understand the concept of two lines of protection against malaria transmission

Materials

- Visual material showing 2 lines of protection

Course content

1. Remind participants of the **role of mosquitoes** in the transmission of the malaria parasite: without the mosquito there cannot be malaria circulating within the community
2. Female mosquitoes search for humans when they are **hungry for a blood meal**
3. They can trace humans from a distance because they have an excellent **sense of smell**
4. Ask participants about the differences between **nuisance mosquitoes** (*Culex*) and **malaria mosquitoes** (*Anopheles*) (when do they bite, which are the noisy ones, which are dangerous?)
5. Most malaria mosquitoes enter the house **through the eaves**, which are the openings between the wall and the roof. Some also enter through windows or other openings
6. In search of a house, the female mosquito travels low to the ground, and when arriving at the wall of a house, she **moves upwards, towards the eave** (or windows), where she enters the house
7. Why does the mosquito move upwards? She follows the **smell that comes out of the eave** openings of the house. She recognizes the smell of humans sleeping inside the house
8. Ask the participants: Are **bed nets** a full-proof protection against malaria mosquitoes or should we also close our houses for mosquitoes? For example:
    a. What if we are bitten <u>before</u> going to bed?
    b. What if we bitten <u>through</u> the net?
    c. What if there are <u>openings</u> in the net?
    d. What if the number of nets is <u>not sufficient</u> to cover all sleepers?
9. What could we do to **close our houses** for mosquitoes, and avoid mosquitoes entering our houses at night? (How could we close the house?)
10. Explain that bed nets are one ‘line of protection’ and that house improvement will be our **second ‘line of protection’** against malaria transmission (even if the house is closed, bed nets should still be used!)
11. Show and explain the **visual**:

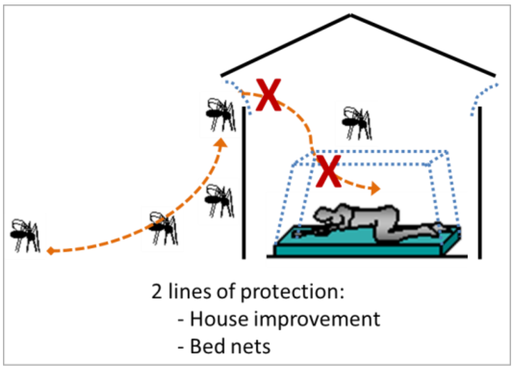

12. Ask: Will **two lines of protection** (bed nets + house improvement) be better than one line of protection? (Will it better protect us against malaria transmission?) Why?
13. Explain that a **recent research in Kenya** has showed that closing of all the eaves in each house with mosquito gauze (wire) kept most of the malaria mosquitoes out. Only very few (10%) still entered
14. Our village was selected to join in the **first research in Malawi** on house improvement. The purpose of this research is to find out whether house improvement reduces the transmission of malaria. Two years from now, if the result is positive, house improvement may come to many more villages
15. It is important that all households in the village collaborate in house improvement

Group discussion

- Is it possible to close our houses from mosquitoes? Can we achieve this as a community effort?
- Do we need openings in our houses for ventilation during the night? If so, can we use mosquito gauze to close these openings but keep the air flow?

2.3 Methods of house improvement

Objective

- Participants understand the correct methods of house improvement

Materials

- Visual material showing house improvement of eaves
- Sample of mosquito gauze

Course content

1. Remind participants of the behaviour of malaria mosquitoes: they enter the house from the outside wall by **moving up to the eaves** (opening between wall and roof), following the smell of humans who are sleeping indoors. If we close the eaves, most mosquitoes will not enter the house

2. Ask participants to differentiate the **types of houses** found in the village. For example:
    a. Sun-dried bricks with thatched roof houses
    b. Baked bricks with iron corrugated roof houses
3. Which type of house will have **more mosquitoes**, and why? Can those living in these house types testify this?
4. Ask: How important is it to have **openings for ventilation** at night?
5. Explain that the use of **mosquito gauze** will keep mosquitoes out but retain ventilation. This material is **expensive**, and its use should be restricted to essential ventilation openings only. All other openings not needed for ventilation should be closed
6. Discuss how we can **close the eaves** so that mosquitoes do not enter (e.g. with bricks and filling up with mosquito gauze)
7. Large openings should first be **filled with bricks**. On top, mosquito gauze can be fitted to allow ventilation
8. Show and explain the visual:

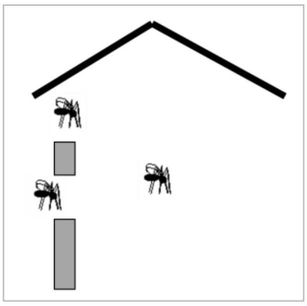

Before house improvement:
Mosquitoes can enter the house

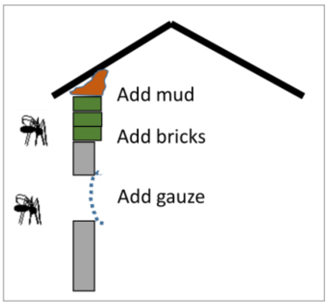

After house improvement:
Mosquitoes stay outdoors

9. How can we close the **small openings** that are often found in the walls (e.g. with mud; mosquito gauze).
10. It is better to **fit gauze from the outside**, not from the inside, because mosquitoes **enter from that direction** (see visual) (if we fit the gauze from the inside, it could create a funnel which the mosquito could follow to reach inside)
11. Discuss how to make **windows** mosquito proof (e.g. by sealing with bags). Can this be done with available local materials?
12. Can **doors** be made mosquito proof?
13. **Show an example** of mosquito gauze, and how it can be rolled to be fitted in small eaves. Explain that each household will receive a fixed amount of mosquito gauze (amount to be determined later)
14. This material should exclusively be used for house improvement, **not for other purposes**, or traded. Monitoring visits will verify the correct use of the mosquito gauze in each house
15. As **homework for the next meeting**, ask participants to make an assessment of the openings in their houses, and what it takes to close the houses for mosquitoes

Group discussion

- Discuss the **advantages** of sleeping in a house that is closed against mosquitoes
- Discuss what householders can **do themselves** to close their house for mosquitoes
-

2.4 Community organizing and action planning

Objective

- Participants organize themselves and agree on roles
- Participants prepare a plan for village-wide implementation of house improvement

Materials

- Flip-chart paper with markers

Course content

1. Remind participants of the **plan of house improvement** to prevent malaria mosquitoes from entering the house at night
2. House improvement is a community effort, aiming to reduce the malaria burden in the **entire village**. This collaboration will make the village stronger and united in the fight against malaria
3. Outline the task ahead:
    a. **All houses** should be improved before the rains start
    b. All households have a **role to play**, by improving their own house, and in assisting others in improving their houses
    c. **Leadership** is needed to coordinate the efforts
4. Explain what is meant by a **community effort**. Not every household will be able to do the house improvement by themselves. Some people may lack the skills or time. In such case, neighboring households can be called upon for help. **Households should help each other**, because they all will benefit by having less malaria in the village.
5. Conduct the next steps in consultation with the **village head**, so that he is closely involved - or in charge
6. The overall **leadership** is with the village head – his role is to oversee the house improvement in the village. The health animator will be the organizer of the house improvement activities
7. To assist the village head and health animator, there should be a special '**house improvement team'** of volunteers. These are four to six energetic persons who, between them, have the following skills:

a. Organizing skills
b. Workmanship
c. Communications skills

8. The **tasks** of the house improvement team would be:

    a. To **stimulate villagers** to implement house improvement, and to help their neighbors

    b. To **provide help in exceptional cases** where householders and their neighbors are all unable to do house improvement

    c. To ensure that **bricks and mosquito gauze** are fairly distributed and correctly used

    d. To **disseminate best practices** of house improvement

    e. To **monitor progress** in the village, by **keeping records** on the

        - Number of houses that have initiated house improvement, and

        - Number of houses that have completed house improvement

9. Ask for **nominees** for the house improvement team. Ask participants to make the selection. The workshop secretary should note down their names

10. On flipchart paper, draw a simple sketch **map of the village** (or cluster). Indicate important landmarks (rivers, roads) and areas where most houses are found. Use this map for the action planning

Group discussion (most important part)

1. **Develop the action plan** for house improvement, keeping in mind that all houses should be completed before the rains. Use the following questions as guideline

    a. When to start house improvement?

    b. Where to start house improvement (which part of the village on the map)?

    c. Are sufficient bricks available to each household?

    d. How will the mosquito gauze be distributed equitably to each household?

    e. Do householders need any help in house improvement?

    f. What should be role of their neighbors in assisting in house improvement?

    g. What is expected from the house improvement team?

The health animator, secretary and village head should **meet with the house improvement team directly after the village workshop** to give further clarifications on their role and responsibility

2.5 First evaluation of progress

Objective

- Participants aware of progress made on house improvement in the village
- Participants stimulated to plan and implement further activities

Materials

- Flip-chart paper with markers

Course content

1. Remind participants that the purpose of house improvement is to **keep malaria mosquitoes out** of the house. This helps prevent malaria transmission during the night.
2. House improvement is a **community effort**, and all households of the village (or cluster) should join.
3. Ask: **Why is it important that all households join** in the house improvement activity? Suppose that few houses are left open, while all other houses are closed for mosquitoes, what would be the effect? (e.g. those in open houses would be at higher risk of malaria, which could keep malaria transmission going on in the village)
4. Ask the village head to update us on the **availability of bricks and mosquito gauze**, and how the distribution of these materials has been going
5. Ask the members of the house improvement team to take the floor and **report on the progress** made in the village on house improvement in the past two weeks
    a. In how many houses house improvement been **initiated**? And in how many houses has this not yet been initiated?
    b. In how many houses has house improvement been **completed**?
    c. What are the major **challenges** faced in house improvement so far?
6. Prepare a simple **bar graph** on a flipchart paper, as in the figure below. Discuss the proportion of the houses that have initiated, or completed, house improvement. Indicate this in the 'barometer'. Ask: how far are we from completing all houses in the village?
7. Ask one or two villagers to explain **how house improvement was conducted** in their house, and what can be learnt from their experience
8. Ask someone to tell about his **experience in helping his/her neighbor** with house improvement (not in his/her own house)
9. Now ask the participants to raise their hands **who have started** with house improvement

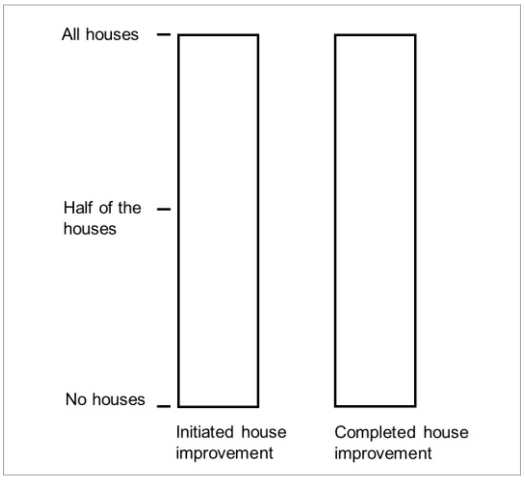

Group discussion

- In the coming two weeks, **how many more houses** should be completed?
- What are the best **solutions for the challenges** that have been faced?
- How do we reach those villagers **who do not actively participate**? What can be done to involve them in house improvement?
- How is the **action team** performing? Do they need additional members or new members?

2.6 Second evaluation of progress

Objective

- Participants aware of progress made on house improvement in the village
- Participants stimulated to plan and implement further activities

Materials

- Flip-chart paper with markers

Course content

1. Remind participants that all households should be **united in the fight against malaria** in the entire village. This means to work together on house improvement as a community effort
2. Ask the village head to update us on the **availability of bricks and mosquito gauze**, and how the distribution of these materials has been going
3. Ask the members of the action team to take the floor and **report on the progress** made in the village on house improvement in the past two weeks
    a. In how many houses house improvement been **initiated**? And in how many houses has this not yet been initiated?
    b. In how many houses has house improvement been **completed**?

c. What are the major **challenges** faced in house improvement so far?

4. Present the **bar graph** on flipchart paper, which was prepared during the previous workshop. Update the proportion of the houses that have initiated, or completed, house improvement. Ask: how far are we from completing **all** houses in the village?

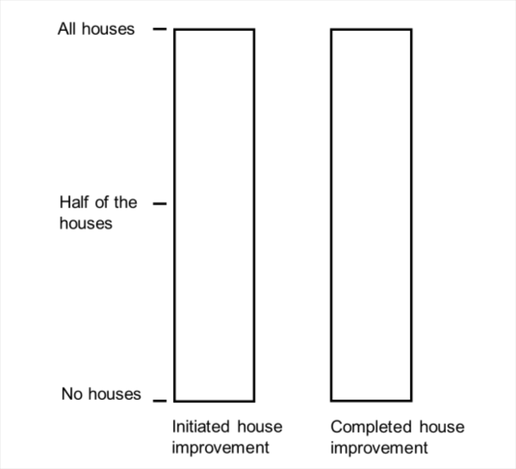

5. Ask one or two villagers to explain **how house improvement was conducted** in their house, and what can be learnt from their experience
6. Ask someone to tell about his **experience in helping his neighbor** with house improvement
7. Now ask the participants to raise their hands who have **not yet started** with house improvement
8. Are there households or parts of the village that do **not participate** in the workshops and are not aware of the house improvement campaign? How can we reach them?

Group discussion

- In the coming two weeks, **how many more houses** should be completed?
- What are the best **solutions for the challenges** that have been faced?
- How is the **action team** performing? Do they need additional members or new members?

2.7 Third evaluation of progress

Objective

- Participants aware of progress made on house improvement in the village
- Participants stimulated to plan and implement further activities

Materials

- Flip-chart paper with markers

Course content

1. Remind participants that all households should be **united in the fight against malaria** in the entire village. This means to work together on house improvement as a community effort
2. Ask the village head to update us on the **availability of bricks and mosquito gauze**, and how the distribution of these materials has been going
3. Ask the members of the action team to take the floor and **report on the progress** made in the village on house improvement in the past two weeks
    a. In how many houses house improvement been **initiated**? And in how many houses has this not yet been initiated?
    b. In how many houses has house improvement been **completed**?
    c. What are the major **challenges** faced in house improvement so far?
4. Present the **bar graph** on flipchart paper, which was prepared during the previous workshop. Update the proportion of the houses that have initiated, or completed, house improvement. Ask: have we managed to complete all houses in the village?

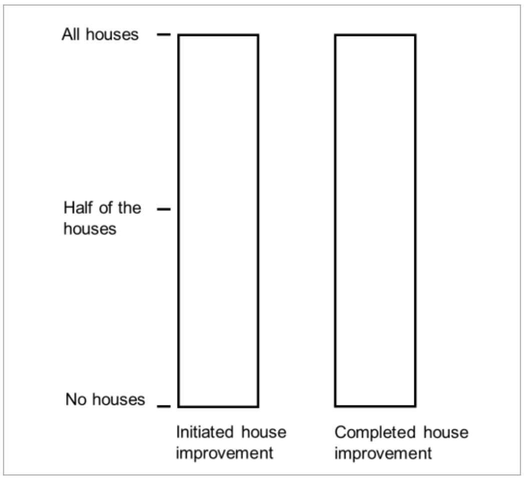

5. Ask one or two villagers to explain **how house improvement was conducted** in their house, and what can be learnt from their experience
6. Ask someone to tell about his **experience in helping his neighbor** with house improvement
7. Now ask the participants to raise their hands who have **not yet started** with house improvement
8. Are there households or parts of the village that do **not participate** in the workshops and are not aware of the house improvement campaign? How can we reach them?

Group discussion

- Discuss whether the task of village-wide house improvement has been achieved, or **whether further action is needed**. If yes, plan how this could be achieved without much delay

- Are the **effects of house improvement** to people inside the house at night noticed? What are the effects?

2.8 Maintenance of house improvement

How to use

- This is a very brief set of questions, to be added to ongoing malaria workshops. These questions do not need to be added every two weeks, but only **once per month**

Objective

- Participants motivated to **continue to maintain and improve** the house so that mosquitoes cannot enter

Course content

1. Remind participants that the purpose of house improvement is to **keep malaria mosquitoes out** of the house. This helps prevent malaria transmission during the night.
2. Ask participants whether they notice **more or less mosquitoes** indoors. Why is this so?
3. Explain the importance of regular (monthly) **inspection** of the house by householders to check if the house is still mosquito-proof.
4. Mosquito gauze could become **loose** or be removed, or windows could become **poorly sealed**. This would allow mosquitoes to enter.
5. Ask one or two participants who have recently inspected their house, to tell about their **findings and actions**.
6. Encourage participants to maintain the closed condition of the house, and announce that persons from the action team or from the Majete Malaria Project **may come to visit** and monitor their houses.

2.9 Closing of door and windows after dark

How to use

- This topic should be added to ongoing malaria workshops.

Objective

- Participants understand the importance of closing door and windows after dark to keep malaria mosquitoes out.

Course content

1. Remind participants of the **campaign of house improvement**, aiming to keep malaria mosquitoes out of our houses at night.
2. Malaria mosquitoes can transmit malaria parasites from a person to another.
3. Ask: How do we **keep mosquitoes out** of our house?
4. Explain that malaria mosquitoes become **active soon after dark**. The female mosquitoes move towards our house and try to enter the house with humans inside. Their aim is to get a blood-meal from a human.
5. Ask: What if our house has closed eaves and screened windows? Will mosquitoes be able to enter?
6. Ask: What if the **door remains open** after dark. What if we leave the window screens or windowpane open after dark? Will mosquitoes be able to enter?
7. Invite one or two **participants to explain** at what time the doors and window screens are closed in their house (how many hours after dark).
8. Explain the "**best practice**" of closing doors and window screens after dark, because malaria mosquitoes are active at that time. Closing the door and window screens will prevent mosquitoes from entering our house.
9. House improvement depends on two things: the house condition and our behavior. Our **house condition** has been improved, with closed eaves and screened windows. Now we also need to improve our **behaviour** of keeping doors and window screens closed after dark.
10. Remind participants that bed nets are the first 'line of protection' against malaria transmission. Bed nets should be used at all times. House improvement is our **second 'line of protection'** against malaria. Together, bed nets and house improvement give us better protection against malaria.

Group discussion

- Discuss whether it is **possible** for households to keep their doors and window screens closed after dark. Or, why may it be difficult to achieve this?
- Discuss how we can increase awareness and **change behavior** to close doors and window screens after dark in our village?
- Are people **sleeping outdoors** during the hot season? If so, will mosquitoes be able to find them? How to protect against the bite from malaria mosquitoes?

ANNEX 1. PARTICIPANTS LIST: HOUSE IMPROVEMENT

Village name:			Date (dd-mm-yy)						
No.	Participant name	Gender	….-….-….	….-….-….	….-….-….	….-….-….	….-….-….	….-….-….	….-….-….
1									
2									
3									
4									
5									
6									
7									
8									
9									
10									
11									
12									
13									
14									
15									
16									
17									
18									
19									
20									
21									
22									
23									
24									
25									
26									
27									
28									
29									
30									
31									
32									
33									
34									
35									
36									
37									
38									
39									
40									
41									
42									
43									
44									
45									
46									
47									
48									
49									
50									

Village name: **Date (dd-mm-yy)**

No.	Participant name	Gender	….-….-….	….-….-….	….-….-….	….-….-….	….-….-….	….-….-….	….-….-….
51									
52									
53									
54									
55									
56									
57									
58									
59									
60									
61									
62									
63									
64									
65									
66									
67									
68									
69									
70									
71									
72									
73									
74									
75									
76									
77									
78									
79									
80									
81									
82									
83									
84									
85									
86									
87									
88									
89									
90									
91									
92									
93									
94									
95									
96									
97									
98									
99									
100									

MANUAL ON HOUSE IMPROVEMENT (PHASE B)

Preface

House improvement in selected villages was started in July 2015 with brick making, followed by closing of the large gaps between the wall and roof (eave), and screening of windows with wire mesh. After completion of these operations, project staff observed that small openings are still remaining in the eaves through which malaria mosquitoes could enter. Previous studies have shown that malaria mosquitoes prefer to enter a house through the eave. Therefore, a second phase of the house improvement campaign will be started with special focus on sealing all small openings in the eaves of houses.

1. Proposed timetable

The timeline of activities for the second phase are from August to September 2016. By mid-October 2016, the sealing of eaves in houses in the selected villages should be completed.

Early/mid August 2016	Meeting at focal area-level (FA-A, FA-B, FA-C)
Mid/late August 2016	Meeting at HI committee level (by health animators)
Late August 2016	Establishing a Demonstration House
Late August/early Sept	Village event
September 2016	Implementation
Sept-Oct 2016	Monitoring and support

2. Meeting at focal area-level

Time:	Half-day meeting, early/mid August 2016; one meeting in each focal area
Venue:	To be determined by EPO

Participants:

a. EPO
b. Health animators and chiefs (from HI villages only)
c. MMP/COM supervision staff (e.g. Site Coordinator)
d. MMP/COM Research Assistant
e. Representatives of each HI committee (chair + 1 or 2 others)
f. If possible: HSAs, THP

Objectives

1. To agree on the need for further improvement of houses
2. To plan activities and roles for Phase B of the house improvement campaign

Meeting agenda

1. Introduction session: Use Chapter 4 as guideline (30 min)
2. Establishing a Demonstration House: Briefly discuss the contents of Chapter 5 (20 min)
3. Village event: Briefly discuss the contents of Chapter 6 (20 min)
4. Monitoring and support: Discuss the contents of Chapter 7 (20 min)
5. Planning session (45 min):
    a. For each activity, set tentative dates, and define roles
    b. Use the timetable in Chapter 1 as guidance
6. Special considerations: Emphasize that this is a community-driven activity, based on voluntary action. People must be encouraged to improve their houses, but they should never be forced to do so.
7. Conclude the meeting

3. Meeting at HI committee-level

Time: Mid/late August 2016

Venue: Each HI village

Participants: EPO, Health animators of the HI village, chief, HI committee members, HSA (if possible)

Objective

- To plan activities for Phase B of the house improvement campaign

Meeting agenda

1. Introduction session: Use Chapter 4 as guideline (30 min)
2. Establishing a Demonstration House: Discuss the contents of Chapter 5, and identify a Demonstration House (40 min)

3. Village event: Discuss the contents of Chapter 6 (40 min)
4. Monitoring and support: Discuss the contents of Chapter 7 (20 min)
5. Planning session (45 min):
    a. There are four activities, all of which should be completed by end September 2016:
        i. Establishing a Demonstration House
        ii. Village event
        iii. Implementation
        iv. Monitoring and support
    b. Discuss for each activities **when** it should be conducted, who is **responsible**, and who should be **involved**
6. Conclude the meeting

4. Introduction session

1. Remind participants that the first phase of house improvement has been a **major achievement**. This shows what is possible through collaborative effort. The EPO, health animators, village chiefs, HSAs, committee members, and villagers have all contributed to this important result. The windows have been screened. The eaves of houses have been closed or partially closed. As a result, fewer mosquitoes will enter the houses.
2. Ask: Is house improvement now complete? No. Some mosquitoes can still get into the house. Particularly in houses with thatched roofs, there are still a number of **small openings in the eaves**, or a narrow slit along the eaves. The mosquitoes are small enough to enter through these openings.
3. Malaria mosquitoes prefer to enter the house through the eaves. Therefore, it is most important that the eaves are properly sealed. (Note that this is different from “nuisance” mosquitoes, which enter houses through doors and windows more than malaria mosquitoes do).
4. Ask: How do malaria mosquitoes know there are **people inside** the house? The mosquitoes can smell that there are people inside. They have a much better sense of smell then humans. When there are openings in the eaves, the smell comes out of them. The mosquitoes follow the smell and enter inside.
5. Ask: **How to observe** whether there are small openings in the eaves? Small openings in the eaves are best observed from the dark inside of the house, against the back-light.
6. Ask: How can these small openings be **sealed**? This could be with mud or bricks. This could be done from the outside or the inside, or both. It may be easier to close the openings from the outside, which is also the side from which the mosquitoes enter. However, it should always be checked from the inside whether any small openings are remaining to be closed.

7. Explain that we will now start **Phase B** of house improvement, which will focus on sealing the small openings in the eaves. The objective is to prevent that any malaria mosquito will be able to enter the house at night.
8. Phase B will be through use of a **Demonstration House**. A demonstration house is a house that will serve as an example on how to seal the windows and eaves. A Demonstration House should be open for visits by other villagers on Open House days, which are the day agreed during the campaign.
9. Explain that this campaign of House Improvement (Phase B) consists of three parts:
    a. <u>Visit to Demonstration House</u>. The villagers **visit** the **Demonstration House** on its "Open House" days.
    b. <u>Implementation</u>. Then, all households in the village start **indoor inspection** of their houses for small openings in the eaves, preparing strong **clay** or mud, and methodically **sealing all small openings** in the eaves until no more openings can be seen from inside the house.
    c. <u>Monitoring and support</u>. HI committee members will **monitor** the houses and advice residents whether house improvement is according to the standard or whether further support is necessary.

5. Establishing a Demonstration House

The Demonstration House will serve as example to all villagers on how the eaves should be properly sealed. The Demonstration House must therefore be open to villagers for visits during special occasions.

Time

The Demonstration House should be made ready in Late August 2016, so that the village campaign can start late August or early September. A Demonstration House should first be selected, and only then be properly sealed.

Selecting a Demonstration House

When selecting a Demonstration House, several criteria must be considered:

1. The house must have a <u>thatched roof</u>, and a door that can be properly shut.
2. The house must be within easy reach for all villagers
3. The house owner is a strong supporter of house improvement, and is preferably a member of the HI committee
4. The house owner will allow access of villagers to visit the house indoors and outdoors during Open House days. These Open House days are the days agreed for visits during the village campaign.
5. One Demonstration House may suffice for most villages, but two Demonstration Houses may be required for larger villages

Preparing a Demonstration House

To prepare a Demonstration House, follow these steps:

1. Ensure that all windows are screened, and that the mosquito wire is in-tact and properly nailed to the wall or window frame. If there are small openings around the edge of the window, seal them with mud.
2. From the inside of the house, check the eaves on all four sides, and look for any small openings, or slits
3. Prepare a strong adhesive clay or mud material, and use it to carefully seal all small openings in the eaves, up to the roof material
4. Double check from the inside of the house whether any openings are remaining that should be closed
5. Clean up personal items so that visitors can easily enter the house

6. Village event

Objective

- To launch the second phase of the house improvement campaign, and to plan the activities ahead

Time

Late August/early September 2016

Preparation for the village event

1. Select a suitable day in consultation with the Chief
2. Organize a theatre or dance on the theme of “malaria and house improvement”
3. Announce the village event to all villagers a few days in advance, again one day in advance, and again on the day itself
4. The following suggestion is given for the agenda:
    a. Introduction session
    b. Theatre or dance
    c. Planning session
    d. Discussion
    e. Visit to Demonstration House

Introduction session

- Present Chapter 4 from the manual

Theatre or dance

- Have a theatre or dance on the theme of “malaria and house improvement”

Planning session

1. Explain that all villagers should try to **visit the Demonstration House**. Not all villagers can visit at once, because it would be too crowded. Therefore, a few days are available as "Open House" days. Announce these "Open House" days, and assign different parts of the village to visit at each "Open House" day.

2. **Implementation**. Explain that after the visit to the Demonstration House, all households in the village should start indoor inspection of their own houses for small openings in the eaves. This should be done in the month of August. They should prepare strong clay or mud, and methodically seal all small openings in the eaves until no more openings can be seen from inside the house.

   a. Discuss how to prepare a strongly adhesive mud or clay, that does not easily wear down.

   b. Ask which households will NOT be able by themselves to seal the openings in the eaves, but need help from the HI committee. The committee members should take note of these households and follow up with them directly after the meeting.

3. **Monitoring and support**. Inform the participants that by late August (or early September), the committee members will check each house whether its eaves have been properly closed.

4. Summarize the main activities for the coming month: Visit of Demonstration House; Implementation in people's own houses; and Monitoring and support

Discussion

- Discuss the plan with the participants. Obtain feedback and make adjustments to the plan where appropriate.
- Ask what could be problems or barriers in implementation of the activities, and how could these be solved?

Visit to Demonstration House

- Invite a group of participants to visit the Demonstration House directly after the village event. Invite only that part of the villagers, as was decided in the planning session. The other parts of the village should visit at the other Open House days, as was decided by the group.
- At the Demonstration House, observe the eaves from outdoors, then from indoors, and check the windows. Emphasize on small openings in the eaves.
- Discuss how the eaves can be made properly sealed so that no more small openings are visible from the inside of the house.

7. Monitoring and support

- During the month of September, the HI committee members and health animator should conduct monitoring of all houses in the village to check whether house improvement meets the quality standard.
- Explain to residents which part of the house needs further improvement, and how that is best done. However, residents cannot be forced to comply but have the right to refuse.
- The check-list in the Annex should be used. Records should be made by 'ticks' for houses in each category.
- Those houses that do not meet the standards should be visited again within 1 or 2 weeks' time.

ANNEX Check-list for monitoring house improvement

Date:

Indicate with one 'tick' per house

	**Observation**	**Yes**	**No**	**Action**
1	Observe from the outside of the house: Are there any gaps in the eaves on the four sides of the house that have not been closed?			If yes, put this house on the list for "basic house improvement", and discuss with the owner on how this could be achieved
	Total:			
2	Observe from the inside of the house: Can you see small openings (the size of a coin, or larger) or open slits along the eaves on any side of the house?			If yes, discuss with the owner on how to seal the small openings with clay or mud
	Total:			
3	Are there any windows that have not yet been properly closed with wire mesh or other materials? Check if the wire mesh is not hanging loose.			If yes, inform the owner on how to do the repairs or fix the wire mesh
	Total:			
4	Does the house have a door that cannot be properly shut?			If yes, inform the owner on how to repair the door or door frame
	Total:			

MANUAL FOR TRAINING WORKSHOPS FOR HI COMMITTEE MEMBERS AT CLUSTER-VILLAGE LEVEL (PHASE C)

Background

House improvement (HI) has been effective in many villages, but there remain two major **weaknesses**:

1. *Rusting*: At all sites, there is rusting of the wire gauze, often leading to **broken wire gauze**. Mosquitoes are able to enter the house through these openings. Apparently, the quality of wire gauze that was distributed by the project was poor. This problem will be solved by distribution of new wire gauze of better quality to replace wire gauze where rusting has caused perforations.

2. *Some houses not yet improved*: In some places, there are houses that have **not yet been improved** according to Phase-B standard. Phase-B standard means that no holes are visible in eaves and walls when standing on the inside of the house, and all windows are screened.

To address these weaknesses, a **1-day training workshop** will be given to all members of HI committees. Health animators, village chiefs and HSAs will also be invited to participate. The HI committees have not previously received training, other than through their participation in village workshops.

Objectives

1. **Increase knowledge, motivation and team building** among members of HI committees
2. Strengthen **maintenance and monitoring** of HI, particularly regarding rusted wire gauze
3. Engagement of HI committees in **problem identifying** and **problem solving** in house improvement

1. How do malaria mosquitoes enter a house, and how to stop them?

Objective

- Participants understand mosquito behaviour of house entry

Materials

- Visual material showing two lines of protection

Course content

16. Remind participants of the **role of mosquitoes** in the transmission of the malaria parasite: without the mosquito there cannot be malaria circulating within the community
17. Female mosquitoes search for humans when they are **hungry for a blood meal**.
18. How can they can trace humans? They can trace human from a distance with their excellent **sense of smell**.
19. Ask participants about the differences between **nuisance mosquitoes** (*Culex*) and **malaria mosquitoes** (*Anopheles*) (when do they bite, which are the noisy ones, which are dangerous?).
20. Ask: How do mosquitoes enter the house?
21. Most malaria mosquitoes enter the house **through the eaves**, which are the openings between the wall and the roof. Some also enter through windows or other openings.
22. In search of a house, the female mosquito travels low to the ground, and when arriving at the wall of a house, she **moves upwards, towards the eave** (or windows), where she enters the house.
23. Why does the mosquito move upwards? She follows the **odour that comes out of the eave** openings of the house. She recognizes the odours of people sleeping inside the house
24. Explain that bed nets are the first 'line of protection' and that house improvement is our **second 'line of protection'** against malaria transmission (even if the house is closed, bed nets should still be used!)
25. Show and explain the **visual**:

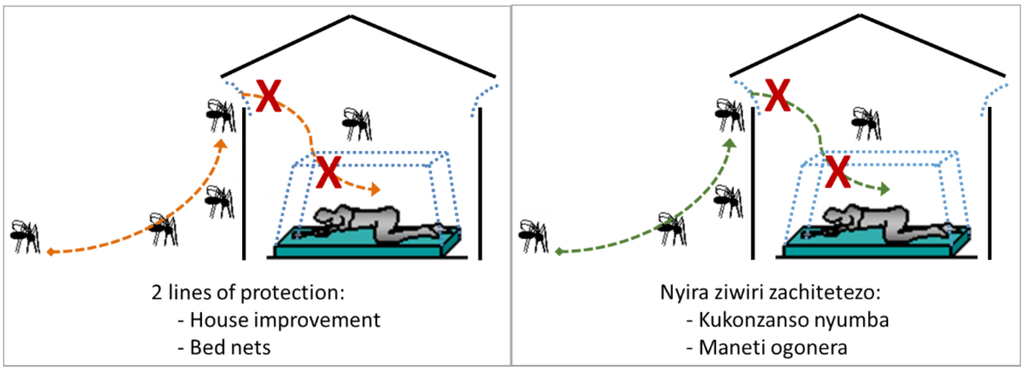

26. Ask: Why are **two lines of protection** (bed nets + house improvement) be better than one line of protection (Will it better protect us against malaria transmission)?
27. Our village was selected to join in the **first research in Malawi** on house improvement. The purpose of this research is to find out whether house improvement reduces the transmission of malaria. In 2018, if the result is positive, house improvement may come to many more villages.

Discussion

- Ask: Do you have any **experience or feedback** from our villages that house improvement does keep mosquitoes out?
- Is there sufficient **ventilation** during the night? If not, what are solutions to improve ventilation without letting mosquitoes enter?

2. Status on house improvement: where are the remaining gaps

Objective

- Participants understand the task ahead in their village

Course content

1. Remind participants that house improvement **started in July 2015** with brick making, followed by closing of the large gaps between the wall and roof (eave). This was followed by screening of windows with wire gauze. This was called Phase A of house improvement.
2. Unfortunately, after these operations had been completed, there were still many small **openings remaining** in the eaves. Malaria mosquitoes could easily enter the house through these openings. Therefore, **Phase-B** of house improvement was introduced in 2016.
3. What was done in Phase B? Phase B had a special focus on sealing all small openings in the eaves of houses, and using a **demonstration house** to show the villagers a closed house of good quality.
4. On a flip-chart paper, the facilitator **draws a map** of the two or three villages that participate in the cluster training.
5. Together with the participants, roughly **indicate on the map** the situation of house improvement:
    a. Indicate the location of **demonstration houses**
    b. Point out where **Phase A** is still lacking (where open eaves and open windows can be found).
    c. Point out where **Phase B** is still lacking (where smaller openings in the eaves and walls can be found)
    d. Point out where **maintenance** of house improvement is lagging behind (**rusting** of wire gauze; new openings in eaves)

6. It is important that most or all households in the village collaborate on house improvement. Only then, the effect of house improvement on reducing malaria can be measured.

Discussion

1. What if people **refuse** to improve their house (for example, for religious reasons) (Note: people are free to comply or not, and should never be forced if unwilling to improve their houses)

3. How to maintain and monitor house improvement

Objective

- Participants know the importance to **maintain and monitor** house improvement in their village.

Course content

7. Once a house has been closed, and windows screened, it will be necessary to do regular **maintenance**. Mosquito wire gauze can become rusty or broken, or windows can become poorly sealed. This would allow mosquitoes to enter.
8. Ask: what is the **condition of our own house**? Are there openings in the eaves, or is there rusting of wire gauze?
9. Ask committee members if they have a good idea about the condition of house improvement in the **entire village**?
10. Ask: What do we mean by **monitoring**?
11. Monitoring is the **regular** (weekly, or monthly) **inspection** of houses throughout the entire village, aiming to check whether maintenance or improvement is needed.
12. Ask: What **monitoring activities** have committee members been carrying out?
13. For any house that requires maintenance or improvement, **follow these steps**:
    a. In case the wire gauze covering windows and vent holes is rusting, check whether there are **perforations** in the wire gauze. Only when there are perforations, the wire gauze should be **replaced**. When there are no perforations, the wire gauze can still stay.
    b. **Measure** the height and width of the pieces of wire gauze that need replacement for that house, and note it down on a record sheet.
    c. In case the eaves have **small openings**, ask the head of household to make the repairs, explaining that it would keep mosquitoes out. Make a record, so that the house can be checked again after 1 week.
    d. In case the eaves have **not yet been closed**, or windows have not yet been screened, make arrangements to assist the household in these activities. Emphasize that an improved house will have fewer mosquitoes and therefore less malaria.

14. Committee members should **report** the results of their monitoring activities in committee meetings and to the health animator.
15. Ask: What to do when new wire gauze is needed in your village?
16. When wire gauze is required, the committee should **alert the health animator**. The health animator will then contact the EPO to bring more materials from his store within a few days' time. The new wire gauze is made of aluminum and should last longer than the product that was previously distributed. Aluminum does not rust.

Discussion

1. Why is it important to monitor houses in the village on a regular basis?

4. Drama on house improvement

Objective

- Participants recognize the common challenges in house improvement.
- Participants motivated to conduct drama in their own village.

Preparation

1. Well ahead of the training, contact the health animators from the cluster village. Ask them to prepare a drama (or song) about common challenges in house improvement. Examples are:
    a. Monitoring of houses in the village, talking to household heads
    b. Meeting of the house improvement committee

5. Roles and activities of HI committee

Objectives

- Participants aware of their own role within the HI committee.
- Participants aware of the role of the HI committee within the village.

Key points

1. The HI committee has a crucial role in all HI activities in the village.
2. Ask: What are the tasks of the HI committee?
3. The HI committee **mobilizes villagers** to improve their houses so that mosquitoes cannot enter.
4. In addition, the HI committee should **monitor the condition of houses** throughout the year.

5. Also, the HI committee members provide **feedback to the community** on all their activities during the village workshops on malaria.
6. With this range of activities, it is important that committee members **support each other**.
7. To accomplish these tasks in an orderly and transparent manner, it is useful for the committee to hold regular **committee meetings** to discuss all matters related to house improvement.
8. The health animator, HSAs and village head have the role of **advisor** to the committee.

Exercises for group work

1. Discuss **roles and responsibilities** of individual committee members.
2. What should be **agenda items** for a committee meeting?

6. Work planning

Objective

- Committee has planned its **timeline** of activities.

Key points

1. Ask: What is the **target** for house improvement in our village, or, where are the weaknesses that we have to tackle?
2. Ask: Which type of **activities** is the committee engaged in? (the facilitator should list the activities on a flip-chart paper in front of the group, and in the end, add any activity that was not mentioned from the list below).
    a. **Monitoring** of the condition of houses (including rusting of wire gauze)
    b. Conducting regular **committee meetings**
    c. **Assisting and motivating** the community in maintenance and house improvement
    d. Organizing a special **community event**
    e. Use of the **demonstration house** for visits from the community
    f. Reporting to the community in **village workshops** on malaria
    g. **Reporting to the animator** on the need for wire gauze
    h. Any **other** activity on house improvement
3. Divide the participants into groups: **one village per group,** each with their **chairperson** and **rapporteur**.
4. Ask each group to **prepare a work plan** for the committee for the next 6 months. They should list their activities, and indicate dates and responsible persons for each activity. (The activities and timeline should be realistic, so that they will actually be implemented; it is not useful to have an ambitious plan that will not be implemented).

Activity	Date	Responsible person

5. In the plenary discussion, request each group to **present** their plan.

Discussion

1. Is the work plan **feasible**, and can it achieve the targets?
2. How to **maintain and update** the work plan during the next 6 months?

7. Addressing the challenges in house improvement

Objective

- Committee members, chiefs, animators and HSAs discussing problems and solutions in house improvement.

Key points

1. Divide the participants into groups: **one village per group**
2. Ask each group to elect a **chairperson** and **rapporteur**.
3. Introduce the task for **group work**:
   a. What are the **main challenges** that obstruct or limit house improvement in our village? (for example, in relation to motivation, community mobilization, committee's role, village leadership)
   b. For each main challenge, discuss **possible solutions** (for example, by planning certain community activities, having regular committee meetings, improving monitoring, involvement of the chief and HSA).
4. If time allows, request 1 or 2 groups to **present** their outcomes.
