# Supplementary material for: Community-based malaria control in southern Malawi: a description of experimental interventions of community workshops, house improvement and larval source management
Source: Malar J. 2018 Jul 16;17:266. doi: 10.1186/s12936-018-2415-1 (PMC6048888; doi:10.1186/s12936-018-2415-1)
Supplement: Supplementary file 3 — Additional file 3. Training manual for health animators on larval source management for malaria prevention and control. [file 12936_2018_2415_MOESM3_ESM.pdf]

Additional file 3

TRAINING MANUAL
FOR HEALTH ANIMATORS ON

LARVAL SOURCE MANAGEMENT
FOR MALARIA PREVENTION
AND CONTROL

Majete Malaria Project

Majete Malaria Project – Community-based malaria control in the perimeter of Majete Wildlife Reserve

Contents_Toc507273604

PREFACE ... 3

INTRODUCTION ... 3

1. HOW TO ORGANIZE AND PLAN ... 5

2. BASICS ... 8

2.1 BREEDING OF MALARIA MOSQUITOES (LAUNCH MEETING) ... 8
2.2 COLLECTING AND RECOGNIZING MOSQUITO LARVAE ... 10
2.3 FILLING AND DRAINING OF BREEDING SITES ... 12

3. PLANNING ... 14

3.1 EXPLORING WHERE MOSQUITOES BREED IN OUR VILLAGE ... 14
3.2 COMMUNITY ORGANIZING TO REMOVE BREEDING SITES ... 15
3.3 KILLING MOSQUITO LARVAE WITH BTI ... 17

4. ACTION ... 19

4.1 COMMUNITY ACTION TO REDUCE MOSQUITO BREEDING-1 ... 19
4.2 COMMUNITY ACTION TO REDUCE MOSQUITO BREEDING-2 ... 20
4.3 COMMUNITY ACTION TO REDUCE MOSQUITO BREEDING-3 ... 22
4.4 COMMUNITY ACTION TO REDUCE MOSQUITO BREEDING-4 ... 24
4.5 COMMUNITY ACTION TO REDUCE MOSQUITO BREEDING-5 ... 25
4.6 COMMUNITY ACTION TO REDUCE MOSQUITO BREEDING-6 ... 27

ANNEX 1. PARTICIPANTS LIST (LARVAL SOURCE MANAGEMENT) ... 29

PREFACE

This manual has been developed by the Majete Malaria Project, as a collaborative effort between its project partners. Drafting of the document was by Henk van den Berg, with inputs provided by Rob McCann, Steve Gowelo, Alinune Kabaghe, Asante Kadama, Mackenzie Nkalapa, Zinenani Truwah, Monicah Mburu, Tumaini Malenga, Saidon Banda and Michele van Vugt.

This manual was first produced in November 2015. The manual is available in the English and Chichewa language. Funding for development of this manual was provided by Dioraphte Foundation, the Netherlands. Requests for permission to reproduce or translate this document – whether for sale or for non-commercial distribution – should be addressed to the programme manager (e-mail address: pmthrue@medcol.mw).

INTRODUCTION

This manual is a supplement to the malaria manual, by the Majete Malaria Project in collaboration with The Hunger Project. This manual is meant for use **only in those villages** that have been **selected for larval source management** during the community ballot box event. This intervention is part of a research trial to study the effectiveness of new vector control interventions on malaria.

Larval source management

Larval source management (or, larval control) is the management of mosquito breeding sites, aiming to reduce the number of mosquito larvae and pupae.

We can best protect ourselves against the bites of malaria mosquitoes, by sleeping under a net. Bed nets are very effective against malaria and should be used every night by all members of the household.

But we can also do something extra to **attack the malaria mosquitoes at their source**. This will be by managing the water bodies, puddles, ponds and swamps in which the malaria mosquitoes like to breed. Mosquitoes depend on water for their life-cycle. Without suitable water they cannot exist.

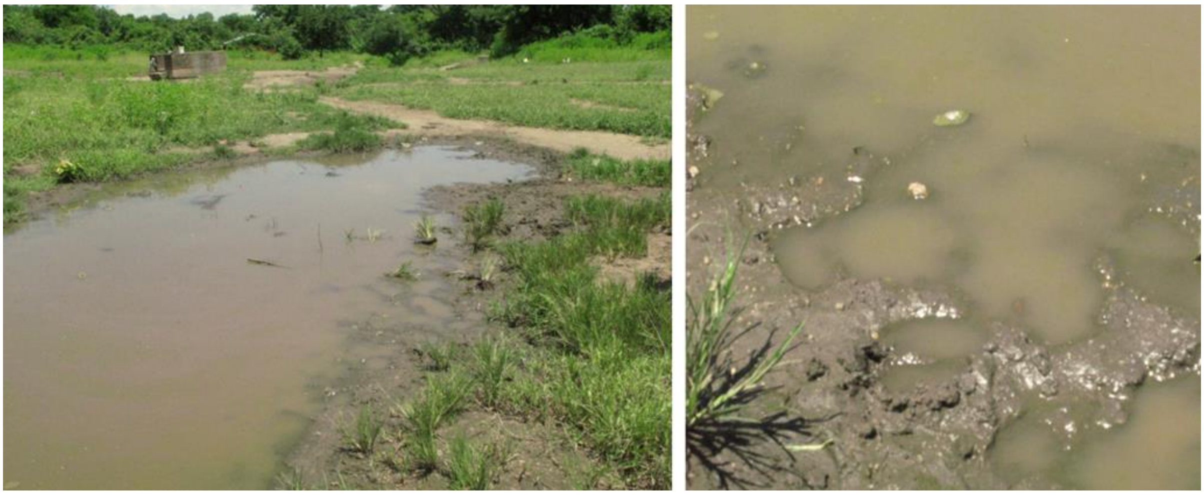

Example of breeding site of malaria mosquitoes in Majete (left); cattle hoof-print used for breeding by malaria mosquitoes (right).

The people in our villages are already aware of the importance of removing water bodies for mosquito control, because it has been discussed in the malaria manual. In this new manual, we will take this a step further by doing a **full-fledged campaign of** larval source management at village level.

Larval source management is an intensive strategy that can only work if the community actively participates throughout the year. During the **rainy season**, there can be a lot of malaria mosquitoes, and malaria usually peaks at this time. However, after the rainy season, and throughout the **dry season** there continue to be some malaria mosquitoes which manage to find some remaining water bodies in which to breed. These mosquitoes, even if they are few, can continue to circulate malaria in the community throughout the year. Therefore, we should manage the water bodies throughout the year, and attempt to break the transmission cycle of malaria.

Most water bodies could be filled or drained, so that water does not stay. In this way, any mosquito larvae initially available will die, and new larvae will not have water to develop at all. Some water bodies or swamps might be too big to be removed (drained or filled), or the water body might be important for use by the community. Malaria mosquitoes can continue to breed in these permanent water bodies even during the dry season. For these water bodies, there is another effective method available to kill mosquito larvae. This is the **spraying of BTI**, which is a safe, biological insecticide that kills only mosquito larvae.

Hence, larval source management will have **two components**:

1. Removal of breeding sites by the community
2. Spraying of BTI by a special committee

In this manual we will concentrate on the **first component only**, which is addressed at the entire community. However, it is important to know that the second component (on BTI) will also start in our village from April 2016. The two components will seamlessly work together to tackle mosquito breeding in all water bodies surrounding our village.

Roles and responsibilities

Health animators have the important task to mobilize the community to voluntarily implement larval source management through filling of draining of water bodies.

THP-Malawi is responsible for recruiting the health animators, managing the health animator team on the ground, and training and monitoring of the health animators in the conduction of village workshops.

The **Health and Education Unit** (HEU) of MoH is responsible for assisting the development of information, education and communication (IEC) materials, assisting the training of health animators, annual supervision of the health animator process and health promotion policy.

The **College of Medicine** is responsible for the technical aspects of training of the health animators and implementation.

Objective

The objective of this curriculum is to provide people living in selected villages with the **knowledge, tools and motivation** to implement larval source management throughout the year in the village and in the 400-meter zone surrounding the village.

This training is part of an intervention of a research trial, in which the purpose is to assess the impact of community-driven larval source management on reducing transmission of malaria parasites.

Use of this manual

This manual is used in the **training of health animators**. This same manual will also be used for **conducting the village workshops** on larval source management.

Chapter 1 of the manual addresses general aspects of organizing and planning the village workshops. **Chapters 2, 3 and 4** present the main topics for each village workshop.

1. HOW TO ORGANIZE AND PLAN

The village head is a **key person** in the activities on larval source management. The role of the village head is to give **support in coordination, organization and planning** of the community-level activities. Without this leadership support, it will be very difficult to implement a fully-fledged campaign on larval source management in the entire village. The health animator should always timely **inform** the village head and **request his participation** in the village workshops on larval source management.

The village workshops on larval source management will follow the same model as the ongoing village workshops on malaria prevention and control. The ongoing malaria workshops are every two weeks. The new village workshops on larval source management will be held **in between the ongoing malaria workshops**, once every month. This is illustrated in the diagram.

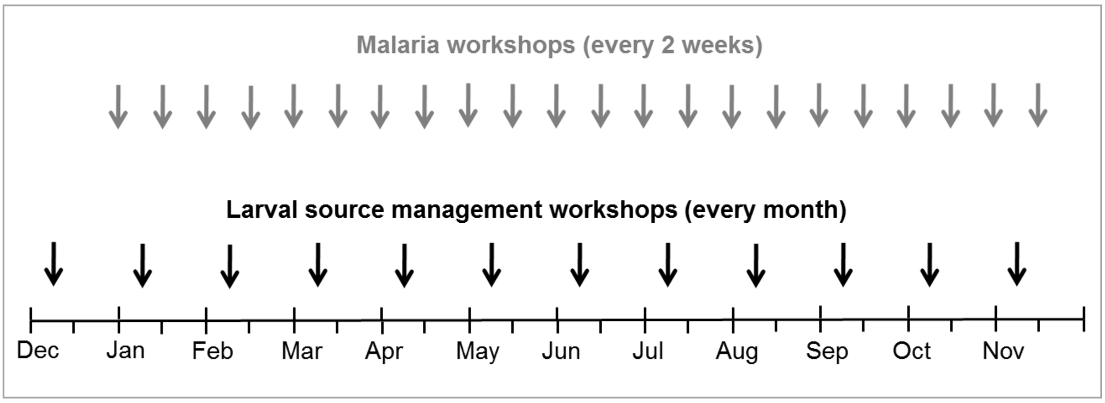

During the ongoing malaria workshops in the village, the health animator should make an **announcement** when the first workshop on larval source management will start. Villagers should be reminded of the community event, in which villages targeted with larval source management were selected through a lottery.

The health animator should carefully plan the dates for each topic of the course content. A provisional plan is presented in Table 1.

The workshops are divided into several categories, or ‘blocks’:

1. The first is on the **Basics** (December 2015-February 2016). The aim is to give villagers the basic understanding on larval source management, while at the same time, encouraging them to start practicing it.
2. The second block is related to **Planning** (March-May 2016). This will help the village to organize and plan together on larval source management.
3. Finally, the third block is on the **Action** (June-November 2016).

Therefore, villagers are encouraged to practice larval source management from the start (December 2015). When the workshops focus on Planning (March-May 2016) and Action (June-November 2016), the practice on larval source management will become more coordinated by the village as a whole.

It is important to stick to the schedule with monthly intervals, for continuity of the field-level activities, and because the content of sessions is adapted to the seasons.

Table 1. Plan for the course content of village workshops on larval source management (from December 2015). Chapter numbers are indicated for easy reference.

Block	Workshop	Time	Course content	Manual chapter
Basics	1	Dec'15	Breeding of malaria mosquitoes (launch meeting)	2.1
	2	Jan'16	Collecting and recognizing mosquito larvae	2.2
	3	Feb'16	Draining and filling of breeding sites	2.3
Planning	4	Mar'16	Exploring where mosquitoes breed in the village	3.1
	5	Apr'16	Community organizing to remove breeding sites	3.2
	6	May'16	Killing mosquito larvae with BTI	3.3
Action	7	Jun'16	Community action to reduce mosquito breeding-1	4.1
	8	Jul'16	Community action to reduce mosquito breeding-2	4.2
	9	Aug'16	Community action to reduce mosquito breeding-3	4.3
	10	Sep'16	Community action to reduce mosquito breeding-4	4.4
	11	Oct'16	Community action to reduce mosquito breeding-5	4.5
	12	Nov'16	Community action to reduce mosquito breeding-6	4.6

The **workshop agenda** is quite similar to that of the malaria workshops. The only difference is that there is no agenda item on 'self-monitoring of malaria' (Table 2). The agenda items have been explained in detail in the malaria manual, and will not be repeated here.

Table 2. Agenda items for community workshops on larval source management; total duration approx. 2 hr.

Nr.	Workshop agenda item	Min	Notes	Self monitoring
1	Opening remarks and workshop agenda	10		Has the agenda been explained?
2	Introducing participants	10	Secretary completes participant record sheet	Are new participants introduced?
3	Summary of previous workshop	5		Is summary of previous session given?
4	Course content	45	See course content per session*	Are the key points presented clearly?
5	Group discussion	30	See questions related to course content*	Does a lively group discussion take place?
6	Plan for next workshop, and closing	5	Create expectation and encourage participation	Is next session's topic announced?

* See course content according to the schedule in table 1

The participant list should be completed in each village workshop on larval source management. This will assist the health animator in identifying households that do not participate. Participant lists are also needed for monitoring purposes. The participants list is kept by the secretary. An example of a participant list is provided in **Annex 1**.

2. BASICS

2.1 Breeding of malaria mosquitoes (launch meeting)

Objective

- Participants understand the meaning of larval source management.
- Establishment of a committee for larval source management.
- Launch of the campaign on larval source management.

Materials and preparation

- This workshop should serve as the **launch** of the campaign on larval source management in the village. Therefore, the date of the workshop should be **announced** to the villagers ahead of time, and a special agenda item (such as a drama on malaria mosquitoes, or a festive activity) should be added to attract a large number of participants.
- Visual-1

Course content

1. Ask: Where do mosquitoes come from?
2. Remind participants that **mosquitoes need standing water** for laying their eggs and producing offspring. Without water there can be no mosquitoes. Therefore, water bodies are the **source** of malaria mosquitoes.
3. Show **Visual-1 with the mosquito life-cycle**. The female mosquito lays her eggs in the water. The emerging larvae live and swim in the water. When the larva is fully-grown, it will become a pupa. After some days, the pupa breaks open and the mosquito with wings comes out.

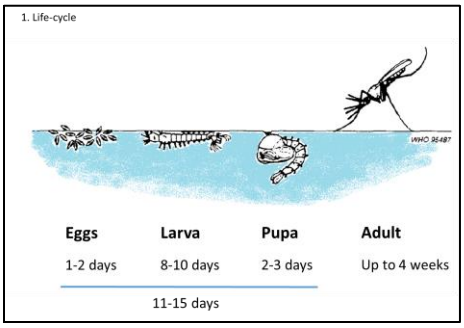

Source: modified from WHO

4. Ask: What happens to the mosquito larvae and pupae when the **water disappears**? (all mosquito larvae will die)
5. Explain that **Larval Source Management** is the control of mosquitoes right at their source (where they are coming from), which are the breeding sites. The aim is to reduce the number of mosquito larvae and pupae developing in water bodies.
6. Remind participants about the **lottery event** in which this village was selected for larval source management. Ask who were not aware of this.

7. Some villages have been selected for larval control and some other villages will be kept without larval control. This allows us to **compare the two villages** – those with and those without larval control.
8. There are **three methods** for larval source management: draining of water bodies, filling of water bodies, or the use of insecticides to treat water bodies.
9. **Draining and filling** are the best methods, because they simply remove the water so that mosquitoes can no longer breed there.
10. Some **larger water bodies**, such as large swamps or floodplain pools near the river, cannot easily be drained or filled. Malaria mosquitoes can continue to breed in these water bodies unless we kill them with insecticide spraying.
11. **BTI is an insecticide** that will be used in this village from April 2016 onwards, after the rainy season. BTI will not be used during the rainy season because the rains can wash away the insecticide.
12. Larval source management is a method that is not yet being used in Malawi, but we need to know how effective it is in reducing malaria mosquitoes and reducing the malaria parasite.
13. In our village we will **start a campaign** to stop the breeding of malaria mosquitoes by using these three methods of larval source management (draining, filling, and BTI). This campaign will **last for two years**.
14. Larval source management is a **community effort**: it cannot be effective when carried out by only a few persons, but the whole community should actively participate.
15. The overall **leadership** is with the village head – his role is to oversee the larval source management in the village. The health animator will be the organizer of the larval source management activities.
16. To assist the village head and health animator, there should be a special **committee** for larval source management. These are a number of energetic persons who are willing to commit time and energy to improve the malaria situation in the village.
17. The **tasks** of the committee would be:
    a. To **mobilise villagers** to remove standing water around their homestead, out to 400 m from the last house in the village.
    b. To **monitor the situation** on mosquito breeding throughout the year.
18. Ask for **nominees** for the committee on larval source management. Ask participants to make the selection in a democratic or other locally acceptable, fair manner. The workshop secretary should note down their names.
19. The village head and Health Surveillance Assistant (HSA) will be asked to serve as **advisers** of the committee.

Group discussion

1. What do villagers expect from the committee, the HSA and village head?
2. What do committee members, HSA and village head expect should be the role of the community in larval source management?

2.2 Collecting and recognizing mosquito larvae

Objective

- Participants able to distinguish the larvae of malaria mosquitoes from those of nuisance mosquitoes.
- Participants able to collect mosquito larvae.

Materials

- Visual-2 and Visual-3
- Light-coloured plastic cup and/or a soup spoon
- Two transparent jars with live mosquito larvae in water: one with malaria mosquitoes (Anopheles) and one with nuisance mosquitoes (Culex)
- Ask the committee members to prepare for a field visit after the village workshop

Course content

1. Remind participants of the **role of mosquitoes** in the transmission of the malaria parasite: If we remove malaria mosquitoes we will suffer less from malaria.
2. Ask **who has observed mosquito larvae**, how did they look, and how could we know whether they are malaria mosquitoes.
3. Show **Visual-2** with the larva of a malaria mosquito (Anopheles). The larvae are minute. The older larvae are easily visible if we know what to look for. Young larvae are very small. The larvae swim just below the water surface, with their body **in line with the surface**.

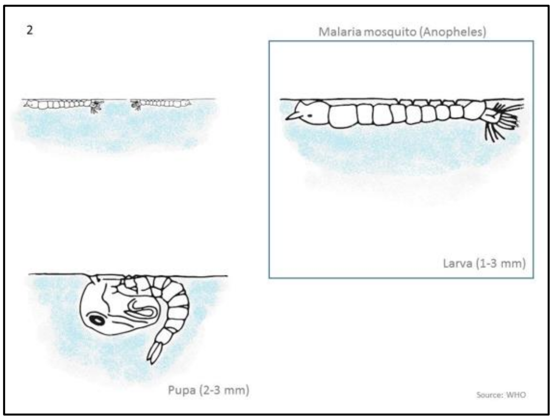

Source: modified from WHO

4. Now show **Visual-3** with the larva of a nuisance mosquito (Culex). Point out the differences with malaria mosquitoes: Larvae of Culex hang straight in the water, and have a long breathing tube pointing at the water surface. Culex larvae waggle more actively in the water than Anopheles larvae.

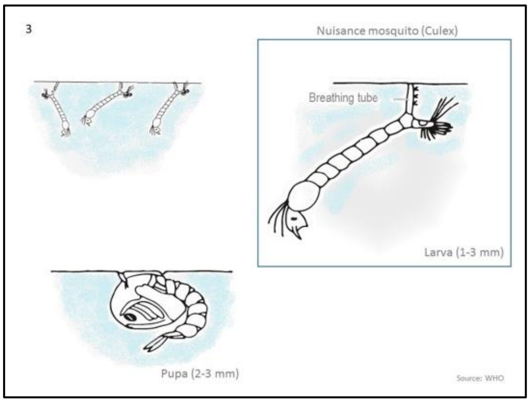

Source: modified from WHO

5. Show the **two jars with live specimens** of Anopheles and Culex larvae. Ask participants to pass them around, spotting the differences in how they look and how they behave.
6. Ask participants if they have seen any of these two types of larvae (Anopheles or Culex) in standing water.
7. Water bodies where we find Anopheles larvae are a **risk for malaria**, because the larvae become malaria mosquitoes and can transmit malaria parasites.
8. Water bodies in which only Culex larvae are breeding are not a risk for malaria, but can cause a **nuisance problem** (mosquito biting at night).
9. Anyone can try to collect mosquito larvae by using a **white plastic cup or a soup spoon**. Ask participants if they have these items at home that they could use for collecting larvae (show example of cup and spoon).
10. **Repeated practice** is necessary for us to recognize and understand the mosquito larvae – how they look and how they behave. Only then will we be able to distinguish Anopheles and Culex larvae.
11. Now, request the **committee members to report** on their initial activities since the establishment of the committee last month.

Group discussion

- Why is it important to **recognize** the larvae from malaria mosquitoes and nuisance mosquitoes?
- Are there water bodies in our own surroundings where we can **try to collect** mosquito larvae?

Field collection

- After the village workshop, ask the committee members to guide the participants to a nearby larval breeding site not far from the meeting location. Let participants get a cup or spoon from home, if possible. Practice the collection method with the participants, collect larvae and pupae, and try to identify the collected specimens together.

2.3 Filling and draining of breeding sites

Objective

- Participants understand the correct methods of filling and draining

Materials

- Visual-4 and Visual-5

Course content

1. Remind participants that when malaria mosquitoes are present in our village there are water bodies in which they can breed. Without water there will be no mosquitoes.
2. Malaria mosquitoes like to breed in many **different types of water bodies**, for example:
    - Small puddles
    - Cattle hoof prints
    - Borrow pits (left behind after extracting clay for producing bricks)
    - Ponds
    - Edges of swamps
    - River floodplain pools
    - Standing water in streambeds and drainage channels
3. Malaria mosquitoes prefer to breed **close to people's houses**, so that they don't have to fly far to find a human to feed on.
4. But if necessary, mosquitoes will fly **over 400 meter** to reach a house with humans. This means that mosquitoes breeding in river banks and swamps just outside the village (less than 15 minutes walk) may be able to find and reach our houses.
5. Ask: What can we do to stop mosquito breeding in our village?
6. There are two methods that we can use to remove the water bodies. These are: filling and draining.
7. Show **Visual-4** about Filling.

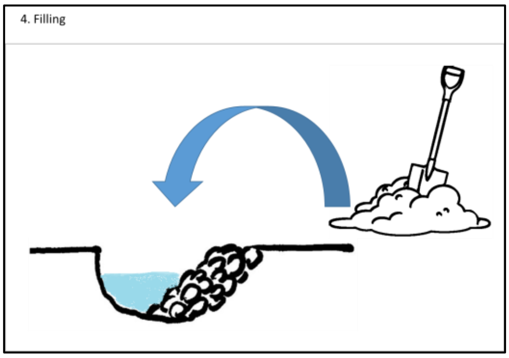

8. Small puddles, borrow pits and some water holes can be **filled with soil**, **stones, rubble** to permanently close them. This can be a long-term solution to prevent mosquito breeding in the future.

9. **Waste materials** could also be used for filling pits or holes, but should be compacted and topped up with clean soil to ensure that the waste will not come to the surface after some time.
10. Special care must be taken that by obtaining filling material we do **not create new depressions** in the soil that could accumulate water!
11. Ask: At this time are there any water bodies that need to be filled?
12. Ask: What if the water body is too large to be filled?
13. Some water bodies can be **drained by digging a drainage canal** where there is a gradient toward lower ground. Care must be taken that drained water will not create a new water accumulation lower down.
14. Show and explain the **Visual-5** about Draining:

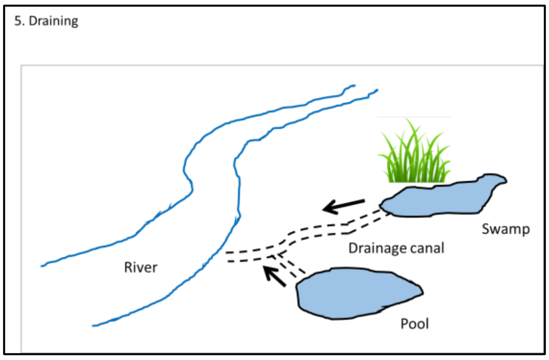

15. Ask participants whether they know water bodies around their village that could be targeted for drainage and how they would do it.
16. **Land levelling** is a combination of draining and filling, aiming to prevent accumulations of water in the future.
17. There may be some water bodies around the village that **cannot be filled or drained**. Here, the best option is for us to **do nothing.** Examples are:
    - Watering holes needed for cattle
    - Irrigation in agricultural crops
    - Large swamps
    - Temporary floodplain pools near the river
18. Ask participants if there are such examples around their village.

There is one more method to control mosquito breeding. If malaria mosquitoes are found in water bodies, they could be sprayed with a product called **BTI**. This is an insecticide that kills only mosquitoes but no other animals. The program on BTI will start in April in our village, and will not be discussed before that time.

19. Now, request the **committee members to report** on the current situation of mosquito breeding sites in the village.

Group discussion

- How do we decide whether to **fill** or **drain** a water body, or whether to **do nothing**?
- Are some water bodies needed for **livestock** or for **irrigation** of crops? If yes, how can we reduce mosquito breeding in these water bodies?

3. PLANNING

3.1 Exploring where mosquitoes breed in our village

Objective

- Participants conduct mapping of breeding sites in their village.
- Participants know how to explore the village environment for breeding sites.

Materials

- Visual-6
- Flip-chart paper with markers (if available)

Course content

1. Remind participants that the malaria mosquitoes that bite us at night have emerged from pupae that were living in **water bodies** from within the village environment. Without these water bodies there would be no mosquitoes or malaria.
2. **Bed nets** are the most effective protection against mosquito bites and malaria. Bed nets should be used by all family members throughout the year.
3. Ask participants whether they remember how far a malaria mosquito can fly.
4. Mosquitoes can **fly 400 meters**, and even further, which means that those breeding in swamps at 10 minutes walk distance from the village can still reach our houses (although many might not reach our houses). Those mosquitoes breeding closer to our houses are **more likely to find us** at night, because they are already inside the village.
5. Show **Visual-6**, pointing out the 400-meter zone around the village.

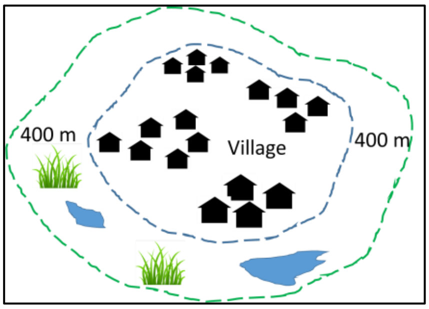

6. Larval source management is a **community effort**, and all households of the village should join.
7. Ask: **Why is it important that all households join** in larval source management? Suppose that breeding sites are removed, but in one pocket of the village mosquitoes continue to breed. What would be the result? (this could keep malaria transmission going in the village)

8. Ask the committee members to assist in drawing a large map of the village, in the soil or on paper - for everyone to see.
    - Indicate the 'village proper' where houses are located
    - Draw a zone around the village of 400 meters wide (this is the 'backyard' of our village, where mosquitoes can breed and fly to reach our houses)
    - Indicate where are agricultural crops, and waterways
    - Indicate where are water bodies (pools, swamps, etc.)
    - Indicate where Anopheles larvae have been found
    - Indicate where Culex larvae have been found
    - Indicate on the map where we do not yet know about the presence of mosquito larvae
9. Next, ask the committee members to summarize the **current situation** of mosquito breeding in the village. Has the situation improved or worsened?

Group discussion

- How can we ensure that we explore mosquito breeding in all parts of the village (including swamps and floodplain pools)?
- How regularly should the exploration take place (every week; every month)?
- How are the rains affecting the breeding of mosquitoes (do rains increase breeding; do rains wash away mosquito larvae)?

Field visit

- After the village workshop, ask the committee members to guide the participants for a **village walk**. Several mosquito breeding sites in the village and around the village are visited, and larvae collected and identified. The feasibility of filling and draining is discussed.

3.2 Community organizing to remove breeding sites

Objective

- Participants prepare a plan for village-wide implementation of larval source management
- Participants agree on roles in implementing the plan

Materials and preparation

- Visual-6
- One or two days before the village workshop, meet with the village head and committee

Course content

1. Remind participants that malaria **parasites are circulating** in the community of our village. These parasites are spread from person-to-person through the bite of a malaria mosquito. If we remove the mosquitoes, malaria will disappear.
2. Ask: How can we **remove** the mosquitoes at their source? Where is the source (where are the breeding sites)?
3. Explain that last month we learnt how to explore the mosquito breeding sites in our village. We also learnt how to remove a breeding site by filling or draining. Today we will make an **action plan** for removing the breeding sites (make sure the secretary makes notes of what will be decided today).
4. Explain what is meant by a **community effort**. Larval source management aim to reduce the malaria burden in the **entire village**. There is much work ahead, and therefore, everyone's participation is needed.
5. Show **Visual-6**, pointing out the 400-meter zone around the village.

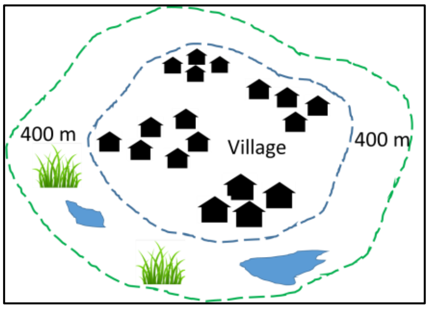

6. The **committee** on larval source management should mobilize and coordinate the campaign on filling and draining of breeding sites. Overall **leadership** is with the village head – his role is to oversee the larval source management in the village.
7. Ask the committee members to assist in drawing a large **map of the village** (just as we did last month), in the soil or on paper - for everyone to see.
    - Indicate where are houses, and indicate the **zone of 400 meters around the village** (show areas of agricultural crops, and waterways)
    - Indicate where are water bodies, and point out where <u>Anopheles</u> larvae have so far been found
8. **Identify the water bodies** to be filled and those to be drained.
9. Also identify the water bodies that **cannot** be filled or drained.
10. Determine **how many persons** are needed to help with filling or draining. Assign roles to households according to the locations of the water bodies.
11. Make an inventory of the **tools needed** for the activities (for example: spades, pick-axes), and the tools available within the village. Discuss the best possible use of available tools.
12. What would be the **best time** for the actions? Should we wait a bit for the current rain showers to cease or can the action be started? How much time

would be needed for the filling and draining action? How regularly should the actions be **repeated**?

13. Give the floor to the committee on larval source management. Ask them how these actions will be **coordinated**. Who will **keep records** on what has been accomplished; when and where?
14. Finally, summarize what was agreed for the action plan on larval source management:
    a. Which breeding sites to be filled and drained
    b. Persons to be involved
    c. Use of available tools
    d. Timing of action
    e. Coordination and record keeping

Group discussion

1. Discuss any remaining issues related to the action plan on larval source management.

3.3 Killing mosquito larvae with BTI

Objective

- Participants aware of the BTI program in their village.
- Participants understanding the role of BTI to complement the filling and draining of breeding sites.

Materials

- Knap-sack sprayer with protective equipment
- A sample of BTI product

Course content

1. Remind participants about the **campaign** for larval source management: We want to remove all breeding sites of malaria mosquitoes, so that there will be less malaria in the village.
2. Briefly repeat the summary of the **action plan** that was developed last month.
3. Ask the committee members to give an **update** on the implementation of the action plan, and to report on the **current situation** of mosquito breeding in the village. Has the situation improved or worsened?
4. Ask one or two of the **participants** to explain how they have helped with the filling and draining of breeding sites.
5. Ask about the breeding sites that **cannot be filled or drained**. Where are they located and how large are they?

6. Breeding sites that cannot be filled or drained can continue to be a source of malaria mosquitoes, when larvae breed in them. But there is an **additional method** that we will use for those breeding sites.
7. BTI is an insecticide that will be **sprayed by committee members** that have received special training.
8. BTI is an insecticide that is different from those insecticides used in agriculture (for example, for spraying cotton against insect pests). This insecticide BTI **kills only mosquito larvae.** When normally used, BTI is harmless to other insects, fish and domestic animals and humans.
9. BTI is only active for a **short period**, and has to be applied every few weeks to be effective.
10. BTI is an **expensive** product, and its use should be limited to only those breeding sites where other methods (filling, draining) are not applicable.
11. For this reason, the spraying of BTI should be **closely coordinated** with the action plan for filling and draining of breeding sites. By filling and draining more and more breeding sites, the spraying of BTI can become more concentrated and more effective.
12. Ask the committee members who have been trained on BTI to **show a knapsack sprayer** and BTI product. Ask them to explain what they have learnt in the training, what protective equipment and protective measures they should take to avoid exposure to the insecticide. Ask them how BTI will help in removing malaria mosquitoes from the village.

Group discussion

- Now that we have a BTI committee with spray operations, discuss why we still need to **continue with filling and draining** of breeding sites.
- If the BTI committee finds breeding **sites that could easily be filled or drained**, what should they do?
- How can the BTI committee **reduce the use of the costly BTI product**?

Field visit

- After the village workshop, ask the committee members to guide the participants for a **demonstration of BTI spraying**, using the protective equipment. Select a suitable water body that cannot be filled or drained, observe whether there are any Anopheles larvae, and then demonstrate how spraying is done (without wasting much BTI product). Make sure that the participants are at a safe distance from the sprayer.

4. ACTION

4.1 Community action to reduce mosquito breeding-1

Objective

- Interaction between BTI team and other villagers about the larval source management activities
- Participants stimulated to plan and implement further activities.

Materials

- None

Course content

1. Remind participants about the **role of mosquitoes** in transmission of malaria parasites in our village.
2. Also, remind participants about the **action plan** on larval source management, aiming to stop the breeding of malaria mosquitoes in and around our village.
3. Inquire from the participants whether there have been any **malaria cases** during the past month. If there are any cases, this is a sign that malaria mosquitoes are still present in our village.
4. Also ask the **HSA** to give report on the number of confirmed malaria cases this past month.
5. Ask: How will the dry season influence malaria mosquitoes?
6. Explain that, with the dry season approaching, there will be fewer and **fewer water bodies** in which malaria mosquitoes can breed. It is important that we do not relax our campaign for larval source management but continue throughout the dry season.
7. In the dry season, the malaria mosquitoes are in a **weak position**, with only few breeding sites. If we manage to find these breeding sites we break their life-cycle and reduce their numbers from the village.
8. Ask the committee members to take the floor and report on their monitoring activities of the **current situation**:
    a. Are breeding sites present in or around the village?
    b. Are Anopheles larvae found in those breeding sites?
    c. Are there any other possible sites within the village where malaria mosquitoes could be coming from? If yes, how could they be monitored and controlled?

    d. Does the committee need any help from villagers (or youth) in monitoring of Anopheles larvae? If yes, how could this be arranged?

9. Ask the committee members to report on their **larval source management** activities.
    a. How many breeding sites have been sprayed with BTI?
    b. When and how often was spraying done?
    c. Was the BTI effective at killing mosquito larvae?
    d. Are any of these water bodies suitable for filling or draining? If yes, discuss how this could be done.
    e. Are there any other possible sites within or around the village where malaria mosquitoes could be coming from? If yes, how could they be controlled?
10. Ask one or two villagers (not committee members) to describe how they have contributed to larval source management over the past month, and what challenges they faced.

Group discussion

- Discuss whether the situation of mosquito breeding is **improving or worsening**. On what basis do we make our conclusion?
- Are there localities in or around the village in which the community should help in **filling or draining** of breeding sites?
- What **challenges** are faced in larval source management, and how can these be solved?
- Ask the participants to evaluate whether the BTI committee is **performing as expected**, or are any measures needed to strengthen the committee and their actions?

4.2 Community action to reduce mosquito breeding-2

Objective

- Interaction between BTI team and other villagers about the larval source management activities
- Participants stimulated to plan and implement further activities.

Materials

- Visual-2 and Visual-3

Course content

1. Remind participants about the campaign on larval source management, aiming to remove the breeding sites of malaria mosquitoes.

2. Ask: Why are we removing water bodies? Why do we want to kill malaria mosquitoes? What is the aim?
3. Ask **who has observed mosquito larvae** from close-by, and who is able to distinguish the larvae from malaria mosquitoes (Anopheles) and those of nuisance mosquitoes (Culex).
4. Remind participants about the differences with malaria mosquitoes by showing Visual-2 and Visual-3.
5. Anopheles larvae swim just below the water surface, with their body **in line with the surface**. Larvae of Culex stand straight in the water, and have a long breathing tube pointing at the water surface. Culex larvae waggle more actively in the water than Anopheles larvae.

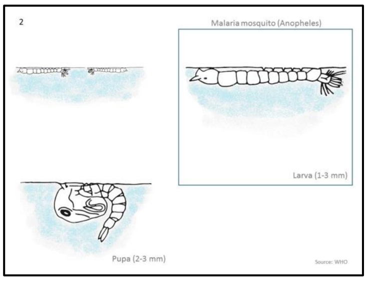

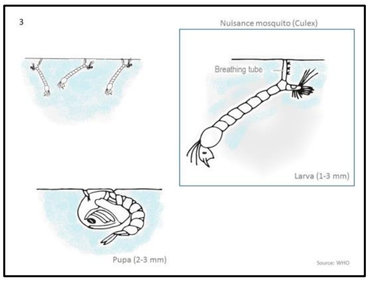

6. Inquire from the participants whether there have been any **malaria cases** during the past month. If there are any cases, this is a sign that malaria mosquitoes are still present in our village.
7. Also ask the **HSA** to give report on the number of confirmed malaria cases this past month.
8. Ask the committee members to take the floor and report on their monitoring activities of the **current situation**:
    a. Are breeding sites present in or around the village?
    b. Are Anopheles larvae found in those breeding sites?
    c. Are there any other possible sites within the village where malaria mosquitoes could be coming from? If yes, how could they be monitored and controlled?
9. Ask the committee members to report on their **larval source management** activities of the past month.
    a. How many breeding sites have been sprayed with BTI?
    b. When and how often was spraying done?
    c. Was the BTI effective at killing mosquito larvae?
    d. Are any of these water bodies suitable for filling or draining? If yes, discuss how this could be done.
    e. Are there any other possible sites within or around the village where malaria mosquitoes could be coming from? If yes, how could they be controlled?

Group discussion

- Does the committee need any help from villagers (or youth) in monitoring of Anopheles larvae? If yes, how could this be arranged?
- What **challenges** are faced in larval source management, and how can these be solved?
- Ask the participants to evaluate whether the BTI committee is **performing as expected**, or are any measures needed to strengthen the committee and their actions?
- Discuss whether the situation of mosquito breeding is **improving or worsening**. On what basis do we make our conclusion?

4.3 Community action to reduce mosquito breeding-3

Objective

- Interaction between BTI team and other villagers about the larval source management activities
- Participants stimulated to plan and implement further activities.

Materials

- Visual-4 and Visual-5

Course content

1. Remind participants that malaria mosquitoes can only flourish when there are suitable water bodies in which they can **breed**, and people on which their can **feed** (blood-meals). If we remove all water bodies, there will be no more mosquitoes.
2. Ask: What is the relationship between **water bodies** and **malaria**?
3. Request the village head, or other senior person, to explain the **action plan** on larval source management, which aims to stop breeding of malaria mosquitoes.
4. Explain that in the dry season there are only few **water bodies** in which malaria mosquitoes can breed. If we manage to find these breeding sites we break their life-cycle and reduce their numbers from the village.
5. Show **Visual-4** and **Visual-5** to remind participants about Filling and Draining.

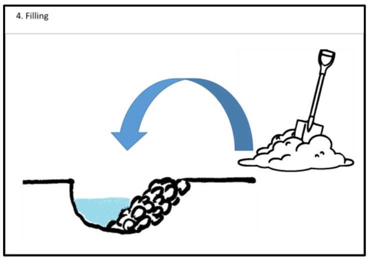

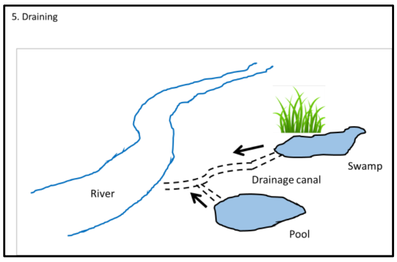

6. Small puddles, borrow pits and some water holes can be **filled with soil**, **stones, rubble** to permanently close them.
7. Some water bodies can be **drained by digging a drainage canal** where there is a gradient toward lower ground.
8. Ask one or two villagers (not committee members) to describe how they have contributed to larval source management over the past month, and what challenges they faced.
9. Ask: Which water bodies in or around our village cannot be filled or drained? How could we kill malaria mosquitoes breeding in these water bodies?
10. Ask the committee members to take the floor and report on their monitoring activities of the **current situation**:
    a. Are breeding sites present in or around the village?
    b. Are <u>Anopheles</u> larvae found in those breeding sites?
    c. Does the committee need any help from villagers (or youth) in monitoring of Anopheles larvae? If yes, how could this be arranged?
11. Ask the committee members to report on their **larval source management** activities.
    a. How many breeding sites have been sprayed with BTI?
    b. When and how often was spraying done?
    c. Was the BTI effective at killing mosquito larvae?
    d. Are any of these water bodies suitable for filling or draining? If yes, discuss how this could be done.
    e. Are there any other possible sites within or around the village where malaria mosquitoes could be coming from? If yes, how could they be controlled?
12. Inquire from the participants whether there have been any **malaria cases** during the past month. If there are any cases, this is a sign that malaria mosquitoes are still present in our village.
13. Also ask the **HSA** to give report on the number of confirmed malaria cases this past month.

Group discussion

- Are there localities in or around the village in which the community should help in **filling or draining** of breeding sites?
- Discuss whether the situation of mosquito breeding is **improving or worsening**. On what basis do we make our conclusion?
- Ask the participants to evaluate whether the BTI committee is **performing as expected**, or are any measures needed to strengthen the committee and their actions?

4.4 Community action to reduce mosquito breeding-4

Objective

- Interaction between BTI team and other villagers about the larval source management activities
- Participants stimulated to plan and implement further activities.

Materials

- Visual-6

Course content

1. Ask: How can we protect ourselves against malaria mosquitoes?
2. Bed nets are the number 1 protection method, and should be used by all household members and throughout the rainy and dry seasons.
3. We can also **attack** malaria mosquitoes **at their source**, by removing the water bodies in which they breed. This is why we have a campaign on larval source management in the village.
4. Now that it is **dry season**, there are only very few water bodies in which malaria mosquitoes can breed. These may be swamps or floodplain pools that are at some distance from the village.
5. If these breeding sites are less than **400 meters** from the edge of the village, mosquitoes will still reach our houses.
6. Show **Visual-6**, pointing out the 400-meter zone around the village. Larval source management should be conducted in and around the village.

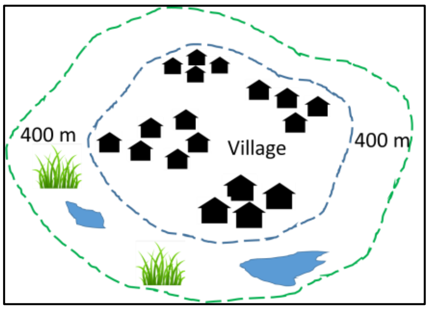

7. Ask whether there have been any **malaria cases** during the past month. If there are any cases, this is a sign that malaria mosquitoes are still present in our village.
8. Also ask the **HSA** to give report on the number of confirmed malaria cases this past month.
9. Ask the committee members to take the floor and report on their monitoring activities of the **current situation**:
    a. Are breeding sites present in or around the village?
    b. Are <u>Anopheles</u> larvae found in those breeding sites?

c. Are there any other possible sites within the village where malaria mosquitoes could be coming from? If yes, how could they be monitored and controlled?

d. Does the committee need any help from villagers (or youth) in monitoring of Anopheles larvae? If yes, how could this be arranged?

10. Ask the committee members to report on their **larval source management** activities.

    a. How many breeding sites have been sprayed with BTI?

    b. When and how often was spraying done?

    c. Was the BTI effective at killing mosquito larvae?

    d. Are any of these water bodies suitable for filling or draining? If yes, discuss how this could be done.

11. Ask one or two villagers (not committee members) to describe how they have contributed to larval source management over the past month, and what challenges they faced.

Group discussion

- Discuss whether the situation of mosquito breeding is **improving or worsening**. On what basis do we make our conclusion?
- Are there localities in or around the village in which the community should help in **filling or draining** of breeding sites?
- Is the **BTI committee** is doing a good job at BTI spraying? Do they give adequate information to the other villagers?

4.5 Community action to reduce mosquito breeding-5

Objective

- Interaction between BTI team and other villagers about the larval source management activities
- Participants stimulated to plan and implement further activities.

Materials

- None

Course content

1. Remind participants about the **role of mosquitoes** in transmission of malaria parasites in our village.
2. Ask: How can we protect ourselves against mosquito bites.

3. **Bed nets** are the best method for protecting against malaria. Bed nets should be used by all household members, both in the dry season, and when the rains start.
4. Besides bed nets, our village has a campaign on larval source management, to attack the mosquitoes **at their source**, which is: at their breeding sites in water bodies.
5. Towards the **end of the dry season** malaria mosquitoes are in a weak position, because they can find only very few water bodies in which to breed. If we manage to find and control these last few water bodies, we can drastically reduce the number of malaria mosquitoes. This could break the transmission cycle of malaria parasites.
6. When it **starts raining**, new water bodies will emerge. These could be depressions in the soil that accumulate rain water. If water remains in these pools for 2 weeks or more, mosquitoes can breed in them.
7. Inquire from the participants whether there have been any **malaria cases** during the past month. Also ask the **HSA** to give report on the number of confirmed malaria cases this past month.
8. If there are any malaria cases, this is a sign that malaria mosquitoes are **still present** in our village. There must have been a water body from which they emerged.
9. Ask the committee members to take the floor and report on their monitoring activities of the **current situation**:
    a. Are breeding sites present in or around the village?
    b. Are Anopheles larvae found in those breeding sites?
    c. Does the committee need any help from villagers (or youth) in monitoring of Anopheles larvae? If yes, how could this be arranged?
10. Ask the committee members to report on their **larval source management** activities.
    a. How many breeding sites have been sprayed with BTI?
    b. When and how often was spraying done?
    c. Was the BTI effective at killing mosquito larvae?
    d. Are any of these water bodies suitable for filling or draining? If yes, discuss how this could be done.
    e. Are there any other possible sites within or around the village where malaria mosquitoes could be coming from? If yes, how could they be controlled?
11. Ask one or two villagers (not committee members) to describe how they have contributed to larval source management over the past month, and what challenges they faced.

Group discussion

- Are there localities in or around the village in which the community should help in **filling or draining** of breeding sites?
- At this moment in time, what **challenges** do we face in larval source management (BTI spraying; filling; draining), and how can these challenges be tackled?
- Is the BTI committee still **performing its tasks** as expected?

4.6 Community action to reduce mosquito breeding-6

Objective

- Interaction between BTI team and other villagers about the larval source management activities
- Participants stimulated to plan and implement further activities (start of rainy season).

Materials

- Visual-1

Course content

1. Remind participants about the **role of mosquitoes** in transmission of malaria parasites in our village. The **action plan** on larval source management aims to stop the breeding of malaria mosquitoes in and around our village.
2. Inquire from the participants whether there have been any **malaria cases** during the past month. If there are any cases, this is a sign that malaria mosquitoes are still present in our village.
3. Ask: Will **heavy rains** stimulate the breeding of mosquitoes?
4. Explain that mosquito larvae will get **washed away** by heavy rains, but the pools created by the rains will be a new source of mosquito breeding.
5. Mosquitoes will not be able to breed in water puddles which **dry up quickly** after the rain. Only water bodies that **remain for at least 2 weeks** can become productive breeding sites of mosquitoes. To understand this, we have to look at the mosquito's life-cycle.
6. Show **Visual-1**. Explain that it takes 11-15 days for the eggs to develop into larvae, pupae, and finally into adult mosquitoes (in hot weather it takes fewer days than in cold weather).

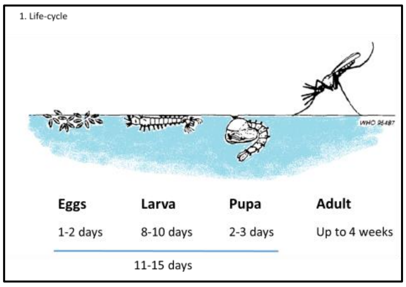

7. Ask: If rain creates **temporary pools** of stagnant water that disappear within 5 days, do we need to fill or drain these pools? (no, these pools cannot produce mosquitoes)
8. Explain that when **water dries up**, the larvae or pupae that were present in the water will die, and no mosquitoes will emerge.
9. Now ask the **HSA** to report on the number of confirmed malaria cases past month. This indicates whether malaria mosquitoes are active in the village.
10. Ask the committee members to take the floor and report on their monitoring activities of the **current situation**:
    a. Are breeding sites present in or around the village?
    b. Are Anopheles larvae found in those breeding sites?
    c. Are there any other possible sites within the village where malaria mosquitoes could be coming from? If yes, how could they be monitored and controlled?
    d. Does the committee need any help from villagers (or youth) in monitoring of Anopheles larvae? If yes, how could this be arranged?
11. Ask the committee members to report on their **larval source management** activities.
    a. How many breeding sites have been sprayed with BTI?
    b. When and how often was spraying done?
    c. Explain why BTI spraying will not be conducted during the rainy season.
    d. Are there currently water bodies which are suitable for filling or draining? If yes, discuss how this could be done.
12. Ask one or two villagers (not committee members) to describe how they have **contributed to larval source management** over the past month, and what challenges they faced.

Group discussion

- Discuss whether the situation of mosquito breeding will increase **when the rains start**, and how we can avoid this as much as possible.
- What should be the role of the community in **filling or draining** of breeding sites during the rainy season?
- Ask the participants to **evaluate** whether the committee members are performing their task as they should, or how this could be improved.

ANNEX 1. PARTICIPANTS LIST (LARVAL SOURCE MANAGEMENT)

Village name: **Date (dd-mm-yy)**

No.	Participant name	Gender	....-....-....	....-....-....	....-....-....	....-....-....	....-....-....	....-....-....	....-....-....
1									
2									
3									
4									
5									
6									
7									
8									
9									
10									
11									
12									
13									
14									
15									
16									
17									
18									
19									
20									
21									
22									
23									
24									
25									
26									
27									
28									
29									
30									
31									
32									
33									
34									
35									
36									
37									
38									
39									
40									
41									
42									
43									
44									
45									
46									
47									
48									
49									
50									

Village name: **Date (dd-mm-yy)**

No.	Participant name	Gender	….-….-….	….-….-….	….-….-….	….-….-….	….-….-….	….-….-….	….-….-….
51									
52									
53									
54									
55									
56									
57									
58									
59									
60									
61									
62									
63									
64									
65									
66									
67									
68									
69									
70									
71									
72									
73									
74									
75									
76									
77									
78									
79									
80									
81									
82									
83									
84									
85									
86									
87									
88									
89									
90									
91									
92									
93									
94									
95									
96									
97									
98									
99									
100									
