# Supplementary material for: Community-based malaria control in southern Malawi: a description of experimental interventions of community workshops, house improvement and larval source management
Source: Malar J. 2018 Jul 16;17:266. doi: 10.1186/s12936-018-2415-1 (PMC6048888; doi:10.1186/s12936-018-2415-1)
Supplement: Supplementary file 4 — Additional file 4. Manual on the use of BTI by village committee members for malaria prevention and control. [file 12936_2018_2415_MOESM4_ESM.pdf]

Additional file 4

Manual on the
use of *BTI* by village committee members

for malaria prevention
and control

Majete Malaria Project

Majete Malaria Project – Community-based malaria control in the perimeter of Majete Wildlife Reserve

Contents

PREFACE 2

1. INTRODUCTION 3

2. BASICS 5

2.1 WHAT IS BTI 5
2.2 HOW TO USE THE SPRAYER 6
2.3 WHEN TO USE FILLING, DRAINING, OR BTI 6
2.4 SURVEYING AND MAPPING OF BREEDING SITES 8

3. SPRAY OPERATION 10

3.1 HOW TO PREPARE FOR A SPRAY OPERATION 10
3.2 HOW TO CONDUCT A SPRAY ROUND 11
3.3 HOW TO CLEAN UP AFTER SPRAYING 13
3.4 HOW TO MAINTAIN AND STORE EQUIPMENT AND SUPPLIES 14
3.5 HOW TO EXAMINE WHETHER SPRAYING WAS EFFECTIVE 15

4. ORGANIZATION AND MANAGEMENT 16

4.1 ROLES OF LSM COMMITTEE 16
4.2 FEEDBACK AT VILLAGE WORKSHOPS 17
4.3 PREPARING AND MAINTAINING A WORK PLAN 17
4.4 RECORD KEEPING AND REPORTING 18
ANNEX 1. WORK PLAN CALENDAR 20
ANNEX 2. RECORDS OF BTI SPRAYING ACTIVITIES 21
ANNEX 3. MEASURING EFFECTIVENESS OF BTI 22
ANNEX 4. HABITAT TRACKING 23
ANNEX 5. INSTALLING THE SPRAYER 25
ANNEX 6. MAINTENANCE OF THE SPRAYER 26

PREFACE

This manual has been developed by the Majete Malaria Project, as a collaborative effort between its project partners. Drafting of the document was by Henk van den Berg, with inputs provided by Rob McCann, Steve Gowelo, Prosper Chaki, Alinune Kabaghe, Asante Kadama, Mackenzie Nkalapa, Saidon Banda, Monicah Mburu, Tumaini Malenga and Michele van Vugt.

This manual was first produced in May 2016. The manual is available in the English and Chichewa language. Funding for development of this manual was provided by Dioraphte Foundation, the Netherlands. Requests for permission to reproduce or translate this document – whether for sale or for non-commercial distribution – should be addressed to the programme manager (e-mail address: pmthrue@medcol.mw).

1. INTRODUCTION

This manual is the next supplement to the malaria manual, produced by the Majete Malaria Project in collaboration with The Hunger Project. This manual is meant for use **only in those villages** that have been **selected for larval source management** during the community ballot box event. This intervention is part of a research trial to study the effectiveness of new vector control interventions on malaria.

Larval source management (LSM)

We can best protect ourselves against the bites of malaria mosquitoes, by sleeping under a net. Bed nets are very effective against malaria and should be used every night by all members of the household.

But we can also do something extra to **attack the malaria mosquitoes at their source**. This will be by managing the water bodies, puddles, ponds and swamps in which the malaria mosquitoes like to breed. Mosquitoes depend on water for their life-cycle. Without suitable water they cannot exist.

Larval source management is an intensive strategy that can only work if the community actively participates throughout the year. During the **rainy season**, there can be a lot of malaria mosquitoes, and malaria usually peaks at this time. However, after the rainy season, and throughout the **dry season** there continue to be some malaria mosquitoes which manage to find some remaining water bodies in which to breed. These mosquitoes, even if they are few, can continue to circulate malaria in the community throughout the year. Therefore, we should manage the water bodies throughout the year, and attempt to break the transmission cycle of malaria.

Most water bodies could be filled or drained, so that water does not stay. In this way, any mosquito larvae initially available will die, and new larvae will not have water to develop at all. Some water bodies or swamps might be too big to be removed (drained or filled), or the water body might be important for use by the community. Malaria mosquitoes can continue to breed in these permanent water bodies even during the dry season. For these water bodies, there is another effective method available to kill mosquito larvae. This is the **spraying of BTI**, which is a safe, biological insecticide that kills only mosquito larvae.

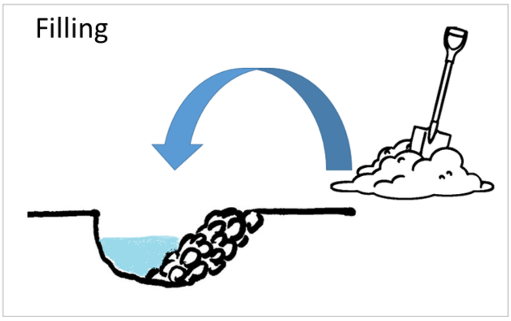

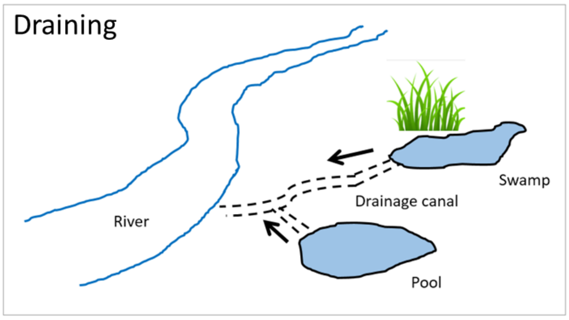

Hence, larval source management will have **two components**:

1. Removal of breeding sites by the community (filling, draining)
2. Spraying of BTI by the LSM committee

In this manual we will concentrate on the **second component**. However, it is important to know that two components should be carried out side-by-side so that mosquito breeding is stopped in all water bodies in and around our village.

Roles and responsibilities

Health animators should provide supervision to the LSM committee members in performing their tasks; and monitor their activities. Also, they should train the LSM committee members in the field on identifying the larvae of *Anopheles* and *Culex*.

Ten LSM committee members have been selected from each LSM village. All ten will be trained on BTI and should be committed to conduct surveys and weekly BTI applications. The LSM committee is responsible for all the activities related to BTI.

Refresher training and feedback sessions on BTI will be organized every 6 or 12 months during the coming two years. The performance in the village will be monitored.

The **village head** is a key person in the BTI activities. His role is to give **support in coordination, organization and planning** of all LSM activities, including BTI spraying. The health animator and LSM committee members should routinely inform the village head about the ongoing BTI activities, and request his support in problem solving.

The health surveillance assistant (**HSA**) is the key link between the village and the District Health Office, responsible for oversight of all health activities in the community. Her/his role is to advise the LSM committees, and to support health animators in explaining the use of BTI to the community.

THP-Malawi is responsible for selection, training and monitoring of LSM committee members, including the activities on BTI. The **Health and Education Unit** (HEU) of MoH is responsible for assisting in the training, and annual supervision. The **College of Medicine** is responsible for the provision of technical support, equipment and supplies to the LSM committee members.

How to use this manual

This manual is used for the purpose of training and for implementation. First, it is used for the training of the health animators and LSM committees. Subsequently, it is intended to guide the LSM committees during their seasonal activities.

The described methods are concise – focusing on the key points only. Each key point is discussed during the training workshops. Emphasis is on skills development and field practice.

The manual consists of three parts:

1. **Basics** on BTI;
2. Activities related to the **spray operation**; and
3. **Organizational aspects** of BTI application as a malaria control activity.

Each session starts with learning objectives, followed by a description of the key points, and ending with an exercise, which can be the field practice or questions for discussion.

2. BASICS

2.1 What is BTI

Learning objective

1. Participants understand the basic characteristics of BTI
2. Participants understand the role of BTI in malaria control

Key points

1. BTI is a **bacterium** naturally found in the soil.
2. The BTI product used in malaria control is known to kill mosquito larvae and is produced in large quantities so that the product you'll use is concentrated and of high quality.
3. BTI produces toxic proteins that **kill the larvae of mosquitoes**. BTI is only toxic to the larvae of mosquitoes and flies.
4. BTI does **not kill agricultural pests**, such as aphids or caterpillars. Therefore, BTI is useless for agriculture.
5. BTI is **safe for other animals**, such as bees, fish, shrimps and birds. These animals can swallow BTI without getting sick, or dying.
6. BTI is **safe for humans when applied in recommended concentrations**. It will not cause people to get sick, even if it is applied to drinking water.
7. But we must take care when handling pure BTI granules, because the granules are highly concentrated and may cause symptoms such as eye irritation.
8. After BTI is sprayed in the field, it **remains active for only a few days**. This is because BTI breaks down quickly in bright **sunlight**. For effective control of mosquitoes, it must be re-applied once every week (further details in later sections).
9. BTI is used **throughout the year**.
10. After a period of heavy rains, there may be many **temporary water bodies** which dry out within one week – this makes them unsuitable for mosquitoes to breed because larvae will die when the pool dries up. BTI should not be wasted on these temporary water bodies.
11. Heavy rains could also **wash the BTI away**, so it is better not to spray at times of heavy rains because it would be a waste of costly BTI product.
12. BTI spraying is important throughout the **dry season** when there are only **few water bodies** in which mosquitoes can breed. If we manage to find and control these last few water bodies, we can drastically reduce the number of malaria mosquitoes. This could break the transmission cycle of malaria parasites.

Exercise

1. Is it better for us to control mosquito breeding using BTI than a chemical pesticide, such as those used in cotton? Why, or why not?
2. Explain in your own words why BTI needs to be applied again every week.

2.2 How to use the sprayer

Learning objective

1. Participants understand the essential components of the sprayer
2. Participants able to assemble and disassemble the sprayer

Key points

1. The sprayer has many parts. The most important parts are the tank, pump, lance, hose and nozzle.
2. The tank is opened with the lid. Inside the lid there is a diaphragm, which serves as a filter. The filter will prevent particles from blocking the hose or nozzle.
3. The sprayer should not be used for chemical pesticides
4. Installing the sprayer is explained in Annex 5.

   Diagram: see sprayer operator's manual

Exercise

1. Study Annex 5.
2. Observe the parts and components of the sprayer, and explain their function and use.
3. Assemble and disassemble the main parts of the sprayer.

2.3 When to use filling, draining, or BTI

Learning objective

1. Participants understand and learn by heart the decision rules for use of filling, draining, and BTI.
2. Participants skilled to apply the decision rules to field situations.

Key points

1. By larval source management (LSM) we mean the use of **filling**, **draining** and **BTI** to control the breeding of malaria mosquitoes.
2. Smaller water bodies can easily be **filled with soil**, **stones, rubble** to permanently close them. Some larger water bodies can be **drained by digging a drainage canal** where there is a gradient toward lower ground.
3. **Land levelling** is a combination of draining and filling, aiming to prevent accumulations of water in the future.
4. Filling and draining should be routinely carried out by the community, with support from the **LSM committee**. This is one of the main tasks of the LSM committee.
5. Some water bodies cannot be filled or drained, either because they are too large for filling or draining, or because they have a purpose for keeping cattle or agricultural crops. BTI helps kill mosquito larvae in these water bodies.
6. Examples are:

a. Large swamps
b. Temporary floodplain pools near the river
c. Watering holes needed for cattle
d. Irrigation tanks and canals in agricultural crops

7. BTI is an **expensive** product, and its use should be limited to only those breeding sites that cannot be filled or drained.

8. The following chart shows how to make decisions on what to do with each water body.

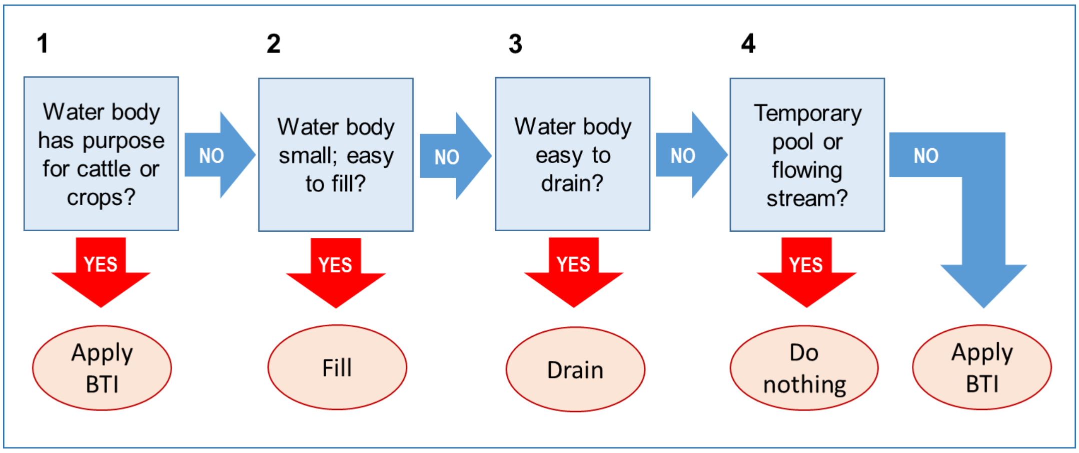

Box 2: When to use filling, draining or BTI?

Fill when:

- The water body does not have any purpose
- Is small and shallow
- Is more easily filled than drained
- When adequate soil, rubble or other filling materials are available on-site

Drain when:

- The water body does not have any purpose
- Drainage to nearby lower ground is feasible
- Is more easily drained than filled

Use BTI when:

- Water body has not yet been filled or drained
- Is not a 'temporary pool' that will quickly dry up (after rain showers)
- Water is not flowing but standing still

Exercise

1. Visit a wide range of water bodies and try to apply the decision criteria.

2. Discuss what to do when filling or draining is the best option (what next step should be taken?).

2.4 Surveying and mapping of breeding sites

Learning objective

1. Participants learn to use mapping as a planning tool for BTI spray operations.
2. Participants know how to explore the village environment for breeding sites.

Key points

1. Surveying and mapping of breeding sites is important because it helps us to understand **where and when** mosquito breeding occurs. This will help us tackle the problem.

Surveying – close examination of the environment (village)<br><br>Mapping – drawing of a map of the environment (village)

2. Surveying also helps us to find out **whether filling or draining is necessary** and where we target BTI spraying.
3. The **results** of the surveying exercise are used to draw and update the map.
4. **Mapping of the village environment** is a tool to help us planning where BTI spraying should be carried out.
5. The village proper plus a **400-meter wide zone** around the village is the area which should be covered by LSM (see Box 3). The border of this zone should be clear to those involved in LSM activities, and could be **indicated with sticks** or other markers. LSM activities should only be conducted within the border.

Box 3. Village and its border zone

Mosquitoes can fly 400 meters, and even further, which means that those breeding in swamps at 10 minutes walk distance from the village can still reach our houses (although many might not reach our houses). Those mosquitoes breeding closer to our houses are more likely to find us at night, because they are already inside the village.

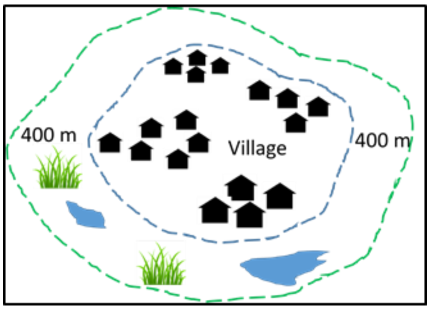

6. The **village map** developed during the village workshops on LSM should be used as example. The map should be regularly **updated** in the field while doing a field survey.
7. Once a month, **habitat tracking** should be conducted. Habitat tracking is the full survey of all the breeding sites that have been indicated on the map. This survey will take more time to conduct. The purpose of habitat tracking is to have a clear idea of the amount of BTI needed for a spray round. For habitat tracking, use the form in **Annex 4**, which includes a guideline on determining the amounts of water and BTI to use for the day. One form should be completed every month.
8. If recent rain showers have resulted in an increase in number or size of water bodies, a re-fill of the spray tank may be necessary to complete a spraying round.

9. Take **dipper samples** from the water bodies to determine the presence of *Anopheles* larvae. Water bodies with *Anopheles* larvae should receive priority in filling, draining or BTI spraying.

Box 4. Identifying mosquito larvae

It is assumed that LSM committee members can recognize *Anopheles* and *Culex* larvae. It is the task of the health animator to teach them to recognize the two types of larvae (Source: Modified from WHO).

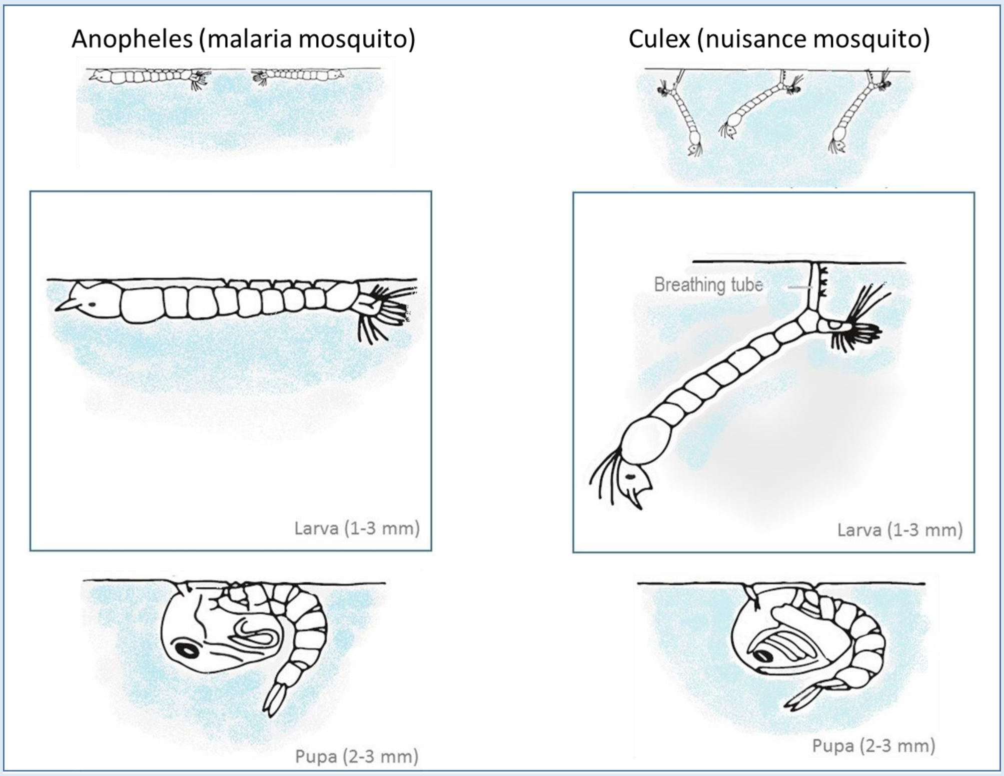

10. Water bodies in which **no *Anopheles* larvae** are found could still be important breeding sites, for example, if the mosquitoes have just laid their eggs, or when eggs are laid in a weeks' time.
11. Indicate on the map where there are water bodies that should be targeted for **filling or draining**, either by the committee or through involvement of the community. The more water bodies can be filled or drained, the less we need to use up the BTI supplies, and the less time we need to spend with weekly spraying of BTI.
12. Use the map for planning the **most efficient walking route** for doing the BTI spray operation of all remaining water bodies (those with and without larvae).
13. **Update** the map once a month, because water bodies will change.

Exercise

1. Prepare and update the village map with the locations of water bodies.
2. Has the 400-m zone around the village been marked? If not, how can this be achieved?
3. Use this map to make a plan for any further actions on filling and draining.
4. Use this map to make a plan for the BTI spray operations.

Notice

The Majete Malaria Project staff will occasionally visit the village to sample the breeding sites in the village and to check if there are any mosquito larvae. If there are still many larvae, this is an indication that BTI spraying has not been very effective. If that is the case, further action will be taken to help the LSM committee to improve their spraying operations.

3. SPRAY OPERATION

3.1 How to prepare for a spray operation

Learning objective

1. Participants able to make all steps in preparation of spraying.
2. Participants able to make the appropriate volume of BTI mix.

Procedure

1. Always try to **estimate the accurate volume of BTI mix** needed for one spray round, because any left-overs after completing the spray round will be waste. Do not mix more VectoBac® WG than can be used on that day. More than one tank may be needed to cover the water bodies.
2. VectoBac® WG can easily be **measured** without the use of a weighing scale. One kg of VectoBac® WG will fill 2 L of volume in a measuring jug.
3. To **prepare the spray mix**, follow the instructions in the Box below.
4. VectoBac® WG **suspends** readily in water and will stay suspended during normal application periods.
5. Brief recirculation may be necessary if the spray mixture has sat for several hours or longer. This is done by **gently shaking** the sprayer with its spray mix.
6. Although BTI when sprayed is safe for humans, the **concentrated product** can cause moderate **eye irritation** and allergic reactions. Therefore, it is important that during the handling and mixing of pure BTI product, contact with eyes is avoided, and breathing BTI dust is avoided.
7. **Wash hands with soap** after handling the product, and do not eat, drink or smoke when handling BTI product or when spraying in the field.

Box: Preparation of BTI mix

Take the standard measuring cup.

<u>If you plan to prepare a full tank with BTI mix (16 litres)</u>:

1. Fill the measuring cup with BTI, up to the marked line at the top. This is 140 grams of BTI (or about 400 ml by volume)
2. Add 16 litres of water, which is up to the maximum volume for the sprayer

3. Gently shake the spray mix

If you plan to prepare a half-full tank:

1. Fill the measuring cup with BTI, up to the marked line at the middle. This is 70 grams of BTI (or about 200 ml by volume)
2. Add 8 litres of water, which is half a tank
3. Gently shake the spray mix

Exercise

1. Recall all steps to prepare for the spraying operation.
2. Practice the preparation of the spray operation; in particular, practice the making of BTI mix.
3. Discuss the safety for the sprayer, and compare that to a farmer spraying his cotton field.

3.2 How to conduct a spray round

Learning objective

1. Participants understand the techniques and methods of spraying
2. Participants skilled to conduct a spray operation

Procedure

1. After the sprayer with BTI mix has been prepared, the spray round can begin. One person should carry the sprayer; the other keep records. They should switch tasks half-way the spray round.
2. A **record sheet** for recording the number of sprayed water bodies is shown in Annex 2.
3. During the spray round, there is **no time** to look for the presence of mosquito larvae. **All water bodies** present at this time should be sprayed.
4. Take the **most efficient walking route** on the village map, as determined in your work plan, visiting all water bodies within the demarcated zone around the village. Do not skip water bodies, unless they are very temporary pools (staying for less than 7 days) or flowing streams.
5. Mosquito larvae occur in **shallow water only** (up to 30 cm deep). Therefore, they should be sprayed at the shallow edge of large water bodies. Water deeper than 30 cm should not be sprayed.
6. Mosquito larvae like to hide around **reeds and grasses** growing in shallow water. When spraying the water where there are reeds or grasses, make sure that BTI reaches the water and is not ending up on the plants. This is best achieved by pushing the reeds to the side so that you can spray the water. If possible, you could also remove plants from the water edge. Only when BTI is present in the water it will be able to kill mosquito larvae.

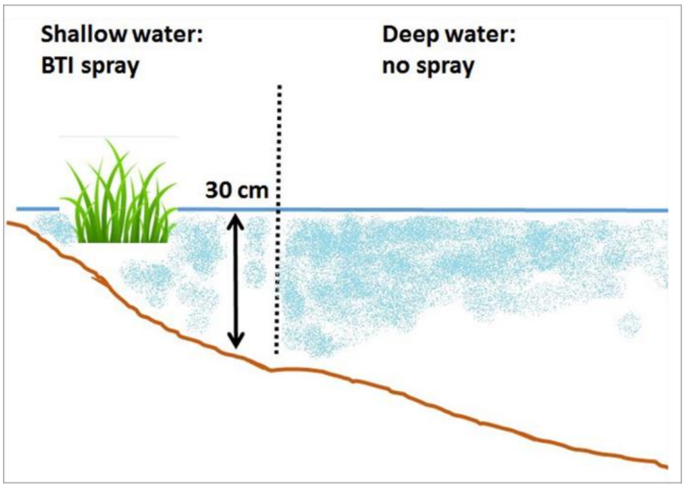

Large water bodies

7. When spraying large water bodies, apply BTI **only at the edge** in water that is no deeper than 30 cm.
8. **Walk at constant speed** and **swing the lance** to achieve a wide swath path and even distribution of BTI over the water surface. Avoid wasting BTI on the ground surface, on plants, or in deep water.

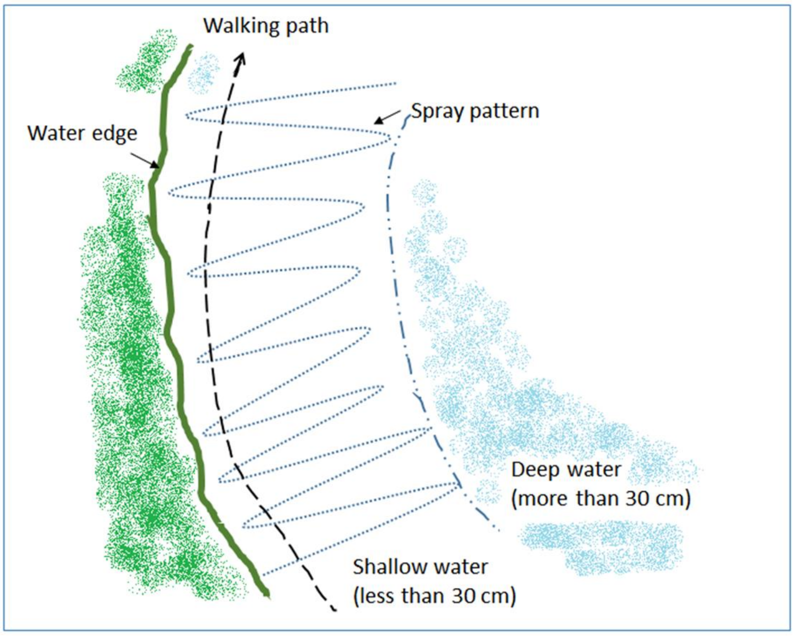

Smaller water bodies

9. Medium water bodies, that are less than 30 cm deep should be **covered entirely**.

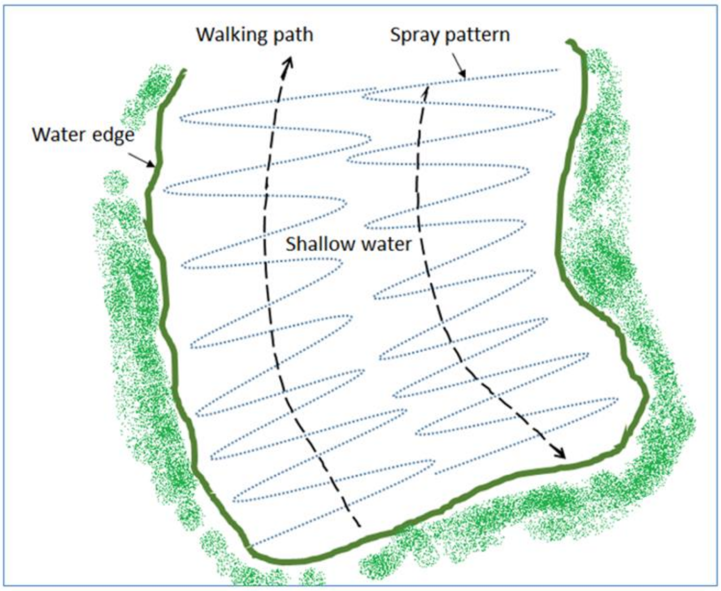

10. For small water bodies, use the **spot spray method**, which is by giving a very brief spray dose directly above the water body.
11. In case of cattle **hoof prints**, spray by oscillating the lance in your hand to cover the area of hoof prints. This is better than giving spot sprays to each individual hoof print.

General instructions

12. Avoid spraying at a time of **strong winds**, because most BTI will disappear with the wind.
13. Do not let the **nozzle** touch the soil because this could cause blockage.
14. After the spray mixture has sat for several hours or longer, **gently recirculate** the mixture in the tank.
15. When a **refill of the tank** is required during the spray round, go back to base to prepare the needed amount of extra BTI mix.

Exercise

1. Practice the spraying technique, swath width and walking speed, using a pressurized tank filled with water.
2. Practice the spraying operation in an area with different types of water bodies, including hoof prints.
3. <u>Where there are reeds and grasses in shallow water, how can we make sure that BTI reaches the water</u>?

3.3 How to clean up after spraying

Learning objective

1. Participants understanding the importance of a proper clean-up.
2. Participants skilled to conduct the clean-up.

Procedure

1. After completing a spray round, flush the tank and clean the sprayer, using water and some detergent.
2. **Immediate cleaning** after spraying is needed to prevent a hard layer of BTI coating onto the sprayer's components. Unused spray-mix should not be left in the sprayer overnight.
3. After releasing the pressure in the tank, any left-over spray mix can be **disposed in the field**, since BTI is not a poisonous pesticide.
4. Take note of the **amount of unused BTI mix**, and determine whether less BTI mix should be prepared for next time.
5. **Clean the nozzle, filters, and seals**. Cleaning the nozzle is essential to ensure smooth flow of spray mix through the nozzle (refer to Annex 6).
6. If a certain part has been **damaged** or worn out, immediately notify the health animator who should contact the EPO about the need for a spare part. The EPO should communicate this to both the THP Head of Programs and the MMP Site Coordinator.

Exercise

1. After the field practice of spraying, conduct the steps of clean-up, and check whether the sprayer is in good working condition.
2. What would happen when the clean-up is not conducted?

3.4 How to maintain and store equipment and supplies

Learning objective

1. Participants understand methods of safe storage and stock management.
2. Participants able to conduct basic maintenance of spray equipment.

Key points

1. Store the sprayer in a **dry and secure room**, away from children and animals.
2. Well before the stock of BTI is **running out**, inform the health animator, who should notify the EPO, that more supplies are needed.
3. **Do not contaminate** water, food, or feed by storage or disposal
4. The sprayer must be used **for BTI only**. Do not use the sprayer for agricultural insecticides. This is extremely important, because Bti is sprayed on water that may be used as drinking water by people or livestock. Agricultural insecticides are not safe to use on drinking water, so using the BTI sprayer for agricultural insecticides would harm people in your community. The LSM committee is responsible for ensuring the BTI sprayer is used only for its intended purpose.
5. The sprayer occasionally needs to be **lubricated**, and the cylinder needs to be opened and cleaned. These actions are explained in **Annex 6**.
6. The BTI granules (VectoBac® WG) must be stored in its original, properly **labeled container**, well sealed and stored in a cool, dry and well ventilated room.

7. Even though BTI is safe when sprayed in the field, the BTI granules are highly concentrated and could be **harmful** to the eyes, or when inhaled or ingested.
8. The BTI container should **not be reused**. Empty containers should be thoroughly washed with water and detergent. The empty containers are marked with a code and should be returned to MMP.

Exercise

1. Identify a safe storage room within the village, and a person responsible for storage.
2. Practice proper storage of spray equipment.
3. Discuss how to avoid a stock-out of BTI supplies?

3.5 How to examine whether spraying was effective

Learning objective

1. Participants able to evaluate the effect of spraying on mosquito larvae.

Key points

1. It is important to know soon after the spray round whether there has been a **killing effect on mosquito larvae**. Therefore, the LSM committee members should do spot checks.
2. BTI kills mosquito larvae not immediately, but **after a period of a number of hours** (2 - 24 hours).
3. **One day before the actual spray round**, conduct a <u>pre-spray survey</u>. To do this, identify **3 water bodies** where live mosquito larvae are present.
4. At each of the selected water bodies, **mark the location** where live mosquito larvae are present with a stick, so that the exact location can be retraced after two days.
5. Take 3 dipper samples from each location. Count the **number of live and dead larvae** before spraying in each dipper sample. Record the results on the data sheet (Annex 3)
6. **One day (24 hours) after spraying**, at the same time of the day as when spraying was conducted, revisit the 3 sites and check the locations that you marked with a stick.
7. Take 3 dipper samples from each location. Count the **number of live and dead larvae** after spraying in each dipper sample.
8. **Record** the results on your record form, shown in Annex 3. Ask advice or assistance from the health animator when needed.
9. **Draw a conclusion** from the results: Was the killing action of BTI ineffective, moderately effective, or highly effective?
10. Is any **follow-up action** needed? If BTI was ineffective, report this immediately to the health animator, or EPO, who should inform the MMP Site Coordinator and THP Director of Operations.

4. ORGANIZATION AND MANAGEMENT

4.1 Roles of LSM committee

Learning objective

1. Participants aware of their own role within the LSM committee.
2. Participants aware of the role of the LSM committee within the village.

Key points

1. The **LSM committee** has a crucial role in all LSM activities in the village.
2. The LSM committee **mobilizes villagers** to remove standing water through filling and draining, out to 400m from the last house in the village.
3. In addition, the LSM committee should **monitor the situation** on mosquito breeding throughout the year (Methods of surveying and mapping have been described in Section 2.4).
4. Now that some of the committee members have been trained on BTI, the committee is conducting **weekly BTI spraying** and managing the BTI equipment and supplies.
5. Also, the LSM committee members provides **feedback to the community** on all their activities during the village workshops on LSM, and occasionally organize field visits for the villagers.
6. The LSM committee must manage this range of activities, and therefore, it is important that committee members **support each other**. For example, the LSM members will need support from the other committee members in mobilization of villagers and conducting monthly surveying of breeding sites. They may also need support during the weekly BTI spray rounds.
7. To accomplish these tasks in an orderly and transparent manner, it is useful for the LSM committee to hold regular **committee meetings** to discuss all matters related to LSM.
8. The health animator, HSAs and village head have the role of **advisor and quality assurance** to the LSM committee.

Exercise

1. Discuss roles and responsibilities of committee members.
2. Draw up an agenda for a typical LSM committee meeting.

4.2 Feedback at village workshops

Learning objective

1. Participants able to give feedback on BTI spraying to villagers at village workshops.

Key points

1. In monthly village workshops on LSM, it is important for the LSM committee to report back on their activities to the community. The community needs to **understand** what is going on, and how they can **contribute** to LSM around their houses and within the village.
2. The LSM committee will inform the villagers about the **BTI spraying**. They should give a short demonstration of BTI spraying.
3. The LSM committee should also report on the **current situation** of mosquito breeding sites in the village. They will conduct a mapping exercise to visualize the breeding sites, and give an update every month.
4. Also, the LSM committee will give the villagers an update on the **implementation of LSM**:
    - How many breeding sites have been sprayed with BTI
    - When and how often was spraying done
    - Was the BTI effective at killing mosquito larvae
    - Are any of these water bodies suitable for filling or draining? If yes, discuss how this could be done.
5. On occasion, the health animator will request the LSM committee to **guide the community to the field** to nearby larval breeding sites to collect larvae and pupae of malaria mosquitoes, or to have a village walk along various breeding sites.

Exercise

1. Prepare a short presentation of the activities and results conducted in the past month.
2. Identify critical issues in which other villagers should be requested to assist or contribute.

4.3 Preparing and maintaining a work plan

Learning objective

1. Participants having planned their timeline of activities.
2. Participants using the work plan to guide their weekly activities.

Key points

1. BTI spraying will be conducted throughout the year.
2. The activities related to BTI are:
    a. BTI spray rounds

b. Surveying of breeding sites
c. Checking the killing effectiveness of BTI
d. Village workshops on LSM

3. BTI sprays are at **weekly intervals**. This is because BTI is not effective for more than a few days, and the life cycle of a mosquito can be completed in as little as 7 days.
4. The surveying, effectiveness checks, and LSM workshops are at **monthly intervals**.
5. Annex 1 shows a **calendar for work planning**. Every LSM committee should complete the work planning for each activity. It is good practice to have a fixed day each week for doing the BTI spraying, throughout the year.
6. Verify the work plan among the members of the **LSM committee** to get consensus.
7. Every week, **maintain and update the work plan**. Check the activities that have been completed, and prepare for the upcoming activities.

Exercise

1. Prepare (and update) an annual work plan until the end of the year.

4.4 Record keeping and reporting

Learning objective

1. Participants understanding how records should be kept.
2. Participants able to report to the health animator, and at village workshops.

Key points

1. The BTI activities require systematic record keeping.
2. First, the **work plan calendar** (shown in Annex 1) requires weekly updating.
3. Second, the **BTI spraying activities** require weekly recording (shown in Annex 2).
4. Third, monthly measurements of **effectiveness** of BTI can be recorded on the record sheet in Annex 3.
5. The work plan calendar (Annex 1) and BTI spraying activities (Annex 2) are the **most important**, and should be given due attention.
6. Record sheets should be **carefully kept** in a folder throughout the season.
7. LSM committee members can present the results to the community through their **presentations** at the monthly LSM village workshops.
8. Committee members should **report** their results to the health animator.
9. When **BTI supplies** are running out, or in case of any other problem regarding BTI, the health animator should be alerted, who will contact the MMP project when necessary.
10. MMP will send routine text messages to all BTI teams to remind them of the upcoming BTI application in the field.

Exercise

1. Explain the records on the three record sheets in a way that other villagers would understand.
2. Discuss the importance of reporting to the health animator and to MMP.

Annex 1. Work plan calendar

**Week**	Pre-spray survey		BTI spray		Testing BTI effectiveness		LSM workshop	Responsi-ble person
nr.	Planned date	Completed	Planned date	Completed	Planned date	Completed	Completed	
1								
2								
3								
4								
5								
6								
7								
8								
9								
10								
11								
12								
13								
14								
15								
16								
17								
18								
19								
20								
21								
22								
23								
24								
25								
26								

Annex 2. Records of BTI spraying activities

Week nr.	Date	Number of water bodies sprayed with BTI					
		**Small** (less than 2 m^2)		**Medium** (2 to 20 m^2)		[illegible] 20 m^2)	
		Tick	Total	Tick	Total	Tick	Total
1							
2							
3							
4							
5							
6							
7							
8							
9							
10							
11							
12							
13							
14							
15							
16							
17							
18							
19							
20							
21							
22							
23							
24							
25							
26							

Annex 3. Measuring effectiveness of BTI

Starting date	Before/ After	Count	Site-1			Site-2			Site-3		
			Sample: 1	2	3	Sample: 1	2	3	Sample: 1	2	3
	1 day **before** spraying	LIVE larvae:									
		DEAD larvae:									
	1 day **after** spraying	LIVE larvae:									
		DEAD larvae:									
	**Conclusion:**										

Starting date	Before/ After	Count	Site-1			Site-2			Site-3		
			Sample: 1	2	3	Sample: 1	2	3	Sample: 1	2	3
	1 day **before** spraying	LIVE larvae:									
		DEAD larvae:									
	1 day **after** spraying	LIVE larvae:									
		DEAD larvae:									
	**Conclusion:**										

Starting date	Before/ After	Count	Site-1			Site-2			Site-3		
			Sample: 1	2	3	Sample: 1	2	3	Sample: 1	2	3
	1 day **before** spraying	LIVE larvae:									
		DEAD larvae:									
	1 day **after** spraying	LIVE larvae:									
		DEAD larvae:									
	**Conclusion:**										

Starting date	Before/ After	Count	Site-1			Site-2			Site-3		
			Sample: 1	2	3	Sample: 1	2	3	Sample: 1	2	3
	1 day **before** spraying	LIVE larvae:									
		DEAD larvae:									
	1 day **after** spraying	LIVE larvae:									
		DEAD larvae:									
	**Conclusion:**										

Starting date	Before/ After	Count	Site-1			Site-2			Site-3		
			Sample: 1	2	3	Sample: 1	2	3	Sample: 1	2	3
	1 day **before** spraying	LIVE larvae:									
		DEAD larvae:									
	1 day **after** spraying	LIVE larvae:									
		DEAD larvae:									
	**Conclusion:**										

Annex 4. Habitat tracking

Monthly Habitat Tracking

Village: ____________________ Month: ___________

Habitat number (from map)	Habitat condition		Measured length with water (m)		Measured width with water (m)		Calculated area with water (m2)	Estimated depth (cm)		BTI applied? Yes/No	Is draining or filling possible? Yes/No
	Wet or dry?	vegetated or not?						At edge	At center		
				X		=					
				X		=					
				X		=					
				X		=					
				X		=					
				X		=					
				X		=					
				X		=					
				X		=					
				X		=					
				X		=					
				X		=					
				X		=					
				X		=					
				X		=					
				X		=					
				X		=					
				X		=					
				X		=					
				X		=					
					Total area (m2) :						

Determining amount of water and Bti to use for the day

Total calculated area for the day (m2)	Equivalent area as fraction (in football grounds)	Area, rounded up by 1/4 ground (in football grounds)	Water (L)	Bti (g)	Bti (ml)
**100**	1/50	1/4	4	35	100
**500**	1/10	1/4	4	35	100
**1000**	1/5	1/4	4	35	100
**1250**	1/4	1/4	4	35	100
**1500**	3/10	1/2	8	70	200
**1875**	3/8	1/2	8	70	200
**2000**	4/10	1/2	8	70	200
**2500**	1/2	1/2	8	70	200
**3000**	6/10	3/4	12	105	300
**3125**	5/8	3/4	12	105	300
**3500**	7/10	3/4	12	105	300
**3750**	3/4	3/4	12	105	300
**4000**	4/5	1	16	140	400
**4375**	7/8	1	16	140	400
**4500**	9/10	1	16	140	400
**5000**	1	1	16	140	400

Annex 5. Installing the sprayer

(Source: Adapted from Operator's manual, Jacto Inc.®)

1. Installing the shaft: Insert the shaft (1) in the free opening on the tank base (on the same side of the chamber). Fit the shaft lock (2).

   Diagram: see sprayer operator's manual

2. Installing the level: Lightly greases the orifice on the chamber top. Fit a flat washer on the shaft and rod. For easy assembly, install the lever on the shaft and the rod on the chamber top at the same time. Install the other flat washers on the shaft and rod and lock with the lock ring.

   Diagram: see sprayer operator's manual

3. Attaching the lance to the trigger valve: Insert the lance into the trigger valve and secure it with the screw cap.

   Diagram: see sprayer operator's manual

4. Tightening the fastening nut: Before using the equipment, tighten the fastening nut (A). IMPORTANT: This operation must be done by hand. Do not use any type of tool.

   Diagram: see sprayer operator's manual

5. Adjusting the shoulder strap: The shoulder straps can be quickly adjusted to properly position the sprayer on the operator's back.

   Diagram: see sprayer operator's manual

6. After assembling the sprayer, fill the tank with clean water and pressure--check the tank lid, diaphragm, tank bottom, lance and trigger valve for leakage. Most leaks can be stopped by retightening the appropriate connections and fittings. Any leakage must be repaired before returning to service

Annex 6. Maintenance of the sprayer

(Source: Adapted from Operator's manual, Jacto Inc.)

After finishing the application, clean and wash all equipment in an approved decontamination area.

Diagram: see sprayer operator's manual

Remove all chemicals before storing the sprayer. Chemical products have different reactions and can damage the sprayer components and environment as well as cause personal injury.

Lubrication

Once every month (every 40 hours of spraying), clean the chamber and lubricate the components, as shown. Remove the chamber and lubricate the piston cups. Preferably, use graphite-base grease. Silicone and vaseline are also acceptable for lubrication. Do not apply grease excessively to avoid clogging and impurities from building up.

Diagram: see sprayer operator's manual

Cylinder maintenance

Remove the pumping rod from the chamber. Loosen the gasket holder and remove the assembly. Remove the chamber (A) from the cylinder (B). Clean the ball (C) and the piston cup (D); replace them if necessary.

Diagram: see sprayer operator's manual

Troubleshooting

Put about 1/2 gallon of water in the tank. While operating the pumping lever, look into the tank to investigate the problem. Refer to the table and diagram below.

**Problem:**	**Causes:**	**Corrections:**
Leakage through the cylinder upper part	Worn or dried out piston cup	Replace or lubricate the piston cup
After pumping and pressurizing the chamber, as you operate the lever it drops fast	Cylinder valve not sealing properly due to wear or impurities	Clean or replace the cylinder valve
After pumping and pressurizing the chamber, the lever rises slowly when released	Chamber valve not sealing properly due to wear or impurities	Clean or replace the valve
